# Wellbeing-focused media literacy interventions in secondary schools: A systematic review and meta-analysis protocol

**DOI:** 10.1136/bmjopen-2026-119555

**Published:** 2026-06-16

**Authors:** Maria Tibbs, Gill Francis, Jennifer Mountain, Gianfranco Polizzi, Beth T Bell

**Affiliations:** 1 Psychology in Education Research Centre, Department of Education, University of York, York, UK; 2 Department of Linguistics and Communication, University of Birmingham, Birmingham, UK

**Keywords:** Meta-Analysis, Systematic Review, MENTAL HEALTH, Schools, Social Media

## Abstract

**Introduction:**

Media and digital technologies shape adolescent development across interconnected online and offline contexts, offering opportunities for learning and connection while also exposing young people to risks that can affect wellbeing. Wellbeing-focused media literacy interventions aim to support young people in understanding and managing how media and technology shape their experiences and wellbeing. Schools are well placed to deliver such interventions at scale through curricular integration. However, existing interventions vary widely in focus, design and evaluation quality making it difficult to determine what works and why. This review synthesises school-based wellbeing-focused media literacy interventions and evaluates their effectiveness across well-being outcomes.

**Methods and analysis:**

A range of interdisciplinary databases (ACM Digital Library, British Education Index, CINAHL, ERIC, MEDLINE, PsycINFO, Scopus, Web of Science) alongside targeted grey literature searches of internal evaluation reports and dissertations were searched on 20 January 2026. Eligible studies will include school-based interventions for adolescents aged 11–18. Abstracts and full texts will be screened in Covidence, and relevant data extracted using an adapted Template for Intervention Description and Replication (TIDieR) framework. A three-level random effects meta-analysis will estimate pooled effects across psychological, social, physical and digital wellbeing domains. Narrative synthesis, informed by the adapted TIDieR framework, will explore intervention content, delivery and theoretical foundations to identify mechanisms of change and sources of heterogeneity.

**Ethics and dissemination:**

Ethical approval is not required for this systematic review, as it will only include published data. The review findings will be disseminated through a peer-reviewed publication and conference presentations.

**PROSPERO registration number:**

CRD420251269724.

**STRENGTHS AND LIMITATIONS OF THIS STUDY**

- Uses a three-level random effects meta-analysis that accounts for dependency among multiple effect sizes within studies, allowing more accurate synthesis of complex intervention outcomes.
- Comprehensive interdisciplinary search across psychology, education, public health and human computer interaction databases alongside grey literature sources to maximise coverage of relevant interventions.
- Integrates interventions targeting well-being outcomes associated with media and technology use including psychological, social, physical and digital well-being within a single media literacy framework, helping consolidate previously fragmented areas of research.
- Intervention characteristics will be systematically coded using an adapted Template for Intervention Description and Replication framework to identify intervention components and mechanisms of change.
- Substantial heterogeneity in intervention content, theoretical frameworks, delivery formats and outcome measures may still limit comparability across studies despite the use of a multilevel meta-analytical approach and use of subgroup analyses.

## Introduction

Rates of mental health difficulties among adolescents have risen globally in recent decades,^1–3^ accompanied by declines in feelings of happiness and positive affect.^4–6^ Most mental health difficulties emerge before the age of 25,^7^ and in the UK one in five adolescents were reported to have a probable mental health disorder in 2023.^8^ At the same time, there has been a rapid growth in youth digital technology use, with adolescents in the UK spending an average of 4–6 hours per day online.^9^ While these trends have unfolded concurrently, existing evidence suggests that technologies are not inherently harmful to young people, but rather associations between digital technology use and mental health are complex and shaped by a wide range of social, developmental and contextual factors.

For contemporary adolescents, social life, learning and self-expression are increasingly shaped by media and digital technologies, including social media platforms such as YouTube, TikTok, Snapchat and WhatsApp.^10–12^ These technologies provide opportunities for connection, creativity and community. However, they can also expose young people to cyberbullying, harmful or distressing content, reinforce sedentary behaviours and promote upward comparison, all of which may undermine mental health and wellbeing.^8 13–17^ Supporting young people to develop capable and informed engagement with media and technology is therefore a pressing public health and educational concern.^18^

### Defining Wellbeing

Wellbeing is commonly defined as ‘how people feel and how they function, both on a personal and a social level and how they evaluate their lives as a whole’^19^ [p.6], though its conceptualisation and measurement vary considerably across the literature.^20 21^ It is widely recognised as a multidimensional construct, encompassing psychological, physical and social domains.^22^ Psychological wellbeing includes two components; subjective (hedonic) wellbeing, such as life satisfaction and affect balance^23 24^ and psychological (eudaimonic) wellbeing, which focuses on qualities associated with flourishing, such as purpose and personal growth.^25 26^ Physical wellbeing is frequently indexed through engagement in health-promoting behaviours (eg, physical activity^27^) and avoidance of health-risk behaviours (eg, smoking and substance use).^28^ Social wellbeing captures the extent to which individuals feel integrated into and supported by their social networks and communities, encompassing experiences of belonging, acceptance and positive relationships.^29^ wellbeing, at both the personal and social level, can also be measured using more objective indicators, including physical/mental health status, educational attainment, socio-economic security and housing quality.^30^

As young people’s everyday lives have become deeply intertwined with digital technologies, scholars have increasingly argued that well-being frameworks must also incorporate a digital dimension. Digital wellbeing has been defined as the ‘subjective individual experience of optimal balance between the benefits and drawbacks obtained from mobile connectivity’^31^ (p.938). Promoting wellbeing in contemporary society therefore requires approaches that attend to multiple, inter-related dimensions of wellbeing and equip young people with the knowledge and skills to engage with media and technology in ways that support personal and social flourishing in an increasingly digital world.

### Media Literacy Education

Media literacy education offers one potential pathway for promoting wellbeing among adolescents. Media literacy refers to the ability to access, analyse, evaluate and create media, with an emphasis on critically understanding media messages and their underlying intentions.^32 33^ As media environments have become increasingly digitised, this framing has been extended to include digital literacy, applying the same core functional, critical and socioemotional competencies to online media, platforms and technologies, alongside related literacies such as information and artificial intelligence (AI) literacy.^34 35^ Drawing on this perspective, digital literacy can be typically understood as a context-specific subfield of media literacy that encompasses the same underlying competencies. These include functional competencies such as searching for information, creating content and navigating platforms^36^ and critical competencies such as evaluating online content and understanding how platform infrastructures and algorithms shape what users encounter and produce.^37–39^

Accordingly, in this review we use the term *media literacy interventions* as an umbrella to refer to interventions grounded in media literacy frameworks and traditions, including those applied to digital contexts (eg, AI literacy, social media literacy) and commonly described as digital literacy interventions. Increasingly, these competencies are viewed not only as educational outcomes in their own right, but also as important determinants of young people's wellbeing. In line with this perspective, there is growing recognition that media literacy includes an integral wellbeing dimension, involving the socioemotional knowledge and skills needed to navigate the psychological demands of modern-day media and digital technology and to understand how these experiences can affect wellbeing.^40^ Emerging evidence suggests that these functional, critical and socioemotional skills are needed to help young people use technology in ways that maintain, enhance, support and protect wellbeing.^41–44^

## Wellbeing-focused Media Literacy nterventions

Schools have become a key setting for initiatives aimed at improving young people’s media literacy in relation to wellbeing, as they reach learners during formative developmental periods, enable integration into curricula, and allow delivery at scale.^45^ In the United Kingdom (UK), this importance is reflected in government efforts to improve media and digital skills,^46^ as well as mandated education on some online harms through the Relationships, Sex and Health Education curriculum.^47^

As media literacy has become an established educational priority, a diverse range of interventions aim to foster media literacy in ways that support youth wellbeing in both online and offline contexts. For example, interventions have been developed to promote positive body image by fostering media literacy in relation to harmful appearance-related messaging across media and technology, while also teaching psychological and behavioural coping strategies.^48 49^ Interventions have also been developed to promote social wellbeing in online spaces by providing education related to how the design features and social affordances of online media environments may shape and sustain cyberbullying behaviours and social relationships.^50 51^ While these interventions vary in terms of content and approach, a commonality is that all aim to *develop the knowledge and skills needed to foster wellbeing in relation to media and technology* through three interrelated sets of competencies:

1. *Critical* knowledge and skills to analyse and evaluate media and technology.
2. *Functional* knowledge and skills to navigate and operate media and technology.
3. *Socioemotional* knowledge and skills to recognise, reflect on, and manage emotional and social responses to media and technology.

In many cases, wellbeing is positioned as an assumed or downstream outcome arising from the development and application of these competencies, rather than as an explicit focus of intervention theory or evaluation. We refer to this diverse group of approaches as *wellbeing-focused media literacy interventions*.

Despite growing recognition of their educational and public health relevance,^52^ little is known about which wellbeing-focused media literacy interventions work, for whom and why. Existing reviews have largely examined intervention effectiveness within specific domains, such as cyberbullying^53^ and body image,^54^ even though interventions often share elements that promote critical awareness, self-reflection and behavioural change.^55^ This fragmentation prevents understanding of shared mechanisms or ‘active ingredients’ that drive positive outcomes across intervention types and limits understanding of what approaches are most effective or transferable to real-world educational settings.

## Objectives

Accordingly, this systematic review aims to identify classroom-based wellbeing-focused media literacy interventions that are effective among adolescents aged 11–18. Furthermore, it aims to examine the components and characteristics that underpin effectiveness. Specifically, it addresses the following questions:

1. Do school-based well-being-focused media literacy interventions improve youth well-being outcomes (psychological/subjective, social, physical and digital)?
2. What active ingredients (components and characteristics) underpin effective school-based well-being focused media literacy interventions?

## Materials and Method

This study will conduct a systematic review and meta-analysis examining the effectiveness of eligible interventions. The review will include experimental and quasi-experimental studies, including dissertations employing these designs, provided they meet the predefined eligibility criteria. Where sufficient comparable data are available, quantitative findings from eligible studies will be synthesised using meta-analysis to estimate the effects of the interventions under investigation.

## Protocol and egistration

The current protocol is aligned with the Preferred Reporting Items for Systematic Reviews and Meta-Analyses Protocols (PRISMA-P)^56^ see online supplemental materials. The subsequent systematic review and meta-analysis will be reported following the PRISMA guidelines.^57^

10.1136/bmjopen-2026-119555.supp1 Supplementary data

This protocol has been preregistered on the Open Science Framework Platform (OSF; 10.17605/OSF.IO/APM7F) and PROSPERO (CRD420251269724). Any significant amendments made to the protocol will be documented and reported in the final systematic review manuscript. Database searches have been conducted, and title and abstract screening is currently ongoing, with completion anticipated by November 2026.

## Information Sources and Search trategy

The systematic search included both peer-reviewed and grey literature published in English, with no geographical restrictions. Peer-reviewed studies were identified through interdisciplinary databases to ensure comprehensive coverage across education, psychology, public health and human–computer interaction: ACM Digital Library, British Education Index, CINAHL, ERIC (Education Resources Information Centre), MEDLINE, PsycINFO, Scopus, Web of Science. Grey literature included doctoral theses and publicly available internal reports evaluating interventions with randomised designs, as these sources can provide valuable, otherwise unpublished evidence subjected to some level of independent scrutiny. Doctoral theses were identified through ProQuest Dissertations and Theses Global. All database searches were conducted on 20 January 2026. In addition to these interdisciplinary databases, websites of reputable media literacy and/or educational evaluation organisation websites (eg, Education Endowment Foundation, Internet Matters, PSHE Association) will be searched for relevant internal evaluation reports. The rationale for including each database is presented in table 1. To ensure all relevant articles will be captured, a process of forward reference and backward citation checking using *citationchaser*^58^ will be conducted on eligible full texts.

**Table 1:** Rationale for bibliographic databases

Database	Rationale
ERIC (Education Resources Information Centre)	Captures literature focused on education and learning contexts.
British Education Index	Covers educational literature focused within the British context that may not be captured within ERIC.
Web of Science	Broad, multidisciplinary database indexing a wide range of high-impact peer-reviewed research.
PsycINFO	Specialises in psychology and related behavioural sciences.
CINAHL	Covers nursing, allied health and applied healthcare research.
MEDLINE	Biomedical and public health database; includes extensive coverage of health interventions and uses MeSH terms, enhancing search precision.
SCOPUS	Indexes a broad range of journals across health sciences, technology and education.
ACM Digital Library	Houses studies from human–computer interaction, a field that often contributes to research on digital literacy and technology use.
ProQuest Dissertations and Theses Global	Grey literature database that houses doctoral theses.

MeSH, Medical Subject Headings.

The search strategy was developed in collaboration with a library specialist at the University of York and through examination of previous systematic reviews in the areas of digital literacy, media literacy and wellbeing. Search terms focused on the core domains under investigation: the population of interest, characteristics of the intervention, relevant outcomes, the setting within which interventions are implemented and study design, as well as MeSH terms or subject areas relevant to each database where applicable. Considering the breadth of potential wellbeing outcomes, the search strategy was conducted separately for each of the four wellbeing domains (psychological/subjective, social, physical and digital) to ensure sufficient and relevant retrieval of studies. See online supplemental material 1 for an example SCOPUS search strategy.

10.1136/bmjopen-2026-119555.supp2 Supplementary data

## Eligibility riteria

To be included in the current systematic review, all studies, including those identified across the grey literature, will need to meet the predefined inclusion and exclusion criteria as informed by the Population, Intervention, Comparison, Outcome, Study Design model. For a summary of the inclusion criteria, see table 2.

**Table 2:** Inclusion/exclusion criteria

	Inclusion	Exclusion
Population	Young people aged 11–18 attending postprimary, secondary or high school.	Young people not attending postprimary, secondary or high school. Young people aged <11 or >18.
Intervention	School or classroom-based media literacy interventions that include education on the knowledge and skills needed to foster well-being in relation to media and technology including critical, functional and socioemotional.	Interventions that: Are not conducted within education settings. Do not target students’ knowledge and skills related to media and technology.
Comparator	Studies will be included if they compare the intervention with either a passive control (eg, no intervention, usual care or waitlist/delayed care) or active control (eg, an alternative intervention or the same intervention delivered at a different time point).	Studies without a comparison or control group (eg, pre–post, uncontrolled or observational designs) will be excluded.
Outcome	Studies will be included if their primary or secondary outcomes relate to either well-being and/or digital/media/information literacy.	Studies will be excluded if they do not assess well-being as an outcome
Study Design	Studies will be included if they: Employ experimental designs with a comparator group, RCTs (including cluster-RCTs) and non-randomised comparison group designs (e.g. quasi-experiments using matched control or non-equivalent control group). Include a quantitative comparison between intervention and control or comparison conditions to evaluate effectiveness or efficacy.	Studies will be excluded if they: Do not include a comparator group (e.g. single-group pre–post, purely observational or cross-sectional studies). Employ qualitative designs. Are systematic reviews and meta-analyses. Are intervention protocols without published findings available at the time of synthesis.
Time frame	2000 to present.	Studies published before the year 2000.

RCTs, randomised controlled trials.

### Population

To be included in this review, studies must focus on young people engaged in formal second level education aged 11–18. This age range was selected to capture developmental stages typically corresponding to secondary-level schooling across international education systems, while excluding primary and postsecondary contexts.

### Intervention

Eligible interventions must include a media literacy component, defined as educational content focused on developing adolescents’ ability to access, analyse, evaluate and create media, with an emphasis on critically understanding media messages and their underlying intentions.^32 33^ Media or technology must be an active target of the intervention, rather than solely the mode of delivery.

Interventions may contain one or more of the following media literacy components, which reflect different ways in which well-being-focused media literacy is operationalised in practice:

1. *Critical*: The knowledge and skills needed to critically analyse and evaluate media and technology through a well-being lens (eg, understanding persuasive intent, platform design, algorithms or how media features shape experiences relevant to well-being).
2. *Functional*: The knowledge and skills needed to navigate and operate media and technology in ways that may support well-being, including modifying engagement, using safety and support features, and enacting boundaries or help-seeking behaviours within digital environments.
3. *Socioemotional*: The knowledge and skills needed to recognise and reflect on emotional and social responses to media and technology, and to evaluate when media engagement supports or undermines well-being.

### Comparators

Studies will be included if they evaluate the intervention against either a passive control condition (eg, no intervention, usual care or waitlist/delayed care) or an active control condition (eg, an alternative intervention or the same intervention delivered at a different time point). Studies that do not include a comparison or control group, such as pre–post, uncontrolled or purely observational designs, will be excluded.

### Outcomes

The primary outcome is wellbeing, which we define broadly as ‘how people feel and how they function, both on a personal and a social level, and how they evaluate their lives as a whole’^19^ (p.6). To structure our analysis, we draw on the WHO’s three-domain model of well-being, which includes psychological, social and physical dimensions.^22^ We extend this framework by adding a fourth domain: digital well-being. This expanded model allows us to capture the full spectrum of experiences and risks associated with young people’s digital lives. For the purposes of this review, we define these four domains as follows:

1. *Psychological/subjective wellbeing* reflects individuals’ evaluations and lived experiences of their own lives, including their sense of life satisfaction, experiences of positive and negative emotions, self-concept, the absence of mental health difficulties and overall capacity to flourish.^59 60^
2. *Social wellbeing* refers to the extent to which individuals feel integrated into and supported by their social networks and communities.^29^ This can include concepts such as belonging, friendship quality, as well as experiences of social isolation and loneliness.
3. *Physical wellbeing* can be described as the ability to maintain bodily health and energy through balanced behaviours such as exercise, diet, sleep, sexual health and avoidance of harmful habits.^61 62^
4. *Digital wellbeing* refers to individuals’ ability to engage with digital technologies in ways that support well-being, including feeling safe, in control and supported in online environments, as well as minimising exposure to digital risks such as harmful content, excessive use or negative social interactions.

Secondary outcomes include indicators of digital, media and information literacy, reflecting young people’s knowledge, skills and critical competencies in navigating online environments. For detail on the categorisation and representative measures, see online supplemental materials 2 and 3).

### Study Design

This review will include English language, peer-reviewed experimental and quasi-experimental studies with a comparator or control group, including randomised controlled trials (cluster and individual) and non-randomised comparison designs (eg, matched or non-equivalent control groups). Eligible studies must provide a quantitative comparison between intervention and control conditions to assess effectiveness or efficacy.

### Timeframe

The search period was set from 2000 to the present date to capture developments in digital literacy during a transitional period of conceptual overlap with traditional media literacy, when technologies were beginning to be integrated into media literacy education and regulation.^63^ These developments are exemplified by the enactment of international policies such as the US Children’s Internet Protection Act (2000), the UK Communications Act (2003) and the European Union Action Plan for a Safer Internet (1999–2004), which increasingly framed media literacy around online safety, responsible use and digital participation alongside traditional media.

## Data anagement

Search results will be exported to Zotero reference managers and duplicates will be removed. Covidence systematic review software will be used to screen the abstracts and full texts of identified studies. Microsoft Excel will be used to extract relevant study data and to conduct quality appraisals of all included studies.

## Selection of Studies and Data Extraction

Following the removal of duplicates, studies will be screened in two stages using Covidence. First, titles and abstracts will be assessed against the inclusion and exclusion criteria. Full texts of potentially relevant studies will then be retrieved and reviewed for eligibility. Four reviewers experienced in systematic review methodologies will contribute to the data screening and extraction process. At each stage studies will be double screened, and discrepancies will be resolved through a joint discussion by the two reviewers, and the opinion of a third reviewer will be obtained if agreement is not reached. Inter-rater reliability (eg, Cohen’s κ) will be calculated to assess agreement between the two reviewers at title and abstract screening as well as full-text screening.

Similarly, two reviewers will duplicate data extraction and coding to ensure accuracy and consistency. One reviewer will extract and code all included studies, while the remaining three reviewers will each extract and code a proportion of the studies to enable cross-checking and verification. Extracted data will be compared for accuracy and completeness, with any discrepancies resolved through discussion and arbitration by a fourth author where necessary.

A prepiloted data extraction form will be used to capture bibliographic details, sample characteristics, intervention and comparison conditions, and quantitative outcomes from each included study. The form will capture:

1. *Key study characteristics*: including author details, study date, country, study aims and objectives.
2. *Participant and school characteristics*: including sample size and composition (eg, gender distribution, mean age, type of school (public/private) and contextual factors such as rural/urban setting of the school).
3. *Intervention characteristics*: We will record intervention characteristics using an adapted version of the Template for Intervention Description and Replication (TIDieR) framework.^64^ Using this, we will extract details on:
  - The rationale, goal and theoretical framing of the intervention.
  - What is delivered (specific activities and intervention components). This will be extended to capture which well-being-focused media literacy components are targeted. These components include: (1) critical knowledge and skills; (2) functional knowledge and skills; and (3) socioemotional knowledge and skills. We acknowledge that these domains are interrelated and will not be mutually exclusive. The coding framework will therefore allow for dual or multiple categorisations where components span domains.
  - Who the intervention is delivered by (eg, teachers, trained facilitators, peers).
  - How the intervention was delivered (in-class online modules, group activities, presentations). This will be extended to extract details on mechanisms through which these interventions are theorised to achieve their outcomes. Specifically, we will code for teaching strategies and pedagogical approaches (eg, reflection, discussion, experiential learning) and behaviour change techniques (BCTs^65^) used to enact behaviour change.
  - Where the intervention was delivered (eg, within subject specific classes, after school, during pastoral care).
  - The frequency and duration of the intervention session(s).
  - Evidence of any modifications during the process of intervention implementation.
  - Details on adherence to any existing protocols.
  - Details of any adaptations or tailoring for different contexts.
4. *Outcomes*: including outcome domains, measures used, time points of assessment (eg, pre, post, follow-up), directionality of well-being outcomes (positive or negative) and available relevant quantitative data (means, related effect sizes and associated variance estimates) for meta-analytical calculations.

## Quality Appraisal and Certainty of Evidence

To examine risk of bias across studies (including those identified through grey literature searching), the Cochrane Risk of Bias Tool (RoB2^66^) will be used. This tool evaluates the risk of bias by assigning studies a quality rating of low, high or unclear across several domains of bias. Studies deemed low quality will be retained for both the systematic review and meta-analysis. However, in line with recent meta-analyses of school-based interventions,^67 68^ sensitivity analyses will be conducted excluding low-quality studies to examine their influence on the magnitude and robustness of the overall effect. Funnel plots will be used to assess potential publication bias, and Egger’s test will be employed to statistically evaluate the presence of small-study effects.

## Data Synthesis

### Narrative synthesis

Extracted data will be tabulated and narratively synthesised following Popay *et al*’s^69^ guidance on narrative synthesis. First, study, participant and intervention characteristics will be summarised descriptively using the adapted TIDieR framework. Second, interventions will be compared according to their wellbeing-focused media literacy components (knowledge/understanding and skills) and the mechanisms of change identified (teaching strategies, pedagogies and BCTs). Finally, the synthesis will explore how variations in these components and mechanisms relate to wellbeing outcomes across domains (psychological/subjective, social, physical, digital). Using this approach, the synthesis will provide an overview of patterns in intervention content (what) and the mechanisms to enact behaviour change (how), providing a working theory of how the intervention works to achieve its outcomes.^70^

### Meta-analysis

All studies meeting the inclusion criteria will be included in the systematic review; however, only studies reporting sufficient quantitative data on well-being outcomes (eg, means and SD, change scores or effect estimates with variance) will be eligible for inclusion in the meta-analysis. Studies lacking extractable data or reporting outcomes that cannot be meaningfully standardised will be synthesised narratively. Where outcomes are reported on continuous scales, effect sizes are expected to be calculated as standardised mean differences (Hedges’ g), which is commonly used in meta-analyses of school-based interventions to accommodate heterogeneous measures and small sample sizes. Effect sizes will be interpreted in relation to those reported in prior meta-analyses of school-based interventions, with emphasis on robustness and practical relevance rather than fixed magnitude thresholds.

We will perform a meta-analysis using an intention-to-treat approach, as opposed to a per-protocol approach, to minimise post-randomisation bias and overestimation of effects.^71^ This means that all participants originally allocated to intervention and control groups will be analysed according to their initial assignment, regardless of adherence or dropout, ensuring a more conservative and unbiased estimate of effect. To account for probable correlation and dependence between multiple effect sizes reported within individual studies, we will adopt a three-level random-effects meta-analysis to estimate pooled effects. A three-level random-effects meta-analysis partitions variance into three components:

1. Sampling variance associated with each observed effect size (sampling error within studies).
2. Variance between effect sizes within the same study.
3. Variance between study effects.

In this analysis, effect sizes are nested within studies, and multiple outcomes reported by the same study are modelled as correlated at level two. This approach allows the inclusion of several non-independent outcomes per study while appropriately accounting for their clustering.^72 73^

To assess what proportion of the observed variance reflects variance in true effects, the *I^2^* statistic will be calculated, with cut-offs of 25, 50 and 75% denoting low, moderate and high levels of heterogeneity. 95% CIs for the *I^2^* values will be calculated using the method outlined by Borenstein.^74^ Finally, to explore sources of heterogeneity, we will conduct moderator analyses. Moderators will include study design characteristics (eg, passive vs active control conditions, publication year), intervention characteristics (eg, mode of delivery, number and duration of modules or lessons) and participant characteristics (eg, gender composition). In addition, outcome domains (psychological/subjective, social, physical, digital) will be entered as categorical moderators alongside study-level characteristics such as intervention design and participant details. This will allow us to formally test whether effects differ across outcome domains, as well as to examine potential sources of between-study heterogeneity. Following guidance from Weisz *et al*,^75^ analyses with categorical moderators will only be conducted if each category contains at least five cases.

As a robustness check, we will fit a correlated-hierarchical effects model, which combines a hierarchical variance structure with robust variance estimation to permit inference without requiring known within-study correlations.^76^ This will be accompanied by further sensitivity analyses using examination of the potential impact of outliers and risk of bias. Where substantial or unforeseen heterogeneity is identified, and where sufficient data are available, we may additionally conduct separate three-level or standard meta-analyses within well-being domains (eg, psychological/subjective, social, physical, digital) to improve conceptual and methodological comparability of pooled estimates. Importantly, meta-analytical decisions will be guided by theoretical coherence, measurement comparability and the distribution of available data within each domain. Where measures within a domain are conceptually or operationally heterogeneous, outcomes that cannot be meaningfully compared will be synthesised narratively rather than pooled.

Statistical analyses will be conducted using the *metaSEM* package^77^ in R statistical software.^78^

## Ethics and Dissemination

Ethical approval is not required for this systematic review, as it will only include published data. The review findings will be disseminated through a peer-reviewed publication and conference presentations.

## Public and Patient Involvement

The current protocol has been presented and reviewed by *n*=10 key stakeholders including representatives from charities and advocacy groups, education specialists, methodological experts and academic researchers as well as *n*=2 young people.

## Deviations from the Protocol

Any deviations from the registered protocol will be updated and recorded on the OSF and PROSPERO websites.

### Strengths and Limitations

This review has several methodological and conceptual strengths. Existing systematic reviews have largely examined the effectiveness of interventions targeting specific online harms,^53 79 80^ yet few have integrated these within a broader wellbeing framework 81. To date, no review has examined the effectiveness of wellbeing-focused media literacy interventions, no meta-analysis has synthesised the effectiveness of such interventions, and no evidence map has visualised the extent of the evidence base and associated research gaps. By synthesising evidence across school-based interventions that aim to develop young people's understanding of how digital experiences affect wellbeing and the skills needed to support it, this review addresses an important gap in the literature. The three-level meta-analysis will provide pooled estimates across psychological/subjective, social, physical, and digital wellbeing outcomes, while the accompanying narrative synthesis will identify intervention components and contextual factors associated with effectiveness. Findings will contribute to a clearer conceptualisation of wellbeing-focused media literacy and inform its implementation and evaluation in educational settings

Methodologically, the review is strengthened by preregistration on the Open Science Framework and PROSPERO and adherence to PRISMA-P guidance, supporting reproducibility and methodological rigour. The search strategy spans multiple interdisciplinary databases across education, psychology, public health and human–computer interaction, alongside grey literature sources, increasing the likelihood of identifying a comprehensive body of relevant evidence. Conceptually, the review integrates interventions targeting well-being outcomes associated with media and technology use including psychological, social, physical and digital well-being within a single media literacy framework. This approach helps consolidate previously fragmented areas of research. In addition, intervention characteristics will be systematically coded using an adapted TIDieR framework, enabling the identification of active components and mechanisms that may underpin effective well-being-focused media literacy interventions.

However, some limitations should be acknowledged. Considerable heterogeneity is also anticipated in intervention content, theoretical foundations, delivery formats and outcome measures across studies. Although this heterogeneity will be explicitly accounted for using a three-level random-effects meta-analytic model that accommodates dependency among multiple effect sizes within studies, variation in intervention design and measurement may still limit comparability and complicate interpretation of pooled estimates. Finally, the strength of the conclusions will depend on the methodological quality and reporting practices of the included primary studies.

## Supplementary Material

Reviewer comments

Author's manuscript

## References

[1] Cybulski L, Ashcroft DM, Carr MJ, et al. Temporal trends in annual incidence rates for psychiatric disorders and self-harm among children and adolescents in the UK, 2003-2018. BMC Psychiatry 2021;21:229. 10.1186/s12888-021-03235-w 33941129 PMC8092997

[2] Fitzgerald A, Mahon C, Shevlin M, et al. Exploring changing trends in depression and anxiety among adolescents from 2012 to 2019: Insights from My World repeated cross-sectional surveys. Early Interv Psychiatry 2025;19:e13562. 10.1111/eip.13562 38956877 PMC11730113

[3] Mojtabai R, Olfson M. National Trends in Mental Health Care for US Adolescents. JAMA Psychiatry 2020;77:703–14. 10.1001/jamapsychiatry.2020.0279 32211824 PMC7097842

[4] Blanchflower DG, Bryson A, Xu X. The declining mental health of the young and the global disappearance of the unhappiness hump shape in age. PLoS One 2025;20:e0327858. 10.1371/journal.pone.0327858 40864616 PMC12385385

[5] Dumuid D, Singh B, Brinsley J, et al. Trends in Well-Being Among Youth in Australia, 2017-2022. JAMA Netw Open 2023;6:e2330098. 10.1001/jamanetworkopen.2023.30098 37606925 PMC10445194

[6] Marquez J, Long E. A Global Decline in Adolescents’ Subjective Well-Being: a Comparative Study Exploring Patterns of Change in the Life Satisfaction of 15-Year-Old Students in 46 Countries. Child Indic Res 2021;14:1251–92. 10.1007/s12187-020-09788-8 PMC7611680 34539933

[7] Solmi M, Radua J, Olivola M, et al. Age at onset of mental disorders worldwide: large-scale meta-analysis of 192 epidemiological studies. Mol Psychiatry 2022;27:281–95. 10.1038/s41380-021-01161-7 34079068 PMC8960395

[8] NHS Digital. Mental Health of Children and Young People in England, 2023 - wave 4 follow up to the 2017 survey. 2023.

[9] Ofcom. Online nation report, 2023. Available: https://www.ofcom.org.uk/siteassets/resources/documents/research-and-data/online-research/online-nation/2023/online-nation-2023-report.pdf?v=368355

[10] Charmaraman L, Hodes R, Richer AM. Young sexual minority adolescent experiences of self-expression and isolation on social media: cross-sectional survey study. JMIR Ment Health 2021;8:e26207. 10.2196/26207 34524107 PMC8482247

[11] Goodyear VA, Armour KM. Young People’s health-related learning through social media: What do teachers need to know? Teach Teach Educ 2021;102:103340. 10.1016/j.tate.2021.103340 34083866 PMC8091040

[12] Mittmann G, Woodcock K, Dörfler S, et al. “TikTok Is My Life and Snapchat Is My Ventricle”: A Mixed-Methods Study on the Role of Online Communication Tools for Friendships in Early Adolescents. J Early Adolesc 2022;42:172–203. 10.1177/02724316211020368

[13] Al-Jbouri E, Volk AA, Spadafora N, et al. Friends, followers, peers, and posts: adolescents’ in-person and online friendship networks and social media use influences on friendship closeness via the importance of technology for social connection. Front Dev Psychol 2024;2:1419756. 10.3389/fdpys.2024.1419756

[14] Caba-Machado V, Mcilroy D, Padilla-Adamuz FM. Night-time use of electronic devices, fear of missing out, sleep difficulties, anxiety, and well-being in UK and Spain: a cross-cultural comparison. Curr Psychol 2024;43:21134–45. 10.1007/s12144-024-05934-5

[15] Engberg E, Leppänen MH, Sarkkola C, et al. Physical Activity Among Preadolescents Modifies the Long-Term Association Between Sedentary Time Spent Using Digital Media and the Increased Risk of Being Overweight. Journal of Physical Activity and Health 2021;18:1105–12. 10.1123/jpah.2021-0163 34326270

[16] Peppler K, Dahn M. Social media and creativity. In: The routledge international handbook of children, adolescents, and media. Routledge, 2022.

[17] Zheng Q, Yang W, Li T, et al. Examining the Pathways to Social Media FOMO: Mediation Effects of Passive Engagement and Upward Social Comparisons. Int J Human–Computer Interact 2025;41:15506–13. 10.1080/10447318.2025.2499156

[18] Büchi M. Digital well-being theory and research. New Media Soc 2024;26:172–89. 10.1177/14614448211056851

[19] Michaelson J, Mahony S, Schifferes J. Measuring wellbeing: A guide for practitioners. New Econ Found Lond 2012.

[20] Dodge R, Daly A, Huyton J, et al. The challenge of defining wellbeing. Intnl J Wellbeing 2012;2:222–35. 10.5502/ijw.v2i3.4

[21] Hone L, Schofield G, Jarden A. Conceptualizations of wellbeing: Insights from a prototype analysis on New Zealand workers. N Z J Hum Resour Manag 2015;15:97–118.

[22] World Health Organization. Summary reports on proceedings minutes and final acts of the international health conference held in New York from 19 June to 22 July 1946. 1946.

[23] Diener E, Suh EM, Lucas RE, et al. Subjective well-being: Three decades of progress. Psychol Bull 1999;125:276–302. 10.1037/0033-2909.125.2.276

[24] Diener E, Ryan K. Subjective Well-Being: A General Overview. South Afr J Psychol 2009;39:391–406. 10.1177/008124630903900402

[25] Ryan RM, Deci EL. On Happiness and Human Potentials: A Review of Research on Hedonic and Eudaimonic Well-Being. Annu Rev Psychol 2001;52:141–66. 10.1146/annurev.psych.52.1.141 11148302

[26] Ryff CD, Singer BH. Know Thyself and Become What You Are: A Eudaimonic Approach to Psychological Well-Being. J Happiness Stud 2008;9:13–39. 10.1007/s10902-006-9019-0

[27] Hennessey A, MacQuarrie S, Petersen KJ. Exploring physical, subjective and psychological wellbeing profile membership in adolescents: a latent profile analysis. BMC Psychol 2024;12:720. 10.1186/s40359-024-02196-5 39633504 PMC11619419

[28] Dittmar H, Bond R, Hurst M, et al. The relationship between materialism and personal well-being: A meta-analysis. J Pers Soc Psychol 2014;107:879–924. 10.1037/a0037409 25347131

[29] Keyes CLM. Social Well-Being. Soc Psychol Q 1998;61:121. 10.2307/2787065

[30] Organisation for economic co-operation and development secretary-general, OECD iLibrary. How’s life?: measuring well-being. 2011.

[31] Vanden Abeele MMP. Digital Wellbeing as a Dynamic Construct. Commun Theory 2021;31:932–55. 10.1093/ct/qtaa024

[32] Hobbs R. Digital and media literacy: a plan of action. A white paper on the digital and media literacy recommendations of the knight commission on the information needs of communities in a democracy. Aspen Institute, 2010.

[33] Aufderheide P. Media literacy: from a report of the national leadership conference on media literacy. Routledge Media Literacy Around the World; 1997.

[34] Leaning M. An Approach to Digital Literacy through the Integration of Media and Information Literacy. MaC 2019;7:4–13. 10.17645/mac.v7i2.1931

[35] Wuyckens G, Landry N, Fastrez P. Untangling media literacy, information literacy, and digital literacy: A systematic meta-review of core concepts in media education. JMLE 2022;14:168–82. 10.23860/JMLE-2022-14-1-12

[36] van Deursen AJAM, Helsper EJ, Eynon R. Development and validation of the Internet Skills Scale (ISS). Inf Commun Soc 2016;19:804–23. 10.1080/1369118X.2015.1078834

[37] Buckingham D. Digital Media Literacies: Rethinking Media Education in the Age of the Internet. Res Comp Int Educ 2007;2:43–55. 10.2304/rcie.2007.2.1.43

[38] Dezuanni M. Minecraft and children’s digital making: implications for media literacy education. Learn Media Technol 2018;43:236–49. 10.1080/17439884.2018.1472607

[39] Polizzi G. Internet users’ utopian/dystopian imaginaries of society in the digital age: Theorizing critical digital literacy and civic engagement. New Media Soc 2023;25:1205–26. 10.1177/14614448211018609

[40] Tuamsuk K, Subramaniam M. The current state and influential factors in the development of digital literacy in Thailand’s higher education. ILS 2017;118:235–51. 10.1108/ILS-11-2016-0076

[41] Gordon CS, Ferber K, Notley T, et al. The relationship between media literacy and well-being: A systematic review and meta-analysis. Educational Research Review 2025;49:100731. 10.1016/j.edurev.2025.100731

[42] Kalmus V, Tambaum T, Abuladze L. Use of digital tools, digital skills and mental well-being. 2023.235–50.

[43] Livingstone S, Mascheroni G, Stoilova M. The outcomes of gaining digital skills for young people’s lives and wellbeing: A systematic evidence review. New Media Soc 2023;25:1176–202. 10.1177/14614448211043189

[44] Vissenberg J, De Coninck D, Mascheroni G, et al. Digital Skills and Digital Knowledge as Buffers Against Online Mis/Disinformation? Findings from a Survey Study Among Young People in Europe. Soc Media Soc 2023;9:20563051231207859. 10.1177/20563051231207859

[45] Carta MG, Fiandra TD, Rampazzo L, et al. An Overview of International Literature on School Interventions to Promote Mental Health and Well-being in Children and Adolescents. CPEMH 2015;11:16–20. 10.2174/1745017901511010016 PMC4378068 25834625

[46] DCMS. Department for digital, culture, media and sport: annual report and accounts. 2022.

[47] Department for Education. Teaching online safety in schools. 2023.

[48] Bell BT, Taylor C, Paddock D, et al. Digital Bodies: A controlled evaluation of a brief classroom‐based intervention for reducing negative body image among adolescents in the digital age. Br J Educ Psychol 2022;92:280–98. 10.1111/bjep.12449 34350594

[49] Gordon CS, Jarman HK, Rodgers RF, et al. Outcomes of a cluster randomized controlled trial of the SoMe social media literacy program for improving body image-related outcomes in adolescent boys and girls. Nutrients 2021;13:3825. 10.3390/nu13113825 34836084 PMC8674763

[50] Kutok ER, Dunsiger S, Patena JV, et al. A Cyberbullying Media-Based Prevention Intervention for Adolescents on Instagram: Pilot Randomized Controlled Trial. JMIR Ment Health 2021;8:e26029. 10.2196/26029 34524103 PMC8482167

[51] Schoeps K, Villanueva L, Prado-Gascó VJ, et al. Development of Emotional Skills in Adolescents to Prevent Cyberbullying and Improve Subjective Well-Being. Front Psychol 2018;9:2050. 10.3389/fpsyg.2018.02050 30416471 PMC6212595

[52] Ofcom. A positive vision for media literacy: ofcom’s three-year media literacy strategy. 2024.

[53] Ng ED, Chua JYX, Shorey S. The Effectiveness of Educational Interventions on Traditional Bullying and Cyberbullying Among Adolescents: A Systematic Review and Meta-Analysis. Trauma Violence Abuse 2022;23:132–51. 10.1177/1524838020933867 32588769

[54] Zuair AA, Sopory P. Effects of Media Health Literacy School-Based Interventions on Adolescents’ Body Image Concerns, Eating Concerns, and Thin-Internalization Attitudes: A Systematic Review and Meta-Analysis. Health Commun 2022;37:20–8. 10.1080/10410236.2020.1813954 32873082

[55] Weinstein E, James C. School-based initiatives promoting digital citizenship and healthy digital media use. Handb Adolesc Digit Media Use Ment Health 2022. 10.1017/9781108976237.020

[56] Moher D, Shamseer L, Clarke M, et al. Preferred reporting items for systematic review and meta-analysis protocols (PRISMA-P) 2015 statement. Syst Rev 2015;4:1. 10.1186/2046-4053-4-1 25554246 PMC4320440

[57] Page MJ, McKenzie JE, Bossuyt PM, et al. The PRISMA 2020 statement: an updated guideline for reporting systematic reviews. BMJ 2021;372:n71. 10.1136/bmj.n71 33782057 PMC8005924

[58] Haddaway NR, Grainger MJ, Gray CT. Citationchaser: A tool for transparent and efficient forward and backward citation chasing in systematic searching. Res Synth Methods 2022;13:533–45. 10.1002/jrsm.1563 35472127

[59] Adler A, Unanue W, Osin E, et al. Psychological wellbeing. Happiness 2017;118.

[60] Stone AA, Mackie C. Introduction. In: Subjective well-being: measuring happiness, suffering, and other dimensions of experience. National Academies Press (US), 2013. 24432436

[61] Harvard University Health Sciences. Physical – Center for Wellness and Health Promotion, Available: https://wellness.huhs.harvard.edu/physical

[62] Hung S-T, Cheng Y-C, Wu C-C, et al. Examining Physical Wellness as the Fundamental Element for Achieving Holistic Well-Being in Older Persons: Review of Literature and Practical Application in Daily Life. J Multidiscip Healthc 2023;16:1889–904. 10.2147/JMDH.S419306 37435298 PMC10329914

[63] Wallis R, Buckingham D. Media literacy: the UK’s undead cultural policy. Int J Cult Policy 2019;25:188–203. 10.1080/10286632.2016.1229314

[64] Hoffmann TC, Glasziou PP, Boutron I, et al. Better reporting of interventions: template for intervention description and replication (TIDieR) checklist and guide. BMJ 2014;348:g1687. 10.1136/bmj.g1687 24609605

[65] Michie S, Richardson M, Johnston M, et al. The Behavior Change Technique Taxonomy (v1) of 93 Hierarchically Clustered Techniques: Building an International Consensus for the Reporting of Behavior Change Interventions. ann behav med 2013;46:81–95. 10.1007/s12160-013-9486-6 23512568

[66] Higgins J. Cochrane handbook for systematic reviews of interventions. Version 5.1. 0. 2011. Available: www.cochrane-handbook.org

[67] Hodder RK, O’Brien KM, Lorien S, et al. Interventions to prevent obesity in school-aged children 6-18 years: An update of a Cochrane systematic review and meta-analysis including studies from 2015-2021. EClinicalMedicine 2022;54:101635. 10.1016/j.eclinm.2022.101635 36281235 PMC9581512

[68] Zhang Q, Wang J, Neitzel A. School-based Mental Health Interventions Targeting Depression or Anxiety: A Meta-analysis of Rigorous Randomized Controlled Trials for School-aged Children and Adolescents. J Youth Adolesc 2023;52:195–217. 10.1007/s10964-022-01684-4 36229755 PMC9560730

[69] Popay J, Roberts H, Sowden A, et al. Guidance on the conduct of narrative synthesis in systematic reviews. Prod ESRC Methods Programme Version 2006;1:b92. 10.13140/2.1.1018.4643

[70] Rodgers M, Sowden A, Petticrew M, et al. Testing methodological guidance on the conduct of narrative synthesis in systematic reviews: effectiveness of interventions to promote smoke alarm ownership and function. Evaluation 2009;15:49–73. 10.1177/1356389008097871

[71] McCoy CE. Understanding the Intention-to-treat Principle in Randomized Controlled Trials. West J Emerg Med 2017;18:1075–8. 10.5811/westjem.2017.8.35985 29085540 PMC5654877

[72] Cheung M-L. Modeling dependent effect sizes with three-level meta-analyses: a structural equation modeling approach. Psychol Methods 2014;19:211–29. 10.1037/a0032968 23834422

[73] Van den Noortgate W, López-López JA, Marín-Martínez F, et al. Three-level meta-analysis of dependent effect sizes. Behav Res Methods 2013;45:576–94. 10.3758/s13428-012-0261-6 23055166

[74] Borenstein M, Cooper H, Hedges L, et al. Effect sizes for continuous data. Handb Res Synth Meta-Anal 2009;2:221–35.

[75] Weisz JR, Kuppens S, Ng MY, et al. What five decades of research tells us about the effects of youth psychological therapy: A multilevel meta-analysis and implications for science and practice. American Psychologist 2017;72:79–117. 10.1037/a0040360 28221063

[76] Pustejovsky JE, Tipton E. Meta-analysis with Robust Variance Estimation: Expanding the Range of Working Models. Prev Sci 2022;23:425–38. 10.1007/s11121-021-01246-3 33961175

[77] Cheung M-L. metaSEM: an R package for meta-analysis using structural equation modeling. Front Psychol 2015;5:1521. 10.3389/fpsyg.2014.01521 25601849 PMC4283449

[78] Core Team R. R: a language and environment for statistical computing [software].

[79] Guest E, Zucchelli F, Costa B, et al. A systematic review of interventions aiming to promote positive body image in children and adolescents. Body Image 2022;42:58–74. 10.1016/j.bodyim.2022.04.009 35679652

[80] Malinauskas R, Malinauskiene V. A meta-analysis of psychological interventions for Internet/smartphone addiction among adolescents. J Behav Addict 2019;8:613–24. 10.1556/2006.8.2019.72 31891316 PMC7044583

[81] Cowling M, Sim KN, Orlando J, et al. Untangling Digital Safety, literacy, and Wellbeing in School activities for 10 to 13 Year Old Students. Educ Inf Technol 2025;30:941–58. 10.1007/s10639-024-13183-z

