# Supplementary material for: Wellbeing-focused media literacy interventions in secondary schools: A systematic review and meta-analysis protocol
Source: BMJ Open. 2026 Jun 16;16(6):e119555. doi: 10.1136/bmjopen-2026-119555 (PMC13295985; doi:10.1136/bmjopen-2026-119555)
Supplement: Supplementary data [file bmjopen-16-6-s002.pdf]

Supplementary Material

Supplement 1. *Example SCOPUS Search String.*

Wellbeing Domain	Search Strategy
Psychological/Subjective Wellbeing:	TITLE-ABS-KEY ((adolescen* OR teen* OR student* OR pupil* OR youth OR "young pe*") AND (“digital skill*" OR "digital safety" OR "online safety" OR "online resilience" OR "digital resilience" OR "digital competenc*” OR "internet safety" OR "social media literacy" OR "digital literacy" OR "media literacy" OR "information literacy” OR "AI literacy” OR misinformation OR "social media" OR "digital media" OR "social network*" OR online) AND ("mental health" OR "mental illness" OR "mental disorder*" OR "psychological distress" OR wellbeing* OR "mental wellbeing" OR "life satisfaction" OR affect* OR "emotional wellbeing" OR "psychological wellbeing" OR resilience OR happiness OR optimism OR "personal growth" OR "self expression" OR identity OR suicid* OR "self-

Tibbs M, *et al. BMJ Open* 2026; 16:e119555. doi: 10.1136/bmjopen-2026-119555

	harm" OR depress* OR anxi* OR stress OR internali?* OR externali?* OR "self esteem" OR "self concept" OR "self worth" OR "body image" OR "eating disorder*" OR "disordered eating")<br><br>AND<br><br>("secondary school" OR "high school" OR "middle school" OR "post primary" OR "secondary education" OR classroom* OR "school-based" OR "whole school")<br><br>AND<br><br>(experiment* OR randomi?ed OR "randomi?ed controlled trial*" OR "randomi?ed control trial*" OR rct OR "cluster rct" OR "cluster randomi?ed*" OR "quasi-experiment*" OR evaluation OR pretest OR posttest OR "pre test" OR "post test" OR lesson* OR intervention* OR effectiveness)
Social Wellbeing	TITLE-ABS-KEY ((adolescen* OR teen* OR student* OR pupil* OR youth OR "young pe*”)<br><br>AND

	(“digital skill*" OR "digital safety" OR "online safety" OR "online resilience" OR "digital resilience" OR "digital competenc*” OR "internet safety" OR "social media literacy" OR "digital literacy" OR "media literacy" OR "information literacy” OR "AI literacy” OR misinformation OR "social media" OR "digital media" OR "social network*" OR online)<br><br>AND<br><br>(loneliness OR "social isolation" OR "social connect*" OR belonging OR friendship OR "social support" OR "peer rejection”)<br><br>AND

	("secondary school" OR "high school" OR "middle school" OR "post primary" OR "secondary education" OR classroom* OR "school-based" OR "whole school")<br><br>AND<br><br>(experiment* OR randomi?ed OR "randomi?ed controlled trial*" OR "randomi?ed control trial*" OR rct OR "cluster rct" OR "cluster randomi?ed*" OR "quasi-experiment*" OR evaluation OR pretest OR posttest OR "pre test" OR "post test" OR lesson* OR intervention* OR effectiveness)
Physical Wellbeing	TITLE-ABS-KEY ((adolescen* OR teen* OR student* OR pupil* OR youth OR "young pe*”)<br><br>AND

	(“digital skill*" OR "digital safety" OR "online safety" OR "online resilience" OR "digital resilience" OR "digital competenc*” OR "internet safety" OR "social media literacy" OR "digital literacy" OR "media literacy" OR "information literacy” OR "AI literacy” OR misinformation OR "social media" OR "digital media" OR "social network*" OR online)<br><br>AND<br><br>(“physical wellbeing" OR "physical well-being" OR sleep* OR "sedentary behav*" OR exercise OR "substance abuse" OR "substance use" OR "physical activ*" OR "health behav*" OR "sexual health")<br><br>AND<br><br>("secondary school" OR "high school" OR "middle school" OR "post primary" OR "secondary education" OR classroom* OR "school-based" OR "whole school")

	AND<br><br>(experiment* OR randomi?ed OR "randomi?ed controlled trial*" OR "randomi?ed control trial*" OR rct OR "cluster rct" OR "cluster randomi?ed*" OR "quasi-experiment*" OR evaluation OR pretest OR posttest OR "pre test" OR "post test" OR lesson* OR intervention* OR effectiveness)
Digital Wellbeing	TITLE-ABS-KEY ((adolescen* OR teen* OR student* OR pupil* OR youth OR "young pe*”)<br><br>AND<br><br>(“digital skill*" OR "digital safety" OR "online safety" OR "online resilience" OR "digital resilience" OR "digital competenc*” OR "internet safety" OR "social media literacy" OR "digital literacy" OR "media literacy"

	OR "information literacy” OR "AI literacy” OR misinformation OR "social media" OR "digital media" OR "social network*" OR online)<br><br>AND<br><br>(“digital well*" OR "online well*" OR cyberbull* OR bully* OR victimis* OR victimiz* OR harass* OR aggress* OR "online abuse" OR sext* OR groom* OR "harmful content" OR "violent content" OR "self-harm content" OR "hate speech" OR extremis* OR radicalis* OR radicaliz* OR "problematic internet*” OR "problematic social media*" OR "internet addiction" OR "social media addiction" OR "problematic smartphone*" OR "technology overuse" OR "gaming addiction" OR "problematic gaming" OR "online gambling" OR "screen time" OR "digital fatigue" OR "information overload" OR "fear of missing out" OR fomo OR pornograph* OR porn*)

	AND<br><br>("secondary school" OR "high school" OR "middle school" OR "post primary" OR "secondary education" OR classroom* OR "school-based" OR "whole school")<br><br>AND<br><br>(experiment* OR randomi?ed OR "randomi?ed controlled trial*" OR "randomi?ed control trial*" OR rct OR "cluster rct" OR "cluster randomi?ed*" OR "quasi-experiment*" OR evaluation OR pretest OR posttest OR "pre test" OR "post test" OR lesson* OR intervention* OR effectiveness)

Supplement 2. *Categorisation of Wellbeing Constructs for the Current Review**

**Wellbeing Domain**	**Direction of Indicator**	**Example & Definition**	**Example Representative Measures**
Psychological and Subjective Wellbeing	Positive Indicators	Life Satisfaction: Measures that assess satisfaction with life overall.	Satisfaction with Life Scale (Diener et al., 1985).
		Positive Affect: Measures that assess how much positive affect the individual experiences in their life.	PANAS–Positive Affect (Watson et al., 1988).
		Composite Wellbeing Measures which assess an individual's overall subjective wellbeing	Warwick Edinburgh Mental Wellbeing Scale (Tennant et al., 2007).

		Resilience<br>Measures which assess the ability to adapt and/or recover.	Adolescent Psychological Resilience Scale (APRS; Bulut et al., 2013).
		Optimism<br>Measures general expectations about positive outcomes.	Revised Life Orientation Test (LOT-R; Scheier et al., 1994).
		Quality of Life<br>Assesses overall satisfaction and fulfilment across life domains.	The Quality-of-Life Scale (QOLS; Burckhardt et al., 2003).
		Body Satisfaction<br>Measures contentment with various aspects of their body.	The Body Satisfaction Scale (Slade et al., 1990).
		Body Esteem<br>Measures positive self-evaluations of	The Body Esteem Scale (Franzoi & Shields, 1984).

		one's body and appearance.	
		Self Esteem<br>Measures an individual's overall sense of self-worth and value.	Rosenberg self-esteem scale (Rosenberg, 2011).
	Negative Indicators	DSM-V defined mental health outcomes (e.g., depression, anxiety, stress)<br>Measures which assess mental health outcomes as defined by the DSM-V.	Beck Depression Inventory (Beck, 1961).<br>State–Trait Anxiety Inventory (Spielberger, 1994)<br>Beck Anxiety Inventory (Beck et al., 1988).
		Negative Affect:<br>Measures that assess how much negative effect the individual	PANAS–Negative Affect (Watson et al., 1988).

		experiences in their life.	
		Body Dissatisfaction<br><br>Measures an individual's negative attitudes or feelings towards their own physical appearance.	Appearance evaluation subscale of the multidimensional body-self relations questionnaire (Cash, 2017).
Social Wellbeing	Positive Indicators	Social Connectedness<br><br>Measures perceived closeness and belonging in relationships and society.	The Social Connectedness scale (Lee & Robbins, 1995).
		Perceived Social Support<br><br>Measures the subjective feeling of having support from	Social Support Questionnaire (SSQ; Sarason et al., 1983).<br><br>The Child and Adolescent Social Support Scale.

		others. E.g. family, peers, and teachers.	(CASSS; Malecki et al., 2018).
	Negative Indicators	Loneliness<br>Measures subjective feelings of isolation and lack of social connection.	UCLA (Russell et al., 1978).
		Social Isolation<br>Measures lack of social networks and frequency of social interactions.	Lubben Social Network Scale (Lubben et al., 2006).
		Peer Rejection<br>Measures the degree to which an individual is disliked, excluded, or rejected by peers.	Social peer rejection measure (Lev-Wiesel et al., 2013).
		Cyberbullying Victimisation<br>Measures experiences of being targeted or harassed online.	Scale of Victimisation through the Cell Phone and Internet (Álvarez-García et al., 2017).

Tibbs M, *et al. BMJ Open* 2026; 16:e119555. doi: 10.1136/bmjopen-2026-119555

			Cyberbullying victimization (CBV) scale (Lee et al., 2017).
		Cyberbullying Perpetration<br>Measures engagement in online bullying behaviours, such as sending harmful messages, spreading rumours, or excluding others digitally.	Scale of Aggression through the Cell Phone and Internet (Álvarez-García et al., 2016)<br><br>Cyberbullying perpetration (CBP) scale (Lee et al., 2017)
Physical Wellbeing	Positive/Negative Indicators	Self-Reported Health Status<br>Measures perceived overall general health.	Self-rated health (SRH; Bombak, 2013).
		Sleep Quality<br>Measures satisfaction with sleep and restfulness.	Pittsburgh Sleep Quality Index (Buysse et al., 1989).
		Substance Use Behaviours	Risk Behaviour Index–5 items on tobacco, alcohol, and

		Measures risk behaviours related to tobacco, alcohol, and drug use.	drug use (Williams et al., 2000).
		Physical Activity<br>Measures frequency and intensity of exercise.	Physical Activity Questionnaire for Adolescents (PAQ-A; Kowalski et al., 1997).
	Negative Indicators	Perceived/Self-Reported Sleep Problems<br>Measures difficulties falling asleep, staying asleep, or feeling rested, based on self-report.	Adolescent Sleep Hygiene Scale (ASHS; LeBourgeois et al., 2005).
		Sedentary Behaviour<br>Measures time spent sitting or engaging in low-energy activities.	Sedentary Behavior Questionnaire (SBQ; Rosenberg et al., 2010).

Tibbs M, *et al. BMJ Open* 2026; 16:e119555. doi: 10.1136/bmjopen-2026-119555

	Positive	Health-Related Quality of Life<br>Measures the impact of various dimensions of health on quality of life.	SF-36 (Short Form 36 Health Survey; Ware & Sherbourne, 1992).
Digital-Specific Wellbeing	Negative	Social media addiction<br>Measures problematic or compulsive use of social media.	The Bergen Facebook Addiction Scale (BFAS) (Bowman & Clark-Gordon, 2019).
		Problematic social media use<br>Measures negative patterns of use which disrupt functioning.	Social Media Disorder scale (Boer et al., 2022).<br>Bergen Social Media Addiction Scale (BSMAS; Lin et al., 2017).
		Online gambling behaviour<br>Measures problem gambling and related	Adolescent Gambling Inventory's (CAGI) Problem Gambling Severity Index (PGSI; Tremblay et al., 2010).

Tibbs M, *et al. BMJ Open* 2026; 16:e119555. doi: 10.1136/bmjopen-2026-119555

		harms in online settings.	
		Online financial risk behaviours<br>Measures risky financial actions online and related consequences.	DOSPERT - Financial domain specific (Weber et al., 2002).
		Compulsive tech use<br>Measures excessive or uncontrolled use of digital devices or the internet leading to negative consequences/ functional impairment.	The Problematic and Risky Internet Use Screening Scale (PRIUSS; Jelenchick et al., 2014).

*Illustrative examples for the purposes of the protocol.

Tibbs M, *et al. BMJ Open* 2026; 16:e119555. doi: 10.1136/bmjopen-2026-119555

Supplement 3. *Categorisation of Media and Digital Literacy Outcomes for the Current Review**

Domain	Definition	Example of Measures
Functional Skills	The skills/practical ability to access and use devices and digital technologies.	● Internet skills for school (ISFS; Kuhlemeier & Hemker, 2007).<br>● iKnow measure (internet knowledge), (Potosky, 2007).<br>● yDSI skills, content creation & production dimension (Helsper et al., 2021).
Critical Skills	The ability to evaluate, assess and analyse media in a critical way.	● Media Literacy Scale (Arke & Primack, 2009).<br>● MeHLit – Media Health Literacy Scale (Nazarnia et al., 2022).<br>● Media Attitudes Questionnaire-Realism Scepticism (Irving et al., 1998).<br>● Critical Processing of Beauty Images Scale (CPBI; Engeln-Maddox & Miller, 2008).<br>● The Critical Thinking about Media Messages (CTMM) scale, (McLean et al., 2016).

Tibbs M, *et al. BMJ Open* 2026; 16:e119555. doi: 10.1136/bmjopen-2026-119555

Creative Production	The ability to create, develop and distribute various forms of media/content.	● yDSI skills, content creation & production dimension (Helsper et al., 2021).

*Illustrative examples for the purposes of the protocol.
