# Supplementary material for: Wellbeing-focused media literacy interventions in secondary schools: A systematic review and meta-analysis protocol
Source: BMJ Open. 2026 Jun 16;16(6):e119555. doi: 10.1136/bmjopen-2026-119555 (PMC13295985; doi:10.1136/bmjopen-2026-119555)
Supplement: Reviewer comments [file bmjopen-2026-119555.reviewer_comments.pdf]

PEER REVIEW HISTORY

BMJ Open publishes all reviews undertaken for accepted manuscripts. Reviewers are asked to complete a checklist review form and are provided with free text boxes to elaborate on their assessment. These free text comments are reproduced below.

ARTICLE DETAILS

Title (Provisional)

Wellbeing-focused Media Literacy Interventions in Secondary Schools: A Systematic Review and Meta-analysis Protocol

Authors

Tibbs, Maria; Francis, Gill; Mountain, Jennifer; Polizzi, Gianfranco; Bell, Beth T

VERSION 1 - REVIEW

Reviewer 1

Name Tang, Xiangdong

Affiliation Sichuan University West China Hospital, Sleep Medicine Center, Mental Health Center, National Center for Mental Disorders, Sleep Research Laboratory, Department of Respiratory and Clinical Care Medicine

Date 05-Apr-2026

COI None

Thank you for inviting me to review the manuscript entitled "Wellbeing-focused Media Literacy Interventions in Secondary Schools: A Systematic Review and Meta-analysis Protocol". Overall, this protocol is methodologically rigorous and has significant research value. However, the following issues need to be addressed:

1. The authors use "media literacy interventions" as a summary term, which may mask substantial heterogeneity across different types of interventions. It is recommended to conduct separate subgroup analyses for each wellbeing domain (psychological, social, physical, and digital) to examine whether effects differ meaningfully across outcome types. This approach will help determine which types of interventions work for which wellbeing outcomes.

2. There is a notable disparity in measurement tools across wellbeing domains. Psychological wellbeing has well-validated, standardised scales (e.g., SWLS, PANAS, WEMWBS), whereas digital wellbeing measures (e.g., social media addiction, problematic use) vary widely in structure, time frames, and cut-off values, lacking consensus. This measurement heterogeneity may compromise the comparability of effect sizes across domains.

3. The authors plan to include internal evaluation reports but do not specify the methodological quality standards they must meet. Clear inclusion criteria for grey literature are needed to ensure validity.

VERSION 1 - AUTHOR RESPONSE

Reviewer: 1

Comments to the Author:

Thank you for inviting me to review the manuscript entitled "Wellbeing-focused Media Literacy Interventions in Secondary Schools: A Systematic Review and Meta-analysis Protocol". Overall, this protocol is methodologically rigorous and has significant research value. However, the following issues need to be addressed:

1. The authors use "media literacy interventions" as a summary term, which may mask substantial heterogeneity across different types of interventions. It is recommended to conduct separate subgroup analyses for each wellbeing domain (psychological, social, physical, and digital) to examine whether effects differ meaningfully across outcome types. This approach will help determine which types of interventions work for which wellbeing outcomes.

Author Response: We thank the reviewer for this suggestion. We agree that considerable heterogeneity may exist across intervention types and wellbeing domains. As outlined in the protocol, outcome domain (psychological/subjective, social, physical, digital) will be included as a categorical moderator to formally test whether intervention effects differ across domains (page 23). We have also clarified that, where substantial or unforeseen heterogeneity is identified and sufficient comparable data are available, we may conduct separate three-level or standard meta-analyses within specific wellbeing domains to improve conceptual and methodological comparability of pooled estimates (page 24).

2. There is a notable disparity in measurement tools across wellbeing domains. Psychological wellbeing has well-validated, standardised scales (e.g., SWLS, PANAS, WEMWBS), whereas digital wellbeing measures (e.g., social media addiction, problematic use) vary widely in structure, time frames, and cut-off values, lacking consensus. This measurement heterogeneity may compromise the comparability of effect sizes across domains.

Author Response: We agree that measurement heterogeneity, particularly within digital wellbeing, is an important consideration. To address this, interpretation of pooled effects will be guided by conceptual alignment of outcomes, and outcome domains will be examined as a moderator to assess whether effects differ systematically across

domains. We have also clarified that where measures are conceptually or operationally heterogeneous and cannot be meaningfully compared, outcomes will be synthesised narratively rather than pooled. As outlined above, we have also clarified that we may conduct separate three-level or standard meta-analyses within specific wellbeing domains (see page 23 and page 24).

3. The authors plan to include internal evaluation reports but do not specify the methodological quality standards they must meet. Clear inclusion criteria for grey literature are needed to ensure validity.

Author Response: We thank the reviewer for highlighting the need for greater clarity regarding grey literature inclusion. We have now specified that all grey literature must meet the same inclusion criteria as peer-reviewed studies, such as the use of experimental or quasi-experimental designs with a comparator group and sufficient methodological detail to permit quality appraisal. All included studies, including grey literature, will be assessed using the same risk of bias procedures (see page 12 & 21).
