# Supplementary material for: Wellbeing-focused media literacy interventions in secondary schools: A systematic review and meta-analysis protocol
Source: BMJ Open. 2026 Jun 16;16(6):e119555. doi: 10.1136/bmjopen-2026-119555 (PMC13295985; doi:10.1136/bmjopen-2026-119555)
Supplement: Author's manuscript [file bmjopen-2026-119555.draft_revisions.pdf]

BMJ Open is committed to open peer review. As part of this commitment we make the peer review history of every article we publish publicly available.

When an article is published we post the peer reviewers' comments and the authors' responses online. We also post the versions of the paper that were used during peer review. These are the versions that the peer review comments apply to.

The versions of the paper that follow are the versions that were submitted during the peer review process. They are not the versions of record or the final published versions. They should not be cited or distributed as the published version of this manuscript.

BMJ Open is an open access journal and the full, final, typeset and author-corrected version of record of the manuscript is available on our site with no access controls, subscription charges or pay-per-view fees (http://bmjopen.bmj.com).

If you have any questions on BMJ Open's open peer review process please email info.bmjopen@bmj.com

BMJ Open

Wellbeing-focused Media Literacy Interventions in Secondary Schools: A Systematic Review and Meta-analysis Protocol

Journal:	*BMJ Open*
Manuscript ID	bmjopen-2026-119555
Article Type:	Protocol
Date Submitted by the Author:	12-Mar-2026
Complete List of Authors:	Tibbs, Maria; University of York, Psychology in Education Research Centre<br>Francis, Gill ; University of York, Psychology in Education Research Centre<br>Mountain, Jennifer ; University of York, Psychology in Education Research Centre<br>Polizzi, Gianfranco ; University of Birmingham, Department of Linguistics and Communication<br>Bell, Beth T; University of York, Psychology in Education Research Centre
Keywords:	Meta-Analysis, Systematic Review, MENTAL HEALTH, Schools, Social Media

SCHOLARONE™
MANUSCRIPTS

Wellbeing-focused Media Literacy Interventions in Secondary Schools: A Systematic Review and Meta-analysis Protocol

Maria Tibbs[1], Gill Francis[1], Jennifer Mountain[1], Gianfranco Polizzi[2], Beth T. Bell[1*].

[1] Psychology in Education Research Centre, Department of Education, University of York, United Kingdom.

[2] Department of Linguistics and Communication, University of Birmingham, Birmingham, United Kingdom.

***Corresponding author**

Email: Beth.bell@york.ac.uk.

Keywords: Adolescent wellbeing; school-based interventions; media literacy; digital literacy; digital wellbeing; meta-analysis; systematic review.

Wordcount: 4,265

Abstract

Background

Media and digital technologies shape adolescent development across interconnected online and offline contexts, offering opportunities for learning and connection while also exposing young people to risks that can affect wellbeing. Wellbeing-focused media literacy interventions aim to support young people in understanding and managing how media and technology shape their experiences and wellbeing. Schools are well placed to deliver such interventions at scale through curricular integration. However, existing interventions vary widely in focus, design, and evaluation quality, making it difficult to determine what works and why. This review synthesises school-based wellbeing-focused media literacy interventions and evaluates their effectiveness across wellbeing outcomes.

Methods

A range of interdisciplinary databases (ACM Digital Library, British Education Index, CINAHL, ERIC, MEDLINE, PsycINFO, Scopus, Web of Science) will be searched, alongside targeted grey literature searches of internal evaluation reports and dissertations. Eligible studies will include school-based interventions for adolescents aged 11 to 18. Abstracts and full texts will be screened in Covidence, and relevant data extracted using an adapted TIDieR framework. A three-level random effects meta-analysis will estimate pooled effects across psychological, social, physical, and digital wellbeing domains. Narrative synthesis, informed by the adapted TIDieR framework, will explore intervention content, delivery, and theoretical foundations to identify mechanisms of change and sources of heterogeneity.

Discussion

This review will provide the first comprehensive synthesis of wellbeing-focused media literacy interventions, clarifying which interventions are effective and identifying potential mechanisms of change. Findings will inform future research, policy and practice.

Ethics and dissemination

Ethical approval is not required for this systematic review, as it will only include published data. The review findings will be disseminated through a peer-reviewed publication and conference presentations.

Article Summary

- Uses a three level random effects meta-analysis that accounts for dependency among multiple effect sizes within studies, allowing more accurate synthesis of complex intervention outcomes.
- Comprehensive interdisciplinary search across psychology, education, public health and human computer interaction databases alongside grey literature sources to maximise coverage of relevant interventions.
- Integrates interventions targeting wellbeing outcomes associated with media and technology use including psychological, social, physical and digital wellbeing within a single media literacy framework, helping consolidate previously fragmented areas of research.
- Intervention characteristics will be systematically coded using an adapted TIDieR framework to identify intervention components and mechanisms of change.
- Substantial heterogeneity in intervention content, theoretical frameworks, delivery formats and outcome measures may still limit comparability across studies despite the use of a multilevel meta-analytic approach and use of subgroup analyses.

For peer review only - http://bmjopen.bmj.com/site/about/guidelines.xhtml

Introduction

Rates of mental health difficulties among adolescents have risen globally in recent decades (1–3), accompanied by declines in feelings of happiness, and positive affect (4–6). Most mental health difficulties emerge before the age of 25 (7), and in the United Kingdom one in five adolescents were reported to have a probable mental health disorder in 2023 (8). At the same time, there has been a rapid growth in youth digital technology use, with adolescents in the United Kingdom spending an average of four to six hours per day online (9). While these trends have unfolded concurrently, existing evidence suggests that technologies are not inherently harmful to young people, but rather associations between digital technology use and mental health are complex and shaped by a wide range of social, developmental, and contextual factors.

For contemporary adolescents, social life, learning, and self-expression are increasingly shaped by media and digital technologies, including social media platforms such as YouTube, TikTok, Snapchat, and WhatsApp (10–12). These technologies provide opportunities for connection, creativity, and community. However, they can also expose young people to cyberbullying, harmful or distressing content, reinforce sedentary behaviours, and promote upward comparison, all of which may undermine mental health and wellbeing (8,13–17). Supporting young people to develop capable and informed engagement with media and technology is therefore a pressing public health and educational concern (18).

Defining Wellbeing

Wellbeing is commonly defined as "*how people feel and how they function, both on a personal and a social level and how they evaluate their lives as a whole*" (19), though its conceptualisation and measurement vary considerably across the literature (20,21). It is widely recognised as a multidimensional construct, encompassing psychological, physical

For peer review only - http://bmjopen.bmj.com/site/about/guidelines.xhtml

and social domains (22). Psychological wellbeing includes two components; subjective (hedonic) wellbeing, such as life satisfaction and affect balance (23,24) and psychological (eudaimonic) wellbeing, which focuses on qualities associated with flourishing, such as purpose and personal growth (25,26). Physical wellbeing is frequently indexed through engagement in health-promoting behaviours (e.g., physical activity; (27) and avoidance of health-risk behaviours (e.g., smoking and substance use; (28). Social wellbeing captures the extent to which individuals feel integrated into and supported by their social networks and communities, encompassing experiences of belonging, acceptance, and positive relationships (29). Wellbeing, at both the personal and social level, can also be measured using more objective indicators, including physical/mental health status, educational attainment, socioeconomic security, and housing quality (30).

As young people's everyday lives have become deeply intertwined with digital technologies, scholars have increasingly argued that wellbeing frameworks must also incorporate a digital dimension. Digital wellbeing has been defined as the "*subjective individual experience of optimal balance between the benefits and drawbacks obtained from mobile connectivity*" (31). Promoting wellbeing in contemporary society therefore requires approaches that attend to multiple, interrelated dimensions of wellbeing and equip young people with the knowledge and skills to engage with media and technology in ways that support personal and social flourishing in an increasingly digital world.

Media Literacy Education

Media literacy education offers one potential pathway for promoting well-being among adolescents. Media literacy refers to the ability to access, analyse, evaluate, and create media, with an emphasis on critically understanding media messages and their underlying intentions (32,33). As media environments have become increasingly digitised, this framing has been

For peer review only - http://bmjopen.bmj.com/site/about/guidelines.xhtml

extended to include digital literacy, applying the same core functional, critical, and socioemotional competencies to online media, platforms, and technologies, alongside related literacies such as information and AI literacy (34,35). In this framing, digital literacy is typically understood as a context-specific subfield of media literacy, that encompasses the same underlying competencies. These include functional competencies such as searching for information, creating content, and navigating platforms (36) and critical competencies, such as evaluating online content and understanding how platform infrastructures and algorithms shape what users encounter and produce (37–39).

Accordingly, in this review we use the term *media literacy interventions* as an umbrella to refer to interventions grounded in media literacy frameworks and traditions, including those applied to digital contexts (e.g., AI literacy, social media literacy) and commonly described as digital literacy interventions. There is growing recognition that media literacy includes an integral wellbeing dimension, involving the socioemotional knowledge and skills needed to navigate the psychological demands of modern-day media and digital technology and to understand how these experiences can affect wellbeing (40). Emerging evidence suggests that these functional, critical and socioemotional skills are needed to help young people use technology in ways that maintain, enhance, support and protect wellbeing (41–44).

Wellbeing-focused Media Literacy Interventions

Schools have become a key setting for initiatives aimed at improving young people's media literacy in relation to wellbeing, as they reach learners during formative developmental periods, enable integration into curricula, and allow delivery at scale (45). In the United Kingdom, improving media and digital skills is now a governmental priority (46), and education on some online harms is mandated through the Relationships, Sex and Health Education curriculum (47).

For peer review only - http://bmjopen.bmj.com/site/about/guidelines.xhtml

Increasingly, a diverse range of interventions aim to foster media literacy in ways that support youth wellbeing in both online and offline contexts. For example, interventions have been developed to promote positive body image by fostering media literacy in relation to harmful appearance-related messaging across media and technology, while also teaching psychological and behavioural coping strategies (48,49). Interventions have also been developed to promote social wellbeing in online spaces by providing education related to how the design features and social affordances of online media environments may shape and sustain cyberbullying behaviours and social relationships (50,51). While these interventions vary in terms of content and approach, a commonality is that all aim to *develop the knowledge and skills needed to foster wellbeing in relation to media and technology* through three interrelated sets of competencies:

(1) Critical knowledge and skills to analyse and evaluate media and technology.

(2) Functional knowledge and skills to navigate and operate media and technology in autonomous ways.

(3) Socio-emotional knowledge and skills to manage personal and social wellbeing in relation to media and technology.

In many cases, wellbeing is positioned as an assumed or downstream outcome arising from the development and application of these competencies, rather than as an explicit focus of intervention theory or evaluation. We refer to this diverse group of approaches as *wellbeing-focused media literacy interventions*.

For peer review only - http://bmjopen.bmj.com/site/about/guidelines.xhtml

Despite growing recognition of their educational and public health relevance (52), little is known about which wellbeing-focused media literacy interventions work, for whom, and why. Existing reviews have largely examined intervention effectiveness within specific domains, such as cyberbullying (53) and body image (54), even though interventions often share elements that promote critical awareness, self-reflection, and behavioural change (55). This fragmentation prevents understanding of shared mechanisms or "active ingredients" that drive positive outcomes across intervention types and limits understanding of what approaches are most effective or transferable to real-world educational settings.

Objectives

Accordingly, this systematic review aims to identify classroom-based wellbeing-focused media literacy interventions that are effective among adolescents aged 11 to 18. Furthermore, it aims to examine the components and characteristics that underpin effectiveness. Specifically, it addresses the following questions:

1. Do school-based wellbeing-focused media literacy interventions improve youth wellbeing outcomes (psychological/subjective, social, physical, and digital)?

2. What active ingredients (components & characteristics) underpin effective school-based well-being focused media literacy interventions?

For peer review only - http://bmjopen.bmj.com/site/about/guidelines.xhtml

Materials and Method

This study will conduct a systematic review and meta-analysis examining the effectiveness of eligible interventions. The review will include experimental and quasi-experimental studies, including dissertations employing these designs, provided they meet the predefined eligibility criteria. Where sufficient comparable data are available, quantitative findings from eligible studies will be synthesised using meta-analysis to estimate the effects of the interventions under investigation.

Protocol and Registration

The current protocol is aligned with the Preferred Reporting Items for Systematic Reviews and Meta-Analyses Protocols (PRISMA-P; (56), see supplementary materials. The subsequent systematic review and meta-analysis will be reported following the Preferred Reporting Items for Systematic Reviews and Meta-Analyses (PRIMSA; (57).

This protocol has been preregistered on the Open Science Framework Platform (OSF; 10.17605/OSF.IO/APM7F) and PROSPERO (CRD420251269724). Any significant amendments made to the protocol will be documented and reported in the final systematic review manuscript. Database searches have been conducted, and title and abstract screening is currently ongoing, with completion anticipated by November 2026.

Information Sources and Search Strategy

The search will include both peer-reviewed and grey literature published in English, with no geographic restrictions. Peer-reviewed studies will be identified through interdisciplinary databases to ensure comprehensive coverage across education, psychology, public health and human computer interaction: ACM Digital Library, British Education Index, CINAHL, ERIC (Education Resources Information Centre), MEDLINE, PsycINFO, Scopus, Web of Science. Grey literature will include doctoral theses and publicly available internal reports evaluating

For peer review only - http://bmjopen.bmj.com/site/about/guidelines.xhtml

interventions with randomised designs, as these sources can provide valuable, otherwise unpublished evidence subjected to some level of independent scrutiny. Doctoral theses will be identified through ProQuest Dissertations and Theses Global. The websites of reputable media literacy and/or educational evaluation organisation websites (e.g., Education Endowment Foundation, Internet Matters, PSHE Association) will be searched for relevant internal evaluation reports. The rationale for including each database is presented in **Table 1**. To ensure all relevant articles will be captured, a process of forward reference and backward citation checking using *citationchaser* (58) will be conducted on eligible full texts.

The search strategy was developed in collaboration with a library specialist at the University of York and through examination of previous systematic reviews in the areas of digital literacy, media literacy, and wellbeing. Search terms focused on the core domains under investigation: the population of interest, characteristics of the intervention, relevant outcomes, the setting within which interventions are implemented, and study design, as well as MeSH terms or subject areas relevant to each database where applicable. Considering the breadth of potential wellbeing outcomes, the search strategy was conducted separately for each of the four wellbeing domains (psychological/subjective, social, physical, and digital) to ensure sufficient and relevant retrieval of studies. See **Supplementary Material 1** for an example SCOPUS search strategy.

Table 1.

Rationale for bibliographic databases

**Database**	**Rationale**

ERIC (Education Resources Information Centre)	Captures literature focused on education and learning contexts.
British Education Index	Covers educational literature focused within the British context, that may not be captured within ERIC.
Web of Science	Broad, multidisciplinary database indexing a wide range of high-impact peer-reviewed research.
PsycINFO	Specialises in psychology and related behavioural sciences.
CINAHL	Covers nursing, allied health, and applied healthcare research.
MEDLINE	Biomedical and public health database; includes extensive coverage of health interventions and uses MeSH terms, enhancing search precision.
SCOPUS	Indexes a broad range of journals across health sciences, technology, and education.
ACM Digital Library	Houses studies from human–computer interaction, a field that often contributes to research on digital literacy and technology use.

For peer review only - http://bmjopen.bmj.com/site/about/guidelines.xhtml

ProQuest Dissertations and Theses Global	Grey literature database that houses doctoral theses.

Eligibility Criteria

To be included in the current systematic review, studies will need to meet the pre-defined inclusion and exclusion criteria as informed by the Population, Intervention, Comparison, Outcome, Study Design (PICOS) model. For a summary of the inclusion criteria, **see Table 2.**

Population

To be included in this review, studies must focus on young people engaged in formal second level education aged 11- 18. This age range was selected to capture developmental stages typically corresponding to secondary-level schooling across international education systems, while excluding primary and post-secondary contexts.

Intervention

Eligible interventions must include a media literacy component, defined as educational content focused on developing adolescents' ability to access, analyse, evaluate, and create media, with an emphasis on critically understanding media messages and their underlying intentions (32,33). Media or technology must be an active target of the intervention, rather than solely the mode of delivery.

Interventions may contain one or more of the following media literacy components, which reflect different ways in which wellbeing-focused media literacy is operationalised in practice:

1. **Critical:** The knowledge and skills needed to critically analyse and evaluate media and technology through a wellbeing lens (e.g. understanding persuasive intent, platform design, algorithms, or how media features shape experiences relevant to wellbeing).
2. **Functional:** The knowledge and skills needed to navigate and operate media and technology in ways that may support wellbeing, including modifying engagement, using safety and support features, and enacting boundaries or help-seeking behaviours within digital environments.
3. **Socio-emotional:** The knowledge and skills needed to recognise and reflect on emotional and social responses to media and technology, and to evaluate when media engagement supports or undermines wellbeing.

Comparators

Studies will be included if they evaluate the intervention against either a passive control condition (e.g., no intervention, usual care, or waitlist/delayed care) or an active control condition (e.g., an alternative intervention or the same intervention delivered at a different time point). Studies that do not include a comparison or control group, such as pre–post, uncontrolled, or purely observational designs, will be excluded.

Outcomes

The primary outcome is wellbeing, which we define broadly as "*how people feel and how they function, both on a personal and a social level, and how they evaluate their lives as a whole*" (19). To structure our analysis, we draw on the World Health Organisation's three-domain model of wellbeing, which includes psychological, social, and physical dimensions (22). We extend this framework by adding a fourth domain: digital wellbeing. This expanded model allows us to capture the full spectrum of experiences and risks associated with young

people's digital lives. For the purposes of this review, we define these four domains as follows:

1. ***Psychological/Subjective wellbeing*** reflects individuals' evaluations and lived experiences of their own lives, including their sense of life satisfaction, experiences of positive and negative emotions, self-concept, the absence of mental health difficulties, and overall capacity to flourish (59,60).
2. ***Social wellbeing*** refers to the extent to which individuals feel integrated into and supported by their social networks and communities (29). This can include concepts such as belonging, friendship quality, as well as experiences of social isolation and loneliness.
3. ***Physical wellbeing*** can be described as the ability to maintain bodily health and energy through balanced behaviours such as exercise, diet, sleep, sexual health, and avoidance of harmful habits (61,62).
4. ***Digital wellbeing*** refers to individuals' ability to engage with digital technologies in ways that support wellbeing, including feeling safe, in control, and supported in online environments, as well as minimising exposure to digital risks such as harmful content, excessive use, or negative social interactions.

Secondary outcomes include indicators of digital, media, and information literacy, reflecting young people's knowledge, skills, and critical competencies in navigating online environments. For detail on the categorisation and representative measures, see **Supplementary Material 2 and 3.**

Study Design

This review will include English language, peer-reviewed experimental and quasi-experimental studies with a comparator or control group, including randomised controlled

trials (cluster and individual) and non-randomised comparison designs (e.g. matched or non-equivalent control groups). Eligible studies must provide a quantitative comparison between intervention and control conditions to assess effectiveness or efficacy.

Timeframe

The search period was set from 2000 to the present date to capture developments in digital literacy during a transitional period of conceptual overlap with traditional media literacy, when technologies were beginning to be integrated into media literacy education and regulation (63). These developments are exemplified by the enactment of international policies such as the U.S. Children's Internet Protection Act (2000), the UK Communications Act (2003), and the EU Action Plan for a Safer Internet (1999-2004), which increasingly framed media literacy around online safety, responsible use, and digital participation alongside traditional media.

For peer review only - http://bmjopen.bmj.com/site/about/guidelines.xhtml

Table 2. *Inclusion/Exclusion Criteria*

	**Inclusion**	**Exclusion**
Population	Young people aged 11-18 attending post-primary, secondary, or high school.	Young people not attending post-primary, secondary, or high school.<br><br>Young people aged <11 or >18.
Intervention	● School or classroom-based media literacy interventions that include education on the knowledge and skills needed to foster wellbeing in relation to media and technology including critical, functional and socio-emotional.	Interventions that:<br><br>● Are not conducted within education settings.<br>● Do not target students' knowledge and skills related to media and technology.
Comparator	Studies will be included if they compare the intervention with either a passive control (e.g., no intervention, usual care, or waitlist/delayed care) or active control (e.g., an	Studies without a comparison or control group (e.g., pre–post, uncontrolled, or observational designs) will be excluded.

	alternative intervention or the same intervention delivered at a different time point).	
Outcome	Studies will be included if their primary or secondary outcomes relate to either wellbeing and/or digital/media/information literacy.	● Studies will be excluded if they do not assess wellbeing as an outcome
Study Design	Studies will be included if they:<br>● Employ experimental designs with a comparator group, randomised controlled trials (including cluster-RCTs) and non-randomised comparison group designs (e.g. quasi-experiments using matched control or non-equivalent control group).	Studies will be excluded if they:<br>● Do not include a comparator group (e.g. single-group pre–post, purely observational, or cross-sectional studies).<br>● Employ qualitative designs.<br>● Are systematic reviews and meta-analyses.

For peer review only - http://bmjopen.bmj.com/site/about/guidelines.xhtml

	• Include a quantitative comparison between intervention and control or comparison conditions to evaluate effectiveness or efficacy.	• Are intervention protocols without published findings available at the time of synthesis.
Timeframe	2000 to present.	Studies published before the year 2000.

Data Management

Search results will be exported to Zotero reference managers and duplicates will be removed. Covidence systematic review software will be used to screen the abstracts and full texts of identified studies. Microsoft Excel will be used to extract relevant study data and to conduct quality appraisals of all included studies.

Selection of Studies and Data Extraction

Following the removal of duplicates, studies will be screened in two stages using Covidence. First, titles and abstracts will be assessed against the inclusion and exclusion criteria. Full texts of potentially relevant studies will then be retrieved and reviewed for eligibility. Four reviewers experienced in systematic review methodologies will contribute to the data screening and extraction process. At each stage studies will be double screened, and discrepancies will be resolved through a joint discussion by the two reviewers, and the opinion of a third reviewer will be obtained if agreement is not reached. Inter-rater reliability (e.g., Cohen's κ) will be calculated to assess agreement between the two reviewers at title and abstract screening as well as full-text screening.

Similarly, two reviewers will duplicate data extraction and coding to ensure accuracy and consistency. One reviewer will extract, and code all included studies, while the remaining three reviewers will each extract and code a proportion of the studies to enable cross-checking and verification. Extracted data will be compared for accuracy and completeness, with any discrepancies resolved through discussion and arbitration by a fourth author where necessary.

A pre-piloted data extraction form will be used to capture bibliographic details, sample characteristics, intervention and comparison conditions, and quantitative outcomes from each included study. The form will capture:

1. **Key study characteristics:** including author details, study date, country, study aims and objectives.

2. **Participant and School characteristics:** including sample size and composition (e.g., gender distribution, mean age, type of school [public/private], and contextual factors such as rural/urban setting of the school).

3. **Intervention characteristics:** We will record intervention characteristics using an adapted version of the Template for Intervention Description and Replication (TIDieR) framework (64). Using this, we will extract details on:

    a. The rationale, goal, and theoretical framing of the intervention.

    b. What is delivered (specific activities, and intervention components). This will be extended to capture which wellbeing-focused media literacy components are targeted. These components include: (1) critical knowledge and skills; (2) functional knowledge and skills; and (3) socio-emotional knowledge and skills. We acknowledge that these domains are interrelated and will not mutually exclusive. The coding framework will therefore allow for dual or multiple categorisations where components span domains.

    c. Who the intervention is delivered by (e.g., teachers, trained facilitators, peers).

    d. How the intervention was delivered (in-class online modules, group activities, presentations). This will be extended to extract details on mechanisms through which these interventions are theorised to achieve their outcomes. Specifically, we will code for teaching strategies and pedagogical approaches (e.g., reflection, discussion, experiential learning) and behaviour change techniques (BCT's; (65) used to enact behaviour change.

e. Where the intervention was delivered (e.g., within subject specific classes, after school, during pastoral care).

f. The frequency and duration of the intervention session(s).

g. Evidence of any modifications during the process of intervention implementation.

h. Details on adherence to any existing protocols.

i. Details of any adaptations or tailoring for different contexts.

4. **Outcomes:** including outcome domains, measures used, time points of assessment (e.g., pre-, post-, follow-up), directionality of wellbeing outcomes (positive or negative), and available relevant quantitative data (means, related effect sizes, and associated variance estimates) for meta-analytic calculations.

Quality Appraisal and Certainty of Evidence

The Cochrane Risk of Bias Tool (RoB2; (66) will be used to assess the risk of bias in randomised trials. This tool evaluates the risk of bias by assigning studies a quality rating of low, high, or unclear across several domains of bias. Studies deemed low quality will be retained for both the systematic review and meta-analysis. However, in line with recent meta-analyses of school-based interventions (67,68), sensitivity analyses will be conducted excluding low-quality studies to examine their influence on the magnitude and robustness of the overall effect. Funnel plots will be used to assess potential publication bias, and Egger's test will be employed to statistically evaluate the presence of small-study effects.

For peer review only - http://bmjopen.bmj.com/site/about/guidelines.xhtml

Data Synthesis

Narrative Synthesis

Extracted data will be tabulated and narratively synthesised following Popay et al's. (69) guidance on narrative synthesis. Firstly, study, participant, and intervention characteristics will be summarised descriptively using the adapted TIDieR framework. Second, interventions will be compared according to their wellbeing-focused media literacy components (knowledge/understanding and skills) and the mechanisms of change identified (teaching strategies, pedagogies, and behaviour change techniques). Finally, the synthesis will explore how variations in these components and mechanisms related to wellbeing outcomes across domains (psychological/subjective, social, physical, digital). Using this approach, the synthesis will provide an overview of patterns in intervention content (what) and the mechanisms to enact behaviour change (how), providing a working theory of how the intervention works to achieve its outcomes (70).

Meta-analysis

All studies meeting the inclusion criteria will be included in the systematic review; however, only studies reporting sufficient quantitative data on wellbeing outcomes (e.g., means and standard deviations, change scores, or effect estimates with variance) will be eligible for inclusion in the meta-analysis. Studies lacking extractable data or reporting outcomes that cannot be meaningfully standardised will be synthesised narratively. Where outcomes are reported on continuous scales, effect sizes are expected to be calculated as standardised mean differences (Hedges' g), which is commonly used in meta-analyses of school-based interventions to accommodate heterogeneous measures and small sample sizes. Effect sizes will be interpreted in relation to those reported in prior meta-analyses of school-based interventions, with emphasis on robustness and practical relevance rather than fixed magnitude thresholds.

We will perform a meta-analysis using an intention-to-treat approach, as opposed to a per-protocol approach, to minimise post-randomisation bias and overestimation of effects (71). This means that all participants originally allocated to intervention and control groups will be analysed according to their initial assignment, regardless of adherence or dropout, ensuring a more conservative and unbiased estimate of effect. To account for probable correlation and dependence between multiple effect sizes reported within individual studies, we will adopt a three-level random-effects meta-analysis to estimate pooled effects, a three-level random-effects meta-analysis partitions variance into three components:

1) Sampling variance around each observed effect size (sampling error within studies).
2) Variance between effect sizes within the same study
3) Variance between study effects.

In this analysis, effect sizes are nested within studies, and multiple outcomes reported by the same study are modelled as correlated at level two. This approach allows the inclusion of several non-independent outcomes per study while appropriately accounting for their clustering (72,73).

To assess what proportion of the observed variance reflects variance in true effects, the I^2 statistic will be calculated, with cut-offs of 25, 50, and 75% denoting low, moderate, and high levels of heterogeneity. 95% confidence intervals and for the I^2 values will be calculated using the method outlined by Borenstein (74). Finally, to explore sources of heterogeneity, we will conduct moderator analyses. Moderators will include study design characteristics (e.g., passive versus active control conditions, publication year), intervention characteristics (e.g., mode of delivery, number and duration of modules or lessons), and participant characteristics (e.g., gender composition). In addition, outcome domains (psychological/subjective, social, physical, digital) will be entered as categorical moderators alongside study-level characteristics such as intervention design and participant details. This

For peer review only - http://bmjopen.bmj.com/site/about/guidelines.xhtml

will allow us to formally test whether effects differ across outcome domains, as well as to examine potential sources of between-study heterogeneity. Following guidance from (75), analyses with categorical moderators will only be conducted if each category contains at least five cases.

As a robustness check, will fit a correlated-hierarchical effects model (CHE), which combines a hierarchical variance structure with robust variance estimation to permit inference without requiring known within-study correlations (76). This will be accompanied by further sensitivity analyses using examination of the potential impact of outliers and risk of bias. Importantly, meta-analysis decisions will be guided by theoretical coherence, measurement comparability, and the distribution of available data within each domain.

Statistical analyses will be conducted using the *metaSEM* package (77) in R statistical software (78).

Ethics and Dissemination

Ethical approval is not required for this systematic review, as it will only include published data. The review findings will be disseminated through a peer-reviewed publication and conference presentations.

Public and Patient Involvement (PPI)

The current protocol has been presented and reviewed by $N = 10$ key stakeholders including as representatives from charities and advocacy groups, education specialists, methodological experts, and academic researchers as well as $N = 2$ young people.

Deviations from the Protocol

Any deviations from the registered protocol will be updated and recorded on the OSF and Prospero websites.

For peer review only - http://bmjopen.bmj.com/site/about/guidelines.xhtml

Discussion

Existing systematic reviews have largely examined the effectiveness of interventions targeting specific online harms (53,79,80), yet few have integrated and consolidated these within a broader wellbeing framework (81). To date, no review has been conducted examining the effectiveness of wellbeing-focused media literacy interventions. To date, no meta-analysis has examined the effectiveness of such interventions. Furthermore, no work has been done to visualise the body of evidence or research gaps relevant to wellbeing-focused media literacy interventions.

The proposed review will address this gap by synthesising evidence across school-based interventions that build young people's understanding of how digital experiences affect wellbeing and the skills needed to support it. The three-level meta-analysis will provide pooled estimates across psychological/subjective, social, physical, and digital wellbeing outcomes, while the accompanying narrative synthesis will identify active components and contextual factors shaping effectiveness. Findings will clarify how wellbeing-focused media literacy can be defined, implemented, and evaluated in educational settings, advancing both theoretical and practical understanding of how schools can support youth wellbeing through media and digital literacy programmes.

Strengths and Limitations

This review has several methodological and conceptual strengths. The protocol is preregistered on the Open Science Framework and PROSPERO and follows PRISMA-P guidance, supporting reproducibility and methodological rigour. The search strategy spans multiple interdisciplinary databases across education, psychology, public health, and human–computer interaction, alongside grey literature sources, increasing the likelihood of identifying a comprehensive body of relevant evidence. Conceptually, the review integrates

interventions targeting wellbeing outcomes associated with media and technology use including psychological, social, physical, and digital wellbeing within a single media literacy framework. This approach helps consolidate previously fragmented areas of research. In addition, intervention characteristics will be systematically coded using an adapted Template for Intervention Description and Replication (TIDieR) framework, enabling the identification of active components and mechanisms that may underpin effective wellbeing-focused media literacy interventions.

However, some limitations should be acknowledged. Considerable heterogeneity is also anticipated in intervention content, theoretical foundations, delivery formats, and outcome measures across studies. Although this heterogeneity will be explicitly accounted for using a three-level random-effects meta-analytic model that accommodates dependency among multiple effect sizes within studies, variation in intervention design and measurement may still limit comparability and complicate interpretation of pooled estimates. Finally, the strength of the conclusions will depend on the methodological quality and reporting practices of the included primary studies.

Conflicts of Interest

The authors declare no conflicts of interest.

Acknowledgements

The authors would like to thank members of both the steering committee and the youth advisory board for their invaluable contributions to the drafting of this manuscript.

Funding

This project has been funded by the Nuffield Foundation (Grant Number: EDO /FR-000025035), but the views expressed are those of the authors and not necessarily the Foundation. Visit nuffieldfoundation.org

Author Contributions

- **MT:** Conceptualisation, Methodology, Formal analysis plan, Investigation, Writing – original draft.
- **GF:** Conceptualisation, Methodology, Validation, Writing – review & editing.
- **JM:** Investigation, Data curation, Writing – review & editing.
- **GP:** Conceptualisation, Writing – review & editing.
- **BB:** Conceptualisation, Funding acquisition, Supervision, Writing – review & editing.

References

1. Cybulski L, Ashcroft DM, Carr MJ, Garg S, Chew-Graham CA, Kapur N, et al. Temporal trends in annual incidence rates for psychiatric disorders and self-harm among children and adolescents in the UK, 2003–2018. BMC Psychiatry. 2021 May 3;21(1):229.
2. Fitzgerald A, Mahon C, Shevlin M, Dooley B, Reilly AO. Exploring changing trends in depression and anxiety among adolescents from 2012 to 2019: Insights from My World repeated cross-sectional surveys. Early Interv Psychiatry. 2025;19(1):e13562.
3. Mojtabai R, Olfson M. National Trends in Mental Health Care for US Adolescents. JAMA Psychiatry. 2020 Jul 1;77(7):703–14.
4. Blanchflower DG, Bryson A, Xu X. The declining mental health of the young and the global disappearance of the unhappiness hump shape in age. PLOS ONE. 2025 Aug 27;20(8):e0327858.
5. Dumuid D, Singh B, Brinsley J, Virgara R, Curtis RG, Brinkman S, et al. Trends in Well-Being Among Youth in Australia, 2017-2022. JAMA Netw Open. 2023 Aug 22;6(8):e2330098.

For peer review only - http://bmjopen.bmj.com/site/about/guidelines.xhtml

6. Marquez J, Long E. A Global Decline in Adolescents’ Subjective Well-Being: a Comparative Study Exploring Patterns of Change in the Life Satisfaction of 15-Year-Old Students in 46 Countries. Child Indic Res. 2021 Jun 1;14(3):1251–92.

7. Solmi M, Radua J, Olivola M, Croce E, Soardo L, Salazar de Pablo G, et al. Age at onset of mental disorders worldwide: large-scale meta-analysis of 192 epidemiological studies. Mol Psychiatry. 2022;27(1):281–95.

8. NHS Digital. Mental Health of Children and Young People in England, 2023 - wave 4 follow up to the 2017 survey [Internet]. 2023 [cited 2025 Oct 5]. Available from: https://digital.nhs.uk/data-and-information/publications/statistical/mental-health-of-children-and-young-people-in-england/2023-wave-4-follow-up

9. Ofcom. Online Nation Report [Internet]. 2023 Nov [cited 2025 Oct 5]. Available from: https://www.ofcom.org.uk/siteassets/resources/documents/research-and-data/online-research/online-nation/2024/online-nation-2024-report.pdf?v=386238

10. Charmaraman L, Hodes R, Richer AM. Young sexual minority adolescent experiences of self-expression and isolation on social media: cross-sectional survey study. JMIR Ment Health. 2021;8(9):e26207.

11. Goodyear VA, Armour KM. Young People’s health-related learning through social media: What do teachers need to know? Teach Teach Educ. 2021;102:103340.

12. Mittmann G, Woodcock K, Dörfler S, Krammer I, Pollak I, Schrank B. “TikTok Is My Life and Snapchat Is My Ventricle”: A Mixed-Methods Study on the Role of Online Communication Tools for Friendships in Early Adolescents. J Early Adolesc. 2022 Feb 1;42(2):172–203.

13. Al-Jbouri E, Volk AA, Spadafora N, Andrews NC. Friends, followers, peers, and posts: adolescents’ in-person and online friendship networks and social media use influences on friendship closeness via the importance of technology for social connection. Front Dev Psychol. 2024;2:1419756.

14. Caba-Machado V, Mcilroy D, Padilla-Adamuz FM. Night-time use of electronic devices, fear of missing out, sleep difficulties, anxiety, and well-being in UK and Spain: a cross-cultural comparison. Curr Psychol. 2024;43(24):21134–45.

15. Engberg E, Leppänen MH, Sarkkola C, Viljakainen H. Physical Activity Among Preadolescents Modifies the Long-Term Association Between Sedentary Time Spent Using Digital Media and the Increased Risk of Being Overweight. 2021 Jul 29 [cited 2025 Oct 6]; Available from: https://journals.humankinetics.com/view/journals/jpah/18/9/article-p1105.xml

16. Peppler K, Dahn M. Social Media and Creativity. In: The Routledge International Handbook of Children, Adolescents, and Media. 2nd ed. Routledge; 2022.

For peer review only - http://bmjopen.bmj.com/site/about/guidelines.xhtml

17. Zheng Q, Yang W, Li T, Liu Z, Zhang L. Examining the Pathways to Social Media FOMO: Mediation Effects of Passive Engagement and Upward Social Comparisons. Int J Human–Computer Interact. 2025;1–8.

18. Büchi M. Digital well-being theory and research. New Media Soc. 2024 Jan 1;26(1):172–89.

19. Michaelson J, Mahony S, Schifferes J. Measuring wellbeing: A guide for practitioners. New Econ Found Lond. 2012;

20. Dodge R, Daly A, Huyton J, Sanders L. The challenge of defining wellbeing. Intnl. J. Wellbeing 2, 222–235. 2012;

21. Hone L, Schofield G, Jarden A. Conceptualizations of wellbeing: Insights from a prototype analysis on New Zealand workers. N Z J Hum Resour Manag. 2015;15(2):97–118.

22. World Health Organization. Summary reports on proceedings minutes and final acts of the international health conference held in New York from 19 June to 22 July 1946. N Y WHO. 1948;35.

23. Diener E, Suh EM, Lucas RE, Smith HL. Subjective well-being: three decades of progress. Psychol Bull. 1999;125(2):276.

24. Diener E, Ryan K. Subjective well-being: A general overview. South Afr J Psychol. 2009;39(4):391–406.

25. Ryan RM, Deci EL. On Happiness and Human Potentials: A Review of Research on Hedonic and Eudaimonic Well-Being. Annu Rev Psychol. 2001 Feb 1;52(Volume 52, 2001):141–66.

26. Ryff CD, Singer BH. Know Thyself and Become What You Are: A Eudaimonic Approach to Psychological Well-Being. J Happiness Stud. 2008 Jan 1;9(1):13–39.

27. Hennessey A, MacQuarrie S, Petersen KJ. Exploring physical, subjective and psychological wellbeing profile membership in adolescents: a latent profile analysis. BMC Psychol. 2024;12(1):720.

28. Dittmar H, Bond R, Hurst M, Kasser T. The relationship between materialism and personal well-being: A meta-analysis. J Pers Soc Psychol. 2014;107(5):879–924.

29. Keyes CLM. Social well-being. Soc Psychol Q. 1998;121–40.

30. Organisation for Economic Co-operation and Development Secretary-General, OECD iLibrary. How’s life?: measuring well-being. In Paris; 2011.

31. Vanden Abeele MMP. Digital Wellbeing as a Dynamic Construct. Commun Theory. 2021 Nov 1;31(4):932–55.

For peer review only - http://bmjopen.bmj.com/site/about/guidelines.xhtml

32. Aufderheide P. Media Literacy: From a Report of the National Leadership Conference on Media Literacy. In: Media Literacy Around the World. Routledge; 1997.

33. Hobbs R. Digital and Media Literacy: A Plan of Action. A White Paper on the Digital and Media Literacy Recommendations of the Knight Commission on the Information Needs of Communities in a Democracy [Internet]. Aspen Institute; 2010 [cited 2025 Oct 9]. Available from: https://eric.ed.gov/?id=ED523244

34. Leaning M. An approach to digital literacy through the integration of media and information literacy. Media Commun. 2019;7(2):4–13.

35. Wuyckens G, Landry N, Fastrez P. Untangling media literacy, information literacy, and digital literacy: A systematic meta-review of core concepts in media education. J Media Lit Educ. 2022 May 9;14(1):168–82.

36. Van Deursen AJ, Helsper EJ, Eynon R. Development and validation of the Internet Skills Scale (ISS). Inf Commun Soc. 2016;19(6):804–23.

37. Buckingham D. Digital Media Literacies: rethinking media education in the age of the Internet. Res Comp Int Educ. 2007;2(1):43–55.

38. Dezuanni M. Minecraft and children's digital making: implications for media literacy education. Learn Media Technol. 2018;43(3):236–49.

39. Polizzi G. Internet users' utopian/dystopian imaginaries of society in the digital age: Theorizing critical digital literacy and civic engagement. New Media Soc. 2023;25(6):1205–26.

40. Tuamsuk K, Subramaniam M. The current state and influential factors in the development of digital literacy in Thailand's higher education. Inf Learn Sci. 2017 May 8;118(5–6):235–51.

41. Gordon CS, Ferber K, Notley T, Rodgers RF, Bradshaw E, Basarkod G, et al. The relationship between media literacy and well-being: A systematic review and meta-analysis. Educ Res Rev. 2025 Nov 1;49:100731.

42. Kalmus V, Tambaum T, Abuladze L. Use of digital tools, digital skills and mental well-being. In 2023. p. 235–50.

43. Livingstone S, Mascheroni G, Stoilova M. The outcomes of gaining digital skills for young people's lives and wellbeing: A systematic evidence review. New Media Soc. 2023;25(5):1176–202.

44. Vissenberg J, De Coninck D, Mascheroni G, Joris W, d'Haenens L. Digital Skills and Digital Knowledge as Buffers Against Online Mis/Disinformation? Findings from a Survey Study Among Young People in Europe. Soc Media Soc. 2023 Oct 1;9(4):20563051231207859.

For peer review only - http://bmjopen.bmj.com/site/about/guidelines.xhtml

45. Carta MG, Di Fiandra T, Rampazzo L, Contu P, Preti A. An overview of international literature on school interventions to promote mental health and well-being in children and adolescents. Clin Pract Epidemiol Ment Health CP EMH. 2015;11(Suppl 1 M1):16.

46. DCMS. Department for Digital, Culture, Media and Sport: Annual Report and Accounts. [Internet]. 2022. Available from: https://assets.publishing.service.gov.uk/media/638883e6d3bf7f3282be4061/DCMS_Annual_Report_and_Accounts_2021-22_-_web_accessible_version.pdf

47. Department for Education. Teaching Online Safety in Schools [Internet]. 2023. Available from: https://www.gov.uk/government/publications/teaching-online-safety-in-schools/teaching-online-safety-in-schools

48. Bell BT, Taylor C, Paddock D, Bates A. Digital Bodies: A controlled evaluation of a brief classroom-based intervention for reducing negative body image among adolescents in the digital age. Br J Educ Psychol. 2022;92(1):280–98.

49. Gordon CS, Jarman HK, Rodgers RF, McLean SA, Slater A, Fuller-Tyszkiewicz M, et al. Outcomes of a cluster randomized controlled trial of the SoMe social media literacy program for improving body image-related outcomes in adolescent boys and girls. Nutrients. 2021;13(11):3825.

50. Kutok ER, Dunsiger S, Patena JV, Nugent NR, Riese A, Rosen RK, et al. A Cyberbullying Media-Based Prevention Intervention for Adolescents on Instagram: Pilot Randomized Controlled Trial. JMIR Ment Health. 2021 Sep 15;8(9):e26029.

51. Schoeps K, Villanueva L, Prado-Gascó VJ, Montoya-Castilla I. Development of Emotional Skills in Adolescents to Prevent Cyberbullying and Improve Subjective Well-Being. Front Psychol [Internet]. 2018 Oct 26 [cited 2025 Oct 8];9. Available from: https://www.frontiersin.org/journals/psychology/articles/10.3389/fpsyg.2018.02050/full

52. Ofcom. A Positive Vision for Media Literacy: Ofcom’s Three-Year Media Literacy Strategy [Internet]. 2024. Available from: https://www.ofcom.org.uk/siteassets/resources/documents/research-and-data/media-literacy-research/making-sense-of-media/media-literacy/ofcoms-three-year-media-literacy-strategy-final.pdf?v=392801

53. Ng ED, Chua JYX, Shorey S. The effectiveness of educational interventions on traditional bullying and cyberbullying among adolescents: A systematic review and meta-analysis. Trauma Violence Abuse. 2022;23(1):132–51.

54. Zuair AA, Sopory P. Effects of media health literacy school-based interventions on adolescents’ body image concerns, eating concerns, and thin-internalization attitudes: A systematic review and meta-analysis. Health Commun. 2022;37(1):20–8.

55. Weinstein E, James C. School-based initiatives promoting digital citizenship and healthy digital media use. Handb Adolesc Digit Media Use Ment Health. 2022;365.

For peer review only - http://bmjopen.bmj.com/site/about/guidelines.xhtml

56. Moher D, Shamseer L, Clarke M, Ghersi D, Liberati A, Petticrew M, et al. Preferred reporting items for systematic review and meta-analysis protocols (PRISMA-P) 2015 statement. Syst Rev. 2015 Jan 1;4(1):1.

57. Page MJ, McKenzie JE, Bossuyt PM, Boutron I, Hoffmann TC, Mulrow CD, et al. The PRISMA 2020 statement: an updated guideline for reporting systematic reviews. BMJ. 2021 Mar 29;372:n71.

58. Haddaway NR, Grainger MJ, Gray CT. Citationchaser: A tool for transparent and efficient forward and backward citation chasing in systematic searching. Res Synth Methods. 2022;13(4):533–45.

59. Adler A, Unanue W, Osin E, Ricard M, Alkire S, Seligman M. Psychological wellbeing. Happiness. 2017;118.

60. Stone AA, Mackie C, Framework P on MSWB in a PR, Statistics C on N, andEducation D on B and SS, Council NR. Introduction. In: Subjective Well-Being: Measuring Happiness, Suffering, and Other Dimensions of Experience [Internet] [Internet]. National Academies Press (US); 2013 [cited 2025 Oct 23]. Available from: https://www.ncbi.nlm.nih.gov/books/NBK179225/

61. Harvard University Health Sciences. Physical – Center for Wellness and Health Promotion [Internet]. n/d [cited 2025 Oct 23]. Available from: https://wellness.huhs.harvard.edu/your-wellbeing/wellbeing-framework/physical/

62. Hung ST, Cheng YC, Wu CC, Su CH. Examining Physical Wellness as the fundamental element for achieving holistic well-being in older persons: review of literature and practical application in Daily Life. J Multidiscip Healthc. 2023;1889–904.

63. Wallis R, Buckingham D. Media literacy: the UK's undead cultural policy. Int J Cult Policy. 2019 Feb 23;25(2):188–203.

64. Hoffmann TC, Glasziou PP, Boutron I, Milne R, Perera R, Moher D, et al. Better reporting of interventions: template for intervention description and replication (TIDieR) checklist and guide. BMJ. 2014 Mar 7;348:g1687.

65. Michie S, Richardson M, Johnston M, Abraham C, Francis J, Hardeman W, et al. The behavior change technique taxonomy (v1) of 93 hierarchically clustered techniques: building an international consensus for the reporting of behavior change interventions. Ann Behav Med. 2013;46(1):81–95.

66. Higgins J. Cochrane handbook for systematic reviews of interventions. Version 5.1. 0 [updated March 2011]. The Cochrane Collaboration. Www Cochrane-Handb Org. 2011;

67. Hodder RK, O'Brien KM, Lorien S, Wolfenden L, Moore THM, Hall A, et al. Interventions to prevent obesity in school-aged children 6-18 years: An update of a Cochrane systematic review and meta-analysis including studies from 2015–2021. eClinicalMedicine [Internet]. 2022 Dec 1 [cited 2026 Jan 8];54. Available from: https://www.thelancet.com/journals/eclinm/article/PIIS2589-5370(22)00365-0/fulltext

For peer review only - http://bmjopen.bmj.com/site/about/guidelines.xhtml

68. Zhang Q, Wang J, Neitzel A. School-based Mental Health Interventions Targeting Depression or Anxiety: A Meta-analysis of Rigorous Randomized Controlled Trials for School-aged Children and Adolescents. J Youth Adolesc. 2023 Jan 1;52(1):195–217.

69. Popay J, Roberts H, Sowden A, Petticrew M, Arai L, Rodgers M, et al. Guidance on the conduct of narrative synthesis in systematic reviews. Prod ESRC Methods Programme Version. 2006;1(1):b92.

70. Rodgers M, Sowden A, Petticrew M, Arai L, Roberts H, Britten N, et al. Testing methodological guidance on the conduct of narrative synthesis in systematic reviews: effectiveness of interventions to promote smoke alarm ownership and function. Evaluation. 2009;15(1):49–73.

71. McCoy CE. Understanding the Intention-to-treat Principle in Randomized Controlled Trials. West J Emerg Med. 2017 Oct;18(6):1075–8.

72. Cheung MWL. Modeling dependent effect sizes with three-level meta-analyses: A structural equation modeling approach. Psychol Methods. 2014;19(2):211–29.

73. Van den Noortgate W, López-López JA, Marín-Martínez F, Sánchez-Meca J. Three-level meta-analysis of dependent effect sizes. Behav Res Methods. 2013 Jun 1;45(2):576–94.

74. Borenstein M, Cooper H, Hedges L, Valentine J. Effect sizes for continuous data. Handb Res Synth Meta-Anal. 2009;2:221–35.

75. Weisz JR, Kuppens S, Ng MY, Eckshtain D, Ugueto AM, Vaughn-Coaxum R, et al. What five decades of research tells us about the effects of youth psychological therapy: A multilevel meta-analysis and implications for science and practice. Am Psychol. 2017;72(2):79–117.

76. Pustejovsky JE, Tipton E. Meta-analysis with Robust Variance Estimation: Expanding the Range of Working Models. Prev Sci Off J Soc Prev Res. 2022 Apr;23(3):425–38.

77. Cheung MWL. metaSEM: An R package for meta-analysis using structural equation modeling. Front Psychol. 2015;5:1521.

78. Core Team R. R: A language and environment for statistical computing [Software]. R Found Stat Comput. 2021;

79. Guest E, Zucchelli F, Costa B, Bhatia R, Halliwell E, Harcourt D. A systematic review of interventions aiming to promote positive body image in children and adolescents. Body Image. 2022 Sep 1;42:58–74.

80. Malinauskas R, Malinauskiene V. A meta-analysis of psychological interventions for Internet/smartphone addiction among adolescents. J Behav Addict. 2019;8(4):613–24.

81. Cowling M, Sim KN, Orlando J, Hamra J. Untangling Digital Safety, literacy, and Wellbeing in School activities for 10 to 13 Year Old Students. Educ Inf Technol. 2025;30(1):941–58.

For peer review only - http://bmjopen.bmj.com/site/about/guidelines.xhtml

Supplementary Material

Supplement 1. *Example SCOPUS Search String.*

**Wellbeing Domain**	**Search Strategy**
Psychological/Subjective Wellbeing:	TITLE-ABS-KEY ((adolescen* OR teen* OR student* OR pupil* OR youth OR "young pe*") AND (“digital skill*" OR "digital safety" OR "online safety" OR "online resilience" OR "digital resilience" OR "digital competenc*” OR "internet safety" OR "social media literacy" OR "digital literacy" OR "media literacy" OR "information literacy” OR "AI literacy” OR misinformation OR "social media" OR "digital media" OR "social network*" OR online) AND ("mental health" OR "mental illness" OR "mental disorder*" OR "psychological distress" OR wellbeing* OR "mental wellbeing" OR "life satisfaction" OR affect* OR "emotional wellbeing" OR "psychological wellbeing" OR resilience OR happiness OR optimism OR "personal growth" OR "self expression" OR identity OR suicid* OR "self-

For peer review only

	harm" OR depress* OR anxi* OR stress OR internali?* OR externali?* OR "self esteem" OR "self concept" OR "self worth" OR "body image" OR "eating disorder*" OR "disordered eating")<br><br>AND<br><br>("secondary school" OR "high school" OR "middle school" OR "post primary" OR "secondary education" OR classroom* OR "school-based" OR "whole school")<br><br>AND<br><br>(experiment* OR randomi?ed OR "randomi?ed controlled trial*" OR "randomi?ed control trial*" OR rct OR "cluster rct" OR "cluster randomi?ed*" OR "quasi-experiment*" OR evaluation OR pretest OR posttest OR "pre test" OR "post test" OR lesson* OR intervention* OR effectiveness)
Social Wellbeing	TITLE-ABS-KEY ((adolescen* OR teen* OR student* OR pupil* OR youth OR "young pe*”)<br><br>AND

For peer review only - http://bmjopen.bmj.com/site/about/guidelines.xhtml

	(“digital skill*" OR "digital safety" OR "online safety" OR "online resilience" OR "digital resilience" OR "digital competenc*” OR "internet safety" OR "social media literacy" OR "digital literacy" OR "media literacy" OR "information literacy” OR "AI literacy” OR misinformation OR "social media" OR "digital media" OR "social network*" OR online)<br><br>AND<br><br>(loneliness OR "social isolation" OR "social connect*" OR belonging OR friendship OR "social support" OR "peer rejection”)<br><br>AND

For peer review only - http://bmjopen.bmj.com/site/about/guidelines.xhtml

	("secondary school" OR "high school" OR "middle school" OR "post primary" OR "secondary education" OR classroom* OR "school-based" OR "whole school")<br><br>AND<br><br>(experiment* OR randomi?ed OR "randomi?ed controlled trial*" OR "randomi?ed control trial*" OR rct OR "cluster rct" OR "cluster randomi?ed*" OR "quasi-experiment*" OR evaluation OR pretest OR posttest OR "pre test" OR "post test" OR lesson* OR intervention* OR effectiveness)
Physical Wellbeing	TITLE-ABS-KEY ((adolescen* OR teen* OR student* OR pupil* OR youth OR "young pe*”)<br><br>AND

For peer review only - http://bmjopen.bmj.com/site/about/guidelines.xhtml

	(“digital skill*" OR "digital safety" OR "online safety" OR "online resilience" OR "digital resilience" OR "digital competenc*” OR "internet safety" OR "social media literacy" OR "digital literacy" OR "media literacy" OR "information literacy” OR "AI literacy” OR misinformation OR "social media" OR "digital media" OR "social network*" OR online)<br><br>AND<br><br>(“physical wellbeing" OR "physical well-being" OR sleep* OR "sedentary behav*" OR exercise OR "substance abuse" OR "substance use" OR "physical activ*" OR "health behav*" OR "sexual health")<br><br>AND<br><br>("secondary school" OR "high school" OR "middle school" OR "post primary" OR "secondary education" OR classroom* OR "school-based" OR "whole school")

For peer review only - http://bmjopen.bmj.com/site/about/guidelines.xhtml

	AND<br><br>(experiment* OR randomi?ed OR "randomi?ed controlled trial*" OR "randomi?ed control trial*" OR rct OR "cluster rct" OR "cluster randomi?ed*" OR "quasi-experiment*" OR evaluation OR pretest OR posttest OR "pre test" OR "post test" OR lesson* OR intervention* OR effectiveness)
Digital Wellbeing	TITLE-ABS-KEY ((adolescen* OR teen* OR student* OR pupil* OR youth OR "young pe*") <br>AND<br><br>(“digital skill*" OR "digital safety" OR "online safety" OR "online resilience" OR "digital resilience" OR "digital competenc*” OR "internet safety" OR "social media literacy" OR "digital literacy" OR "media literacy"

For peer review only - http://bmjopen.bmj.com/site/about/guidelines.xhtml

	OR "information literacy" OR "AI literacy" OR misinformation OR "social media" OR "digital media" OR "social network*" OR online)<br><br>AND<br><br>("digital well*" OR "online well*" OR cyberbull* OR bully* OR victimis* OR victimiz* OR harass* OR aggress* OR "online abuse" OR sext* OR groom* OR "harmful content" OR "violent content" OR "self-harm content" OR "hate speech" OR extremis* OR radicalis* OR radicaliz* OR "problematic internet*" OR "problematic social media*" OR "internet addiction" OR "social media addiction" OR "problematic smartphone*" OR "technology overuse" OR "gaming addiction" OR "problematic gaming" OR "online gambling" OR "screen time" OR "digital fatigue" OR "information overload" OR "fear of missing out" OR fomo OR pornograph* OR porn*)

For peer review only - http://bmjopen.bmj.com/site/about/guidelines.xhtml

	AND<br><br>("secondary school" OR "high school" OR "middle school" OR "post primary" OR "secondary education" OR classroom* OR "school-based" OR "whole school")<br><br>AND<br><br>(experiment* OR randomi?ed OR "randomi?ed controlled trial*" OR "randomi?ed control trial*" OR rct OR "cluster rct" OR "cluster randomi?ed*" OR "quasi-experiment*" OR evaluation OR pretest OR posttest OR "pre test" OR "post test" OR lesson* OR intervention* OR effectiveness)

For peer review only

4 **Supplement 2.** *Categorisation of Wellbeing Constructs for the Current Review**

5

**Wellbeing Domain**	**Direction of Indicator**	**Example & Definition**	**Example Representative Measures**
Psychological and Subjective Wellbeing	Positive Indicators	Life Satisfaction: Measures that assess satisfaction with life overall.	Satisfaction with Life Scale (Diener et al., 1985).
		Positive Affect: Measures that assess how much positive affect the individual experiences in their life.	PANAS–Positive Affect (Watson et al., 1988).
		Composite Wellbeing Measures which assess an individual's overall subjective wellbeing	Warwick Edinburgh Mental Wellbeing Scale (Tennant et al., 2007).

For peer review only - http://bmjopen.bmj.com/site/about/guidelines.xhtml

		Resilience<br><br>Measures which assess the ability to adapt and/or recover.	Adolescent Psychological Resilience Scale (APRS; Bulut et al., 2013).
		Optimism<br><br>Measures general expectations about positive outcomes.	Revised Life Orientation Test (LOT-R; Scheier et al., 1994).
		Quality of Life<br><br>Assesses overall satisfaction and fulfilment across life domains.	The Quality-of-Life Scale (QOLS; Burckhardt et al., 2003).
		Body Satisfaction<br><br>Measures contentment with various aspects of their body.	The Body Satisfaction Scale (Slade et al., 1990).
		Body Esteem<br><br>Measures positive self-evaluations of	The Body Esteem Scale (Franzoi & Shields, 1984).

For peer review only

For peer review only - http://bmjopen.bmj.com/site/about/guidelines.xhtml

		one's body and appearance.	
		Self Esteem<br><br>Measures an individual's overall sense of self-worth and value.	Rosenberg self-esteem scale (Rosenberg, 2011).
	Negative Indicators	DSM-V defined mental health outcomes (e.g., depression, anxiety, stress)<br><br>Measures which assess mental health outcomes as defined by the DSM-V.	Beck Depression Inventory (Beck, 1961).<br><br>State–Trait Anxiety Inventory (Spielberger, 1994)<br><br>Beck Anxiety Inventory (Beck et al., 1988).
		Negative Affect:<br><br>Measures that assess how much negative effect the individual	PANAS–Negative Affect (Watson et al., 1988).

For peer review only

		experiences in their life.	
		Body Dissatisfaction<br><br>Measures an individual's negative attitudes or feelings towards their own physical appearance.	Appearance evaluation subscale of the multidimensional body-self relations questionnaire (Cash, 2017).
Social Wellbeing	Positive Indicators	Social Connectedness<br><br>Measures perceived closeness and belonging in relationships and society.	The Social Connectedness scale (Lee & Robbins, 1995).
		Perceived Social Support<br><br>Measures the subjective feeling of having support from	Social Support Questionnaire (SSQ; Sarason et al., 1983).<br><br>The Child and Adolescent Social Support Scale.

For peer review only - http://bmjopen.bmj.com/site/about/guidelines.xhtml

		others. E.g. family, peers, and teachers.	(CASSS; Malecki et al., 2018).
	Negative Indicators	Loneliness<br><br>Measures subjective feelings of isolation and lack of social connection.	UCLA (Russell et al., 1978).
		Social Isolation<br><br>Measures lack of social networks and frequency of social interactions.	Lubben Social Network Scale (Lubben et al., 2006).
		Peer Rejection<br><br>Measures the degree to which an individual is disliked, excluded, or rejected by peers.	Social peer rejection measure (Lev-Wiesel et al., 2013).
		Cyberbullying Victimisation<br><br>Measures experiences of being targeted or harassed online.	Scale of Victimisation through the Cell Phone and Internet (Álvarez-García et al., 2017).

For peer review only - http://bmjopen.bmj.com/site/about/guidelines.xhtml

			Cyberbullying victimization (CBV) scale (Lee et al., 2017).
		Cyberbullying Perpetration<br><br>Measures engagement in online bullying behaviours, such as sending harmful messages, spreading rumours, or excluding others digitally.	Scale of Aggression through the Cell Phone and Internet (Álvarez-García et al., 2016)<br><br>Cyberbullying perpetration (CBP) scale (Lee et al., 2017)
Physical Wellbeing	Positive/Negative Indicators	Self-Reported Health Status<br><br>Measures perceived overall general health.	Self-rated health (SRH; Bombak, 2013).
		Sleep Quality<br><br>Measures satisfaction with sleep and restfulness.	Pittsburgh Sleep Quality Index (Buysse et al., 1989).
		Substance Use Behaviours	Risk Behaviour Index–5 items on tobacco, alcohol, and

For peer review only

		Measures risk behaviours related to tobacco, alcohol, and drug use.	drug use (Williams et al., 2000).
		Physical Activity<br><br>Measures frequency and intensity of exercise.	Physical Activity Questionnaire for Adolescents (PAQ-A; Kowalski et al., 1997).
	Negative Indicators	Perceived/Self-Reported Sleep Problems<br><br>Measures difficulties falling asleep, staying asleep, or feeling rested, based on self-report.	Adolescent Sleep Hygiene Scale (ASHS; LeBourgeois et al., 2005).
		Sedentary Behaviour<br><br>Measures time spent sitting or engaging in low-energy activities.	Sedentary Behavior Questionnaire (SBQ; Rosenberg et al., 2010).

For peer review only - http://bmjopen.bmj.com/site/about/guidelines.xhtml

	Positive	Health-Related Quality of Life<br><br>Measures the impact of various dimensions of health on quality of life.	SF-36 (Short Form 36 Health Survey; Ware & Sherbourne, 1992).
Digital-Specific Wellbeing	Negative	Social media addiction<br><br>Measures problematic or compulsive use of social media.	The Bergen Facebook Addiction Scale (BFAS) (Bowman & Clark-Gordon, 2019).
		Problematic social media use<br><br>Measures negative patterns of use which disrupt functioning.	Social Media Disorder scale (Boer et al., 2022).<br><br>Bergen Social Media Addiction Scale (BSMAS; Lin et al., 2017).
		Online gambling behaviour<br><br>Measures problem gambling and related	Adolescent Gambling Inventory's (CAGI) Problem Gambling Severity Index (PGSI; Tremblay et al., 2010).

For peer review only - http://bmjopen.bmj.com/site/about/guidelines.xhtml

		harms in online settings.	
		Online financial risk behaviours<br><br>Measures risky financial actions online and related consequences.	DOSPERT - Financial domain specific (Weber et al., 2002).
		Compulsive tech use<br><br>Measures excessive or uncontrolled use of digital devices or the internet leading to negative consequences/ functional impairment.	The Problematic and Risky Internet Use Screening Scale (PRIUSS; Jelenchick et al., 2014).

6 *Illustrative examples for the purposes of the protocol.

For peer review only

Supplement 3. *Categorisation of Media and Digital Literacy Outcomes for the Current Review**

Domain	Definition	Example of Measures
Functional Skills	The skills/practical ability to access and use devices and digital technologies.	• Internet skills for school (ISFS; Kuhlemeier & Hemker, 2007).<br>• iKnow measure (internet knowledge), (Potosky, 2007).<br>• yDSI skills, content creation & production dimension (Helsper et al., 2021).
Critical Skills	The ability to evaluate, assess and analyse media in a critical way.	• Media Literacy Scale (Arke & Primack, 2009).<br>• MeHLit – Media Health Literacy Scale (Nazarnia et al., 2022).<br>• Media Attitudes Questionnaire-Realism Scepticism (Irving et al., 1998).<br>• Critical Processing of Beauty Images Scale (CPBI; Engeln-Maddox & Miller, 2008).<br>• The Critical Thinking about Media Messages (CTMM) scale, (McLean et al., 2016).

For peer review only - http://bmjopen.bmj.com/site/about/guidelines.xhtml

Creative Production	The ability to create, develop and distribute various forms of media/content.	• yDSI skills, content creation & production dimension (Helsper et al., 2021).

*Illustrative examples for the purposes of the protocol.

For peer review only

For peer review only - http://bmjopen.bmj.com/site/about/guidelines.xhtml

PRISMA-P (Preferred Reporting Items for Systematic review and Meta-Analysis Protocols) 2015 checklist: recommended items to address in a systematic review protocol*

Section and topic	Item No	Checklist item	Location where item is reported
**ADMINISTRATIVE INFORMATION**			
Title:			
Identification	1a	Identify the report as a protocol of a systematic review	Pg. 1
Update	1b	If the protocol is for an update of a previous systematic review, identify as such	n/a
Registration	2	If registered, provide the name of the registry (such as PROSPERO) and registration number	Pg. 9
Authors:			
Contact	3a	Provide name, institutional affiliation, e-mail address of all protocol authors; provide physical mailing address of corresponding author	Pg. 1
Contributions	3b	Describe contributions of protocol authors and identify the guarantor of the review	Pg.1
Amendments	4	If the protocol represents an amendment of a previously completed or published protocol, identify as such and list changes; otherwise, state plan for documenting important protocol amendments	n/a
Support:			
Sources	5a	Indicate sources of financial or other support for the review	Pg. 25
Sponsor	5b	Provide name for the review funder and/or sponsor	Pg. 25
Role of sponsor or funder	5c	Describe roles of funder(s), sponsor(s), and/or institution(s), if any, in developing the protocol	Pg. 25
**INTRODUCTION**			
Rationale	6	Describe the rationale for the review in the context of what is already known	Pg. 4 - 8
Objectives	7	Provide an explicit statement of the question(s) the review will address with reference to participants, interventions, comparators, and outcomes (PICO)	Pg. 8
**METHODS**			
Eligibility criteria	8	Specify the study characteristics (such as PICO, study design, setting, time frame) and report characteristics (such as years considered, language, publication status) to be used as criteria for eligibility for the review	Pg. 11 - 18
Information sources	9	Describe all intended information sources (such as electronic databases, contact with study authors, trial registers or other grey literature sources) with planned dates of coverage	Pg. 9
Search strategy	10	Present draft of search strategy to be used for at least one electronic database, including planned limits, such that it could be repeated	Supplements

For peer review only

Study records:			
Data management	11a	Describe the mechanism(s) that will be used to manage records and data throughout the review	Pg. 19
Selection process	11b	State the process that will be used for selecting studies (such as two independent reviewers) through each phase of the review (that is, screening, eligibility and inclusion in meta-analysis)	Pg. 19
Data collection process	11c	Describe planned method of extracting data from reports (such as piloting forms, done independently, in duplicate), any processes for obtaining and confirming data from investigators	Pg. 19 - 20
Data items	12	List and define all variables for which data will be sought (such as PICO items, funding sources), any pre-planned data assumptions and simplifications	Pg. 20 21
Outcomes and prioritization	13	List and define all outcomes for which data will be sought, including prioritization of main and additional outcomes, with rationale	Pg. 21
Risk of bias in individual studies	14	Describe anticipated methods for assessing risk of bias of individual studies, including whether this will be done at the outcome or study level, or both; state how this information will be used in data synthesis	Pg. 21
Data synthesis	15a	Describe criteria under which study data will be quantitatively synthesised	Pg. 22
	15b	If data are appropriate for quantitative synthesis, describe planned summary measures, methods of handling data and methods of combining data from studies, including any planned exploration of consistency (such as I^2, Kendall's τ)	Pg. 22 - 24
	15c	Describe any proposed additional analyses (such as sensitivity or subgroup analyses, meta-regression)	Pg. 23 - 24
	15d	If quantitative synthesis is not appropriate, describe the type of summary planned	n/a
Meta-bias(es)	16	Specify any planned assessment of meta-bias(es) (such as publication bias across studies, selective reporting within studies)	Pg. 21
Confidence in cumulative evidence	17	Describe how the strength of the body of evidence will be assessed (such as GRADE)	n/a

*** It is strongly recommended that this checklist be read in conjunction with the PRISMA-P Explanation and Elaboration (cite when available) for important clarification on the items. Amendments to a review protocol should be tracked and dated.** **The copyright for PRISMA-P (including checklist) is held by the PRISMA-P Group and is distributed under a Creative Commons Attribution Licence 4.0.**

From: Shamseer L, Moher D, Clarke M, Ghersi D, Liberati A, Petticrew M, Shekelle P, Stewart L, PRISMA-P Group. Preferred reporting items for systematic review and meta-analysis protocols (PRISMA-P) 2015: elaboration and explanation. BMJ. 2015 Jan 2;349(jan02 1):g7647.

For peer review only

BMJ Open

Wellbeing-focused Media Literacy Interventions in Secondary Schools: A Systematic Review and Meta-analysis Protocol

Journal:	*BMJ Open*
Manuscript ID	bmjopen-2026-119555.R1
Article Type:	Protocol
Date Submitted by the Author:	15-May-2026
Complete List of Authors:	Tibbs, Maria; University of York, Psychology in Education Research Centre, Department of Education.<br>Francis, Gill ; University of York, Psychology in Education Research Centre, Department of Education.<br>Mountain, Jennifer ; University of York, Psychology in Education Research Centre, Department of Education.<br>Polizzi, Gianfranco ; University of Birmingham, Department of Linguistics and Communication<br>Bell, Beth T; University of York, Psychology in Education Research Centre, Department of Education.
**Primary Subject Heading**:	Mental health
Secondary Subject Heading:	Paediatrics
Keywords:	Meta-Analysis, Systematic Review, MENTAL HEALTH, Schools, Social Media

SCHOLARONE™
MANUSCRIPTS

Wellbeing-focused Media Literacy Interventions in Secondary Schools: A Systematic Review and Meta-analysis Protocol

Maria Tibbs[1], Gill Francis[1], Jennifer Mountain[1], Gianfranco Polizzi[2], Beth T. Bell[1*].

[1] Psychology in Education Research Centre, Department of Education, University of York, United Kingdom.

[2] Department of Linguistics and Communication, University of Birmingham, Birmingham, United Kingdom.

***Corresponding author**

Email: Beth.bell@york.ac.uk.

Keywords: Adolescent wellbeing; school-based interventions; media literacy; digital literacy; digital wellbeing; meta-analysis; systematic review.

Wordcount: 4,744

Abstract

Background

Media and digital technologies shape adolescent development across interconnected online and offline contexts, offering opportunities for learning and connection while also exposing young people to risks that can affect wellbeing. Wellbeing-focused media literacy interventions aim to support young people in understanding and managing how media and technology shape their experiences and wellbeing. Schools are well placed to deliver such interventions at scale through curricular integration. However, existing interventions vary widely in focus, design, and evaluation quality, making it difficult to determine what works and why. This review synthesises school-based wellbeing-focused media literacy interventions and evaluates their effectiveness across wellbeing outcomes.

Methods

A range of interdisciplinary databases (ACM Digital Library, British Education Index, CINAHL, ERIC, MEDLINE, PsycINFO, Scopus, Web of Science) will be searched, alongside targeted grey literature searches of internal evaluation reports and dissertations. Eligible studies will include school-based interventions for adolescents aged 11 to 18. Abstracts and full texts will be screened in Covidence, and relevant data extracted using an adapted TIDieR framework. A three-level random effects meta-analysis will estimate pooled effects across psychological, social, physical, and digital wellbeing domains. Narrative synthesis, informed by the adapted TIDieR framework, will explore intervention content, delivery, and theoretical foundations to identify mechanisms of change and sources of heterogeneity.

Discussion

This review will provide the first comprehensive synthesis of wellbeing-focused media literacy interventions, clarifying which interventions are effective and identifying potential mechanisms of change. Findings will inform future research, policy and practice.

Ethics and dissemination

Ethical approval is not required for this systematic review, as it will only include published data. The review findings will be disseminated through a peer-reviewed publication and conference presentations.

Strengths and Limitations of this study

- The protocol is preregistered on OSF and PROSPERO and follows PRISMA-P guidance, with a search strategy spanning multiple interdisciplinary databases across education, psychology, public health, and HCI, alongside grey literature.
- The review consolidates previously fragmented research by bringing together interventions targeting psychological, social, physical, and digital wellbeing associated with media and technology use within a single unified framework.
- Intervention characteristics will be coded using an adapted TIDieR framework, enabling identification of active components and mechanisms that may underpin effective wellbeing-focused media literacy interventions.
- Considerable variation in intervention content, delivery formats, and outcome measures is expected. To address this, three-level random-effects models and, where appropriate, separate meta-analyses will be conducted, though interpretation of pooled estimates may still be complicated.

Introduction

Rates of mental health difficulties among adolescents have risen globally in recent decades [1–3], accompanied by declines in feelings of happiness, and positive affect [4–6]. Most mental health difficulties emerge before the age of 25 [7], and in the United Kingdom one in five adolescents were reported to have a probable mental health disorder in 2023 [8]. At the same time, there has been a rapid growth in youth digital technology use, with adolescents in the United Kingdom spending an average of four to six hours per day online [9]. While these trends have unfolded concurrently, existing evidence suggests that technologies are not inherently harmful to young people, but rather associations between digital technology use and mental health are complex and shaped by a wide range of social, developmental, and contextual factors.

For contemporary adolescents, social life, learning, and self-expression are increasingly shaped by media and digital technologies, including social media platforms such as YouTube, TikTok, Snapchat, and WhatsApp [10–12]. These technologies provide opportunities for connection, creativity, and community. However, they can also expose young people to cyberbullying, harmful or distressing content, reinforce sedentary behaviours, and promote upward comparison, all of which may undermine mental health and wellbeing [8,13–17]. Supporting young people to develop capable and informed engagement with media and technology is therefore a pressing public health and educational concern [18].

Defining Wellbeing

Wellbeing is commonly defined as "*how people feel and how they function, both on a personal and a social level and how they evaluate their lives as a whole*" [19], though its conceptualisation and measurement vary considerably across the literature [20,21]. It is widely recognised as a multidimensional construct, encompassing psychological, physical and social domains [22]. Psychological wellbeing includes two components; subjective

For peer review only - http://bmjopen.bmj.com/site/about/guidelines.xhtml

(hedonic) wellbeing, such as life satisfaction and affect balance [23,24] and psychological (eudaimonic) wellbeing, which focuses on qualities associated with flourishing, such as purpose and personal growth [25,26]. Physical wellbeing is frequently indexed through engagement in health-promoting behaviours (e.g., physical activity; [27] and avoidance of health-risk behaviours (e.g., smoking and substance use; [28]. Social wellbeing captures the extent to which individuals feel integrated into and supported by their social networks and communities, encompassing experiences of belonging, acceptance, and positive relationships [29]. Wellbeing, at both the personal and social level, can also be measured using more objective indicators, including physical/mental health status, educational attainment, socioeconomic security, and housing quality [30].

As young people's everyday lives have become deeply intertwined with digital technologies, scholars have increasingly argued that wellbeing frameworks must also incorporate a digital dimension. Digital wellbeing has been defined as the "*subjective individual experience of optimal balance between the benefits and drawbacks obtained from mobile connectivity*" [31]. Promoting wellbeing in contemporary society therefore requires approaches that attend to multiple, interrelated dimensions of wellbeing and equip young people with the knowledge and skills to engage with media and technology in ways that support personal and social flourishing in an increasingly digital world.

Media Literacy Education

Media literacy education offers one potential pathway for promoting well-being among adolescents. Media literacy refers to the ability to access, analyse, evaluate, and create media, with an emphasis on critically understanding media messages and their underlying intentions [32,33]. As media environments have become increasingly digitised, this framing has been extended to include digital literacy, applying the same core functional, critical, and

socioemotional competencies to online media, platforms, and technologies, alongside related literacies such as information and AI literacy [34,35]. In this framing, digital literacy is typically understood as a context-specific subfield of media literacy, that encompasses the same underlying competencies. These include functional competencies such as searching for information, creating content, and navigating platforms [36] and critical competencies, such as evaluating online content and understanding how platform infrastructures and algorithms shape what users encounter and produce [37–39].

Accordingly, in this review we use the term *media literacy interventions* as an umbrella to refer to interventions grounded in media literacy frameworks and traditions, including those applied to digital contexts (e.g., AI literacy, social media literacy) and commonly described as digital literacy interventions. There is growing recognition that media literacy includes an integral wellbeing dimension, involving the socioemotional knowledge and skills needed to navigate the psychological demands of modern-day media and digital technology and to understand how these experiences can affect wellbeing [40]. Emerging evidence suggests that these functional, critical and socioemotional skills are needed to help young people use technology in ways that maintain, enhance, support and protect wellbeing [41–44].

Wellbeing-focused Media Literacy Interventions

Schools have become a key setting for initiatives aimed at improving young people's media literacy in relation to wellbeing, as they reach learners during formative developmental periods, enable integration into curricula, and allow delivery at scale [45]. In the United Kingdom, improving media and digital skills is now a governmental priority [46], and education on some online harms is mandated through the Relationships, Sex and Health Education curriculum [47].

Increasingly, a diverse range of interventions aim to foster media literacy in ways that support youth wellbeing in both online and offline contexts. For example, interventions have been developed to promote positive body image by fostering media literacy in relation to harmful appearance-related messaging across media and technology, while also teaching psychological and behavioural coping strategies [48,49]. Interventions have also been developed to promote social wellbeing in online spaces by providing education related to how the design features and social affordances of online media environments may shape and sustain cyberbullying behaviours and social relationships [50,51]. While these interventions vary in terms of content and approach, a commonality is that all aim to *develop the knowledge and skills needed to foster wellbeing in relation to media and technology* through three interrelated sets of competencies:

(1) Critical knowledge and skills to analyse and evaluate media and technology.

(2) Functional knowledge and skills to navigate and operate media and technology in autonomous ways.

(3) Socio-emotional knowledge and skills to manage personal and social wellbeing in relation to media and technology.

In many cases, wellbeing is positioned as an assumed or downstream outcome arising from the development and application of these competencies, rather than as an explicit focus of intervention theory or evaluation. We refer to this diverse group of approaches as *wellbeing-focused media literacy interventions*.

For peer review only - http://bmjopen.bmj.com/site/about/guidelines.xhtml

Despite growing recognition of their educational and public health relevance [52], little is known about which wellbeing-focused media literacy interventions work, for whom, and why. Existing reviews have largely examined intervention effectiveness within specific domains, such as cyberbullying [53] and body image [54], even though interventions often share elements that promote critical awareness, self-reflection, and behavioural change [55]. This fragmentation prevents understanding of shared mechanisms or "active ingredients" that drive positive outcomes across intervention types and limits understanding of what approaches are most effective or transferable to real-world educational settings.

Objectives

Accordingly, this systematic review aims to identify classroom-based wellbeing-focused media literacy interventions that are effective among adolescents aged 11 to 18. Furthermore, it aims to examine the components and characteristics that underpin effectiveness. Specifically, it addresses the following questions:

1. Do school-based wellbeing-focused media literacy interventions improve youth wellbeing outcomes (psychological/subjective, social, physical, and digital)?

2. What active ingredients (components & characteristics) underpin effective school-based well-being focused media literacy interventions?

Materials and Method

This study will conduct a systematic review and meta-analysis examining the effectiveness of eligible interventions. The review will include experimental and quasi-experimental studies, including dissertations employing these designs, provided they meet the predefined eligibility criteria. Where sufficient comparable data are available, quantitative findings from eligible studies will be synthesised using meta-analysis to estimate the effects of the interventions under investigation.

Protocol and Registration

The current protocol is aligned with the Preferred Reporting Items for Systematic Reviews and Meta-Analyses Protocols (PRISMA-P; [56], see supplementary materials. The subsequent systematic review and meta-analysis will be reported following the Preferred Reporting Items for Systematic Reviews and Meta-Analyses (PRIMSA; [57].

This protocol has been preregistered on the Open Science Framework Platform (OSF; 10.17605/OSF.IO/APM7F) and PROSPERO (CRD420251269724). Any significant amendments made to the protocol will be documented and reported in the final systematic review manuscript. Database searches have been conducted, and title and abstract screening is currently ongoing, with completion anticipated by November 2026.

Information Sources and Search Strategy

The search will include both peer-reviewed and grey literature published in English, with no geographic restrictions. Peer-reviewed studies will be identified through interdisciplinary databases to ensure comprehensive coverage across education, psychology, public health and human computer interaction: ACM Digital Library, British Education Index, CINAHL, ERIC (Education Resources Information Centre), MEDLINE, PsycINFO, Scopus, Web of Science. Grey literature will include doctoral theses and publicly available internal reports evaluating

For peer review only - http://bmjopen.bmj.com/site/about/guidelines.xhtml

interventions with randomised designs, as these sources can provide valuable, otherwise unpublished evidence subjected to some level of independent scrutiny. Doctoral theses will be identified through ProQuest Dissertations and Theses Global. The websites of reputable media literacy and/or educational evaluation organisation websites (e.g., Education Endowment Foundation, Internet Matters, PSHE Association) will be searched for relevant internal evaluation reports. The rationale for including each database is presented in **Table 1**. To ensure all relevant articles will be captured, a process of forward reference and backward citation checking using *citationchaser* [58] will be conducted on eligible full texts.

The search strategy was developed in collaboration with a library specialist at the University of York and through examination of previous systematic reviews in the areas of digital literacy, media literacy, and wellbeing. Search terms focused on the core domains under investigation: the population of interest, characteristics of the intervention, relevant outcomes, the setting within which interventions are implemented, and study design, as well as MeSH terms or subject areas relevant to each database where applicable. Considering the breadth of potential wellbeing outcomes, the search strategy was conducted separately for each of the four wellbeing domains (psychological/subjective, social, physical, and digital) to ensure sufficient and relevant retrieval of studies. See **Supplementary Material 1** for an example SCOPUS search strategy.

Table 1.

Rationale for bibliographic databases

**Database**	**Rationale**

ERIC (Education Resources Information Centre)	Captures literature focused on education and learning contexts.
British Education Index	Covers educational literature focused within the British context, that may not be captured within ERIC.
Web of Science	Broad, multidisciplinary database indexing a wide range of high-impact peer-reviewed research.
PsycINFO	Specialises in psychology and related behavioural sciences.
CINAHL	Covers nursing, allied health, and applied healthcare research.
MEDLINE	Biomedical and public health database; includes extensive coverage of health interventions and uses MeSH terms, enhancing search precision.
SCOPUS	Indexes a broad range of journals across health sciences, technology, and education.
ACM Digital Library	Houses studies from human–computer interaction, a field that often contributes to research on digital literacy and technology use.

For peer review only - http://bmjopen.bmj.com/site/about/guidelines.xhtml

ProQuest Dissertations and Theses Global	Grey literature database that houses doctoral theses.

Eligibility Criteria

To be included in the current systematic review, all studies, including those identified across the grey literature, will need to meet the pre-defined inclusion and exclusion criteria as informed by the Population, Intervention, Comparison, Outcome, Study Design (PICOS) model. For a summary of the inclusion criteria, **see Table 2.**

Population

To be included in this review, studies must focus on young people engaged in formal second level education aged 11- 18. This age range was selected to capture developmental stages typically corresponding to secondary-level schooling across international education systems, while excluding primary and post-secondary contexts.

Intervention

Eligible interventions must include a media literacy component, defined as educational content focused on developing adolescents' ability to access, analyse, evaluate, and create media, with an emphasis on critically understanding media messages and their underlying intentions [32,33]. Media or technology must be an active target of the intervention, rather than solely the mode of delivery.

Interventions may contain one or more of the following media literacy components, which reflect different ways in which wellbeing-focused media literacy is operationalised in practice:

1. **Critical:** The knowledge and skills needed to critically analyse and evaluate media and technology through a wellbeing lens (e.g. understanding persuasive intent, platform design, algorithms, or how media features shape experiences relevant to wellbeing).
2. **Functional:** The knowledge and skills needed to navigate and operate media and technology in ways that may support wellbeing, including modifying engagement, using safety and support features, and enacting boundaries or help-seeking behaviours within digital environments.
3. **Socio-emotional:** The knowledge and skills needed to recognise and reflect on emotional and social responses to media and technology, and to evaluate when media engagement supports or undermines wellbeing.

Comparators

Studies will be included if they evaluate the intervention against either a passive control condition (e.g., no intervention, usual care, or waitlist/delayed care) or an active control condition (e.g., an alternative intervention or the same intervention delivered at a different time point). Studies that do not include a comparison or control group, such as pre–post, uncontrolled, or purely observational designs, will be excluded.

Outcomes

The primary outcome is wellbeing, which we define broadly as "*how people feel and how they function, both on a personal and a social level, and how they evaluate their lives as a whole*" [19]. To structure our analysis, we draw on the World Health Organisation's three-domain model of wellbeing, which includes psychological, social, and physical dimensions [22]. We extend this framework by adding a fourth domain: digital wellbeing. This expanded model allows us to capture the full spectrum of experiences and risks associated with young

people's digital lives. For the purposes of this review, we define these four domains as follows:

1. ***Psychological/Subjective wellbeing*** reflects individuals' evaluations and lived experiences of their own lives, including their sense of life satisfaction, experiences of positive and negative emotions, self-concept, the absence of mental health difficulties, and overall capacity to flourish [59,60].
2. ***Social wellbeing*** refers to the extent to which individuals feel integrated into and supported by their social networks and communities [29]. This can include concepts such as belonging, friendship quality, as well as experiences of social isolation and loneliness.
3. ***Physical wellbeing*** can be described as the ability to maintain bodily health and energy through balanced behaviours such as exercise, diet, sleep, sexual health, and avoidance of harmful habits [61,62].
4. ***Digital wellbeing*** refers to individuals' ability to engage with digital technologies in ways that support wellbeing, including feeling safe, in control, and supported in online environments, as well as minimising exposure to digital risks such as harmful content, excessive use, or negative social interactions.

Secondary outcomes include indicators of digital, media, and information literacy, reflecting young people's knowledge, skills, and critical competencies in navigating online environments. For detail on the categorisation and representative measures, see **Supplementary Material 2 and 3.**

Study Design

This review will include English language, peer-reviewed experimental and quasi-experimental studies with a comparator or control group, including randomised controlled

For peer review only - http://bmjopen.bmj.com/site/about/guidelines.xhtml

280 trials (cluster and individual) and non-randomised comparison designs (e.g. matched or non-
281 equivalent control groups). Eligible studies must provide a quantitative comparison between
282 intervention and control conditions to assess effectiveness or efficacy.

283 ***Timeframe***

284 The search period was set from 2000 to the present date to capture developments in digital
285 literacy during a transitional period of conceptual overlap with traditional media literacy,
286 when technologies were beginning to be integrated into media literacy education and
287 regulation [63]. These developments are exemplified by the enactment of international
288 policies such as the U.S. Children's Internet Protection Act (2000), the UK Communications
289 Act (2003), and the EU Action Plan for a Safer Internet (1999-2004), which increasingly
290 framed media literacy around online safety, responsible use, and digital participation
291 alongside traditional media.

For peer review only - http://bmjopen.bmj.com/site/about/guidelines.xhtml

Table 2. *Inclusion/Exclusion Criteria*

	**Inclusion**	**Exclusion**
Population	Young people aged 11-18 attending post-primary, secondary, or high school.	Young people not attending post-primary, secondary, or high school.<br>Young people aged <11 or >18.
Intervention	● School or classroom-based media literacy interventions that include education on the knowledge and skills needed to foster wellbeing in relation to media and technology including critical, functional and socio-emotional.	Interventions that:<br>● Are not conducted within education settings.<br>● Do not target students' knowledge and skills related to media and technology.
Comparator	Studies will be included if they compare the intervention with either a passive control (e.g., no intervention, usual care, or waitlist/delayed care) or active control (e.g., an alternative intervention or the same intervention delivered at a different time point).	Studies without a comparison or control group (e.g., pre–post, uncontrolled, or observational designs) will be excluded.

Outcome	Studies will be included if their primary or secondary outcomes relate to either wellbeing and/or digital/media/information literacy.	● Studies will be excluded if they do not assess wellbeing as an outcome
Study Design	Studies will be included if they:<br>● Employ experimental designs with a comparator group, randomised controlled trials (including cluster-RCTs) and non-randomised comparison group designs (e.g. quasi-experiments using matched control or non-equivalent control group).<br>● Include a quantitative comparison between intervention and control or comparison conditions to evaluate effectiveness or efficacy.	Studies will be excluded if they:<br>● Do not include a comparator group (e.g. single-group pre–post, purely observational, or cross-sectional studies).<br>● Employ qualitative designs.<br>● Are systematic reviews and meta-analyses.<br>● Are intervention protocols without published findings available at the time of synthesis.
Timeframe	2000 to present.	Studies published before the year 2000.

Data Management

Search results will be exported to Zotero reference managers and duplicates will be removed. Covidence systematic review software will be used to screen the abstracts and full texts of identified studies. Microsoft Excel will be used to extract relevant study data and to conduct quality appraisals of all included studies.

Selection of Studies and Data Extraction

Following the removal of duplicates, studies will be screened in two stages using Covidence. First, titles and abstracts will be assessed against the inclusion and exclusion criteria. Full texts of potentially relevant studies will then be retrieved and reviewed for eligibility. Four reviewers experienced in systematic review methodologies will contribute to the data screening and extraction process. At each stage studies will be double screened, and discrepancies will be resolved through a joint discussion by the two reviewers, and the opinion of a third reviewer will be obtained if agreement is not reached. Inter-rater reliability (e.g., Cohen's κ) will be calculated to assess agreement between the two reviewers at title and abstract screening as well as full-text screening.

Similarly, two reviewers will duplicate data extraction and coding to ensure accuracy and consistency. One reviewer will extract, and code all included studies, while the remaining three reviewers will each extract and code a proportion of the studies to enable cross-checking and verification. Extracted data will be compared for accuracy and completeness, with any discrepancies resolved through discussion and arbitration by a fourth author where necessary.

A pre-piloted data extraction form will be used to capture bibliographic details, sample characteristics, intervention and comparison conditions, and quantitative outcomes from each included study. The form will capture:

For peer review only - http://bmjopen.bmj.com/site/about/guidelines.xhtml

1. **Key study characteristics:** including author details, study date, country, study aims and objectives.

2. **Participant and School characteristics:** including sample size and composition (e.g., gender distribution, mean age, type of school [public/private], and contextual factors such as rural/urban setting of the school).

3. **Intervention characteristics:** We will record intervention characteristics using an adapted version of the Template for Intervention Description and Replication (TIDieR) framework [64]. Using this, we will extract details on:

   a. The rationale, goal, and theoretical framing of the intervention.

   b. What is delivered (specific activities, and intervention components). This will be extended to capture which wellbeing-focused media literacy components are targeted. These components include: (1) critical knowledge and skills; (2) functional knowledge and skills; and (3) socio-emotional knowledge and skills. We acknowledge that these domains are interrelated and will not mutually exclusive. The coding framework will therefore allow for dual or multiple categorisations where components span domains.

   c. Who the intervention is delivered by (e.g., teachers, trained facilitators, peers).

   d. How the intervention was delivered (in-class online modules, group activities, presentations). This will be extended to extract details on mechanisms through which these interventions are theorised to achieve their outcomes. Specifically, we will code for teaching strategies and pedagogical approaches (e.g., reflection, discussion, experiential learning) and behaviour change techniques (BCT's; [65] used to enact behaviour change.

For peer review only - http://bmjopen.bmj.com/site/about/guidelines.xhtml

e. Where the intervention was delivered (e.g., within subject specific classes, after school, during pastoral care).

f. The frequency and duration of the intervention session(s).

g. Evidence of any modifications during the process of intervention implementation.

h. Details on adherence to any existing protocols.

i. Details of any adaptations or tailoring for different contexts.

4. **Outcomes:** including outcome domains, measures used, time points of assessment (e.g., pre-, post-, follow-up), directionality of wellbeing outcomes (positive or negative), and available relevant quantitative data (means, related effect sizes, and associated variance estimates) for meta-analytic calculations.

Quality Appraisal and Certainty of Evidence

To examine risk of bias across studies (including those identified through grey literature searching), the Cochrane Risk of Bias Tool (RoB2; [66] will be used. This tool evaluates the risk of bias by assigning studies a quality rating of low, high, or unclear across several domains of bias. Studies deemed low quality will be retained for both the systematic review and meta-analysis. However, in line with recent meta-analyses of school-based interventions [67,68], sensitivity analyses will be conducted excluding low-quality studies to examine their influence on the magnitude and robustness of the overall effect. Funnel plots will be used to assess potential publication bias, and Egger's test will be employed to statistically evaluate the presence of small-study effects.

For peer review only - http://bmjopen.bmj.com/site/about/guidelines.xhtml

Data Synthesis

Narrative Synthesis

Extracted data will be tabulated and narratively synthesised following Popay et al's. [69] guidance on narrative synthesis. Firstly, study, participant, and intervention characteristics will be summarised descriptively using the adapted TIDieR framework. Second, interventions will be compared according to their wellbeing-focused media literacy components (knowledge/understanding and skills) and the mechanisms of change identified (teaching strategies, pedagogies, and behaviour change techniques). Finally, the synthesis will explore how variations in these components and mechanisms related to wellbeing outcomes across domains (psychological/subjective, social, physical, digital). Using this approach, the synthesis will provide an overview of patterns in intervention content (what) and the mechanisms to enact behaviour change (how), providing a working theory of how the intervention works to achieve its outcomes [70].

Meta-analysis

All studies meeting the inclusion criteria will be included in the systematic review; however, only studies reporting sufficient quantitative data on wellbeing outcomes (e.g., means and standard deviations, change scores, or effect estimates with variance) will be eligible for inclusion in the meta-analysis. Studies lacking extractable data or reporting outcomes that cannot be meaningfully standardised will be synthesised narratively. Where outcomes are reported on continuous scales, effect sizes are expected to be calculated as standardised mean differences (Hedges' g), which is commonly used in meta-analyses of school-based interventions to accommodate heterogeneous measures and small sample sizes. Effect sizes will be interpreted in relation to those reported in prior meta-analyses of school-based interventions, with emphasis on robustness and practical relevance rather than fixed magnitude thresholds.

For peer review only - http://bmjopen.bmj.com/site/about/guidelines.xhtml

We will perform a meta-analysis using an intention-to-treat approach, as opposed to a per-protocol approach, to minimise post-randomisation bias and overestimation of effects [71]. This means that all participants originally allocated to intervention and control groups will be analysed according to their initial assignment, regardless of adherence or dropout, ensuring a more conservative and unbiased estimate of effect. To account for probable correlation and dependence between multiple effect sizes reported within individual studies, we will adopt a three-level random-effects meta-analysis to estimate pooled effects. A three-level random-effects meta-analysis partitions variance into three components:

1) Sampling variance associated each observed effect size (sampling error within studies).
2) Variance between effect sizes within the same study
3) Variance between study effects.

In this analysis, effect sizes are nested within studies, and multiple outcomes reported by the same study are modelled as correlated at level two. This approach allows the inclusion of several non-independent outcomes per study while appropriately accounting for their clustering [72,73].

To assess what proportion of the observed variance reflects variance in true effects, the I^2 statistic will be calculated, with cut-offs of 25, 50, and 75% denoting low, moderate, and high levels of heterogeneity. 95% confidence intervals and for the I^2 values will be calculated using the method outlined by Borenstein [74]. Finally, to explore sources of heterogeneity, we will conduct moderator analyses. Moderators will include study design characteristics (e.g., passive versus active control conditions, publication year), intervention characteristics (e.g., mode of delivery, number and duration of modules or lessons), and participant characteristics (e.g., gender composition). In addition, outcome domains (psychological/subjective, social, physical, digital) will be entered as categorical moderators

For peer review only - http://bmjopen.bmj.com/site/about/guidelines.xhtml

alongside study-level characteristics such as intervention design and participant details. This will allow us to formally test whether effects differ across outcome domains, as well as to examine potential sources of between-study heterogeneity. Following guidance from [75], analyses with categorical moderators will only be conducted if each category contains at least five cases.

As a robustness check, will fit a correlated-hierarchical effects model (CHE), which combines a hierarchical variance structure with robust variance estimation to permit inference without requiring known within-study correlations [76]. This will be accompanied by further sensitivity analyses using examination of the potential impact of outliers and risk of bias. Where substantial or unforeseen heterogeneity is identified, and where sufficient data are available, we may additionally conduct separate three-level or standard meta-analyses within wellbeing domains (e.g., psychological/subjective, social, physical, digital) to improve conceptual and methodological comparability of pooled estimates. Importantly, meta-analytic decisions will be guided by theoretical coherence, measurement comparability, and the distribution of available data within each domain. Where measures within a domain are conceptually or operationally heterogeneous, outcomes that cannot be meaningfully compared will be synthesised narratively rather than pooled.

Statistical analyses will be conducted using the *metaSEM* package [77] in R statistical software [78].

Ethics and Dissemination

Ethical approval is not required for this systematic review, as it will only include published data. The review findings will be disseminated through a peer-reviewed publication and conference presentations.

Public and Patient Involvement (PPI)

For peer review only - http://bmjopen.bmj.com/site/about/guidelines.xhtml

The current protocol has been presented and reviewed by $N = 10$ key stakeholders including as representatives from charities and advocacy groups, education specialists, methodological experts, and academic researchers as well as $N = 2$ young people.

Deviations from the Protocol

Any deviations from the registered protocol will be updated and recorded on the OSF and Prospero websites.

Discussion

Existing systematic reviews have largely examined the effectiveness of interventions targeting specific online harms [53,79,80], yet few have integrated and consolidated these within a broader wellbeing framework [81]. To date, no review has been conducted examining the effectiveness of wellbeing-focused media literacy interventions. To date, no meta-analysis has examined the effectiveness of such interventions. Furthermore, no work has been done to visualise the body of evidence or research gaps relevant to wellbeing-focused media literacy interventions.

The proposed review will address this gap by synthesising evidence across school-based interventions that build young people's understanding of how digital experiences affect wellbeing and the skills needed to support it. The three-level meta-analysis will provide pooled estimates across psychological/subjective, social, physical, and digital wellbeing outcomes, while the accompanying narrative synthesis will identify active components and contextual factors shaping effectiveness. Findings will clarify how wellbeing-focused media literacy can be defined, implemented, and evaluated in educational settings, advancing both theoretical and practical understanding of how schools can support youth wellbeing through media and digital literacy programmes.

For peer review only - http://bmjopen.bmj.com/site/about/guidelines.xhtml

Conflicts of Interest

The authors declare no conflicts of interest.

Acknowledgements

The authors would like to thank members of both the steering committee and the youth advisory board for their invaluable contributions to the drafting of this manuscript.

Funding

This project has been funded by the Nuffield Foundation (Grant Number: EDO /FR-000025035), but the views expressed are those of the authors and not necessarily the Foundation. Visit nuffieldfoundation.org

Author Contributions

- **MT:** Conceptualisation, Methodology, Formal analysis plan, Investigation, Writing – original draft.
- **GF:** Conceptualisation, Methodology, Validation, Writing – review & editing.
- **JM:** Investigation, Data curation, Writing – review & editing.
- **GP:** Conceptualisation, Writing – review & editing.
- **BB:** Conceptualisation, Funding acquisition, Supervision, Writing – review & editing.

References

1 Cybulski L, Ashcroft DM, Carr MJ, *et al.* Temporal trends in annual incidence rates for psychiatric disorders and self-harm among children and adolescents in the UK, 2003–2018. *BMC Psychiatry*. 2021;21:229. doi: 10.1186/s12888-021-03235-w

For peer review only - http://bmjopen.bmj.com/site/about/guidelines.xhtml

2 Fitzgerald A, Mahon C, Shevlin M, *et al.* Exploring changing trends in depression and anxiety among adolescents from 2012 to 2019: Insights from My World repeated cross-sectional surveys. *Early Interv Psychiatry*. 2025;19:e13562. doi: 10.1111/eip.13562

3 Mojtabai R, Olfson M. National Trends in Mental Health Care for US Adolescents. *JAMA Psychiatry*. 2020;77:703–14. doi: 10.1001/jamapsychiatry.2020.0279

4 Blanchflower DG, Bryson A, Xu X. The declining mental health of the young and the global disappearance of the unhappiness hump shape in age. *PLOS ONE*. 2025;20:e0327858. doi: 10.1371/journal.pone.0327858

5 Dumuid D, Singh B, Brinsley J, *et al.* Trends in Well-Being Among Youth in Australia, 2017-2022. *JAMA Netw Open*. 2023;6:e2330098. doi: 10.1001/jamanetworkopen.2023.30098

6 Marquez J, Long E. A Global Decline in Adolescents' Subjective Well-Being: a Comparative Study Exploring Patterns of Change in the Life Satisfaction of 15-Year-Old Students in 46 Countries. *Child Indic Res*. 2021;14:1251–92. doi: 10.1007/s12187-020-09788-8

7 Solmi M, Radua J, Olivola M, *et al.* Age at onset of mental disorders worldwide: large-scale meta-analysis of 192 epidemiological studies. *Mol Psychiatry*. 2022;27:281–95.

8 NHS Digital. Mental Health of Children and Young People in England, 2023 - wave 4 follow up to the 2017 survey. 2023.

9 Ofcom. Online Nation Report. 2023.

10 Charmaraman L, Hodes R, Richer AM. Young sexual minority adolescent experiences of self-expression and isolation on social media: cross-sectional survey study. *JMIR Ment Health*. 2021;8:e26207.

11 Goodyear VA, Armour KM. Young People's health-related learning through social media: What do teachers need to know? *Teach Teach Educ*. 2021;102:103340.

12 Mittmann G, Woodcock K, Dörfler S, *et al.* "TikTok Is My Life and Snapchat Is My Ventricle": A Mixed-Methods Study on the Role of Online Communication Tools for Friendships in Early Adolescents. *J Early Adolesc*. 2022;42:172–203. doi: 10.1177/02724316211020368

13 Al-Jbouri E, Volk AA, Spadafora N, *et al.* Friends, followers, peers, and posts: adolescents' in-person and online friendship networks and social media use influences on friendship closeness via the importance of technology for social connection. *Front Dev Psychol*. 2024;2:1419756.

14 Caba-Machado V, Mcilroy D, Padilla-Adamuz FM. Night-time use of electronic devices, fear of missing out, sleep difficulties, anxiety, and well-being in UK and Spain: a cross-cultural comparison. *Curr Psychol*. 2024;43:21134–45.

For peer review only - http://bmjopen.bmj.com/site/about/guidelines.xhtml

15 Engberg E, Leppänen MH, Sarkkola C, *et al.* Physical Activity Among Preadolescents Modifies the Long-Term Association Between Sedentary Time Spent Using Digital Media and the Increased Risk of Being Overweight. Published Online First: 29 July 2021. doi: 10.1123/jpah.2021-0163

16 Peppler K, Dahn M. Social Media and Creativity. *The Routledge International Handbook of Children, Adolescents, and Media*. Routledge 2022.

17 Zheng Q, Yang W, Li T, *et al.* Examining the Pathways to Social Media FOMO: Mediation Effects of Passive Engagement and Upward Social Comparisons. *Int J Human–Computer Interact*. 2025;1–8. doi: 10.1080/10447318.2025.2499156

18 Büchi M. Digital well-being theory and research. *New Media Soc*. 2024;26:172–89. doi: 10.1177/14614448211056851

19 Michaelson J, Mahony S, Schifferes J. Measuring wellbeing: A guide for practitioners. *New Econ Found Lond*. 2012.

20 Dodge R, Daly A, Huyton J, *et al.* The challenge of defining wellbeing. Intnl. J. Wellbeing 2, 222–235. 2012.

21 Hone L, Schofield G, Jarden A. Conceptualizations of wellbeing: Insights from a prototype analysis on New Zealand workers. *N Z J Hum Resour Manag*. 2015;15:97–118.

22 World Health Organization. Summary reports on proceedings minutes and final acts of the international health conference held in New York from 19 June to 22 July 1946. *N Y WHO*. 1948;35.

23 Diener E, Suh EM, Lucas RE, *et al.* Subjective well-being: three decades of progress. *Psychol Bull*. 1999;125:276.

24 Diener E, Ryan K. Subjective well-being: A general overview. *South Afr J Psychol*. 2009;39:391–406.

25 Ryan RM, Deci EL. On Happiness and Human Potentials: A Review of Research on Hedonic and Eudaimonic Well-Being. *Annu Rev Psychol*. 2001;52:141–66. doi: 10.1146/annurev.psych.52.1.141

26 Ryff CD, Singer BH. Know Thyself and Become What You Are: A Eudaimonic Approach to Psychological Well-Being. *J Happiness Stud*. 2008;9:13–39. doi: 10.1007/s10902-006-9019-0

27 Hennessey A, MacQuarrie S, Petersen KJ. Exploring physical, subjective and psychological wellbeing profile membership in adolescents: a latent profile analysis. *BMC Psychol*. 2024;12:720.

28 Dittmar H, Bond R, Hurst M, *et al.* The relationship between materialism and personal well-being: A meta-analysis. *J Pers Soc Psychol*. 2014;107:879–924. doi: 10.1037/a0037409

For peer review only - http://bmjopen.bmj.com/site/about/guidelines.xhtml

29 Keyes CLM. Social well-being. *Soc Psychol Q*. 1998;121–40.

30 Organisation for Economic Co-operation and Development Secretary-General, OECD iLibrary. How's life?: measuring well-being. Paris 2011.

31 Vanden Abeele MMP. Digital Wellbeing as a Dynamic Construct. *Commun Theory*. 2021;31:932–55. doi: 10.1093/ct/qtaa024

32 Aufderheide P. Media Literacy: From a Report of the National Leadership Conference on Media Literacy. *Media Literacy Around the World*. Routledge 1997.

33 Hobbs R. Digital and Media Literacy: A Plan of Action. A White Paper on the Digital and Media Literacy Recommendations of the Knight Commission on the Information Needs of Communities in a Democracy. Aspen Institute 2010.

34 Leaning M. An approach to digital literacy through the integration of media and information literacy. *Media Commun*. 2019;7:4–13.

35 Wuyckens G, Landry N, Fastrez P. Untangling media literacy, information literacy, and digital literacy: A systematic meta-review of core concepts in media education. *J Media Lit Educ*. 2022;14:168–82. doi: https://doi.org/10.23860/JMLE-2022-14-1-12

36 Van Deursen AJ, Helsper EJ, Eynon R. Development and validation of the Internet Skills Scale (ISS). *Inf Commun Soc*. 2016;19:804–23.

37 Buckingham D. Digital Media Literacies: rethinking media education in the age of the Internet. *Res Comp Int Educ*. 2007;2:43–55.

38 Dezuanni M. Minecraft and children's digital making: implications for media literacy education. *Learn Media Technol*. 2018;43:236–49.

39 Polizzi G. Internet users' utopian/dystopian imaginaries of society in the digital age: Theorizing critical digital literacy and civic engagement. *New Media Soc*. 2023;25:1205–26.

40 Tuamsuk K, Subramaniam M. The current state and influential factors in the development of digital literacy in Thailand's higher education. *Inf Learn Sci*. 2017;118:235–51. doi: 10.1108/ILS-11-2016-0076

41 Gordon CS, Ferber K, Notley T, *et al.* The relationship between media literacy and well-being: A systematic review and meta-analysis. *Educ Res Rev*. 2025;49:100731. doi: 10.1016/j.edurev.2025.100731

42 Kalmus V, Tambaum T, Abuladze L. Use of digital tools, digital skills and mental well-being. 2023:235–50.

43 Livingstone S, Mascheroni G, Stoilova M. The outcomes of gaining digital skills for young people's lives and wellbeing: A systematic evidence review. *New Media Soc*. 2023;25:1176–202.

For peer review only - http://bmjopen.bmj.com/site/about/guidelines.xhtml

44 Vissenberg J, De Coninck D, Mascheroni G, *et al.* Digital Skills and Digital Knowledge as Buffers Against Online Mis/Disinformation? Findings from a Survey Study Among Young People in Europe. *Soc Media Soc*. 2023;9:20563051231207859. doi: 10.1177/20563051231207859

45 Carta MG, Di Fiandra T, Rampazzo L, *et al.* An overview of international literature on school interventions to promote mental health and well-being in children and adolescents. *Clin Pract Epidemiol Ment Health CP EMH*. 2015;11:16.

46 DCMS. Department for Digital, Culture, Media and Sport: Annual Report and Accounts. 2022.

47 Department for Education. Teaching Online Safety in Schools. 2023.

48 Bell BT, Taylor C, Paddock D, *et al.* Digital Bodies: A controlled evaluation of a brief classroom-based intervention for reducing negative body image among adolescents in the digital age. *Br J Educ Psychol*. 2022;92:280–98.

49 Gordon CS, Jarman HK, Rodgers RF, *et al.* Outcomes of a cluster randomized controlled trial of the SoMe social media literacy program for improving body image-related outcomes in adolescent boys and girls. *Nutrients*. 2021;13:3825.

50 Kutok ER, Dunsiger S, Patena JV, *et al.* A Cyberbullying Media-Based Prevention Intervention for Adolescents on Instagram: Pilot Randomized Controlled Trial. *JMIR Ment Health*. 2021;8:e26029. doi: 10.2196/26029

51 Schoeps K, Villanueva L, Prado-Gascó VJ, *et al.* Development of Emotional Skills in Adolescents to Prevent Cyberbullying and Improve Subjective Well-Being. *Front Psychol*. 2018;9. doi: 10.3389/fpsyg.2018.02050

52 Ofcom. A Positive Vision for Media Literacy: Ofcom's Three-Year Media Literacy Strategy. 2024.

53 Ng ED, Chua JYX, Shorey S. The effectiveness of educational interventions on traditional bullying and cyberbullying among adolescents: A systematic review and meta-analysis. *Trauma Violence Abuse*. 2022;23:132–51.

54 Zuair AA, Sopory P. Effects of media health literacy school-based interventions on adolescents' body image concerns, eating concerns, and thin-internalization attitudes: A systematic review and meta-analysis. *Health Commun*. 2022;37:20–8.

55 Weinstein E, James C. School-based initiatives promoting digital citizenship and healthy digital media use. *Handb Adolesc Digit Media Use Ment Health*. 2022;365.

56 Moher D, Shamseer L, Clarke M, *et al.* Preferred reporting items for systematic review and meta-analysis protocols (PRISMA-P) 2015 statement. *Syst Rev*. 2015;4:1. doi: 10.1186/2046-4053-4-1

For peer review only - http://bmjopen.bmj.com/site/about/guidelines.xhtml

57 Page MJ, McKenzie JE, Bossuyt PM, *et al.* The PRISMA 2020 statement: an updated guideline for reporting systematic reviews. *BMJ*. 2021;372:n71. doi: 10.1136/bmj.n71

58 Haddaway NR, Grainger MJ, Gray CT. Citationchaser: A tool for transparent and efficient forward and backward citation chasing in systematic searching. *Res Synth Methods*. 2022;13:533–45.

59 Adler A, Unanue W, Osin E, *et al.* Psychological wellbeing. *Happiness*. 2017;118.

60 Stone AA, Mackie C, Framework P on MSW-B in a P-R, *et al.* Introduction. *Subjective Well-Being: Measuring Happiness, Suffering, and Other Dimensions of Experience [Internet]*. National Academies Press (US) 2013.

61 Harvard University Health Sciences. Physical – Center for Wellness and Health Promotion. n/d.

62 Hung S-T, Cheng Y-C, Wu C-C, *et al.* Examining Physical Wellness as the fundamental element for achieving holistic well-being in older persons: review of literature and practical application in Daily Life. *J Multidiscip Healthc*. 2023;1889–904.

63 Wallis R, Buckingham D. Media literacy: the UK's undead cultural policy. *Int J Cult Policy*. 2019;25:188–203. doi: 10.1080/10286632.2016.1229314

64 Hoffmann TC, Glasziou PP, Boutron I, *et al.* Better reporting of interventions: template for intervention description and replication (TIDieR) checklist and guide. *BMJ*. 2014;348:g1687. doi: 10.1136/bmj.g1687

65 Michie S, Richardson M, Johnston M, *et al.* The behavior change technique taxonomy (v1) of 93 hierarchically clustered techniques: building an international consensus for the reporting of behavior change interventions. *Ann Behav Med*. 2013;46:81–95.

66 Higgins J. Cochrane handbook for systematic reviews of interventions. Version 5.1. 0 [updated March 2011]. The Cochrane Collaboration. *Www Cochrane-Handb Org*. 2011.

67 Hodder RK, O'Brien KM, Lorien S, *et al.* Interventions to prevent obesity in school-aged children 6-18 years: An update of a Cochrane systematic review and meta-analysis including studies from 2015–2021. *eClinicalMedicine*. 2022;54. doi: 10.1016/j.eclinm.2022.101635

68 Zhang Q, Wang J, Neitzel A. School-based Mental Health Interventions Targeting Depression or Anxiety: A Meta-analysis of Rigorous Randomized Controlled Trials for School-aged Children and Adolescents. *J Youth Adolesc*. 2023;52:195–217. doi: 10.1007/s10964-022-01684-4

69 Popay J, Roberts H, Sowden A, *et al.* Guidance on the conduct of narrative synthesis in systematic reviews. *Prod ESRC Methods Programme Version*. 2006;1:b92.

For peer review only - http://bmjopen.bmj.com/site/about/guidelines.xhtml

70 Rodgers M, Sowden A, Petticrew M, *et al.* Testing methodological guidance on the conduct of narrative synthesis in systematic reviews: effectiveness of interventions to promote smoke alarm ownership and function. *Evaluation*. 2009;15:49–73.

71 McCoy CE. Understanding the Intention-to-treat Principle in Randomized Controlled Trials. *West J Emerg Med*. 2017;18:1075–8. doi: 10.5811/westjem.2017.8.35985

72 Cheung MW-L. Modeling dependent effect sizes with three-level meta-analyses: A structural equation modeling approach. *Psychol Methods*. 2014;19:211–29. doi: 10.1037/a0032968

73 Van den Noortgate W, López-López JA, Marín-Martínez F, *et al.* Three-level meta-analysis of dependent effect sizes. *Behav Res Methods*. 2013;45:576–94. doi: 10.3758/s13428-012-0261-6

74 Borenstein M, Cooper H, Hedges L, *et al.* Effect sizes for continuous data. *Handb Res Synth Meta-Anal*. 2009;2:221–35.

75 Weisz JR, Kuppens S, Ng MY, *et al.* What five decades of research tells us about the effects of youth psychological therapy: A multilevel meta-analysis and implications for science and practice. *Am Psychol*. 2017;72:79–117. doi: 10.1037/a0040360

76 Pustejovsky JE, Tipton E. Meta-analysis with Robust Variance Estimation: Expanding the Range of Working Models. *Prev Sci Off J Soc Prev Res*. 2022;23:425–38. doi: 10.1007/s11121-021-01246-3

77 Cheung MW-L. metaSEM: An R package for meta-analysis using structural equation modeling. *Front Psychol*. 2015;5:1521.

78 Core Team R. R: A language and environment for statistical computing [Software]. *R Found Stat Comput*. 2021.

79 Guest E, Zucchelli F, Costa B, *et al.* A systematic review of interventions aiming to promote positive body image in children and adolescents. *Body Image*. 2022;42:58–74. doi: 10.1016/j.bodyim.2022.04.009

80 Malinauskas R, Malinauskiene V. A meta-analysis of psychological interventions for Internet/smartphone addiction among adolescents. *J Behav Addict*. 2019;8:613–24.

81 Cowling M, Sim KN, Orlando J, *et al.* Untangling Digital Safety, literacy, and Wellbeing in School activities for 10 to 13 Year Old Students. *Educ Inf Technol*. 2025;30:941–58.

For peer review only - http://bmjopen.bmj.com/site/about/guidelines.xhtml

Supplementary Material

Supplement 1. *Example SCOPUS Search String.*

**Wellbeing Domain**	**Search Strategy**
Psychological/Subjective<br><br>Wellbeing:	TITLE-ABS-KEY ((adolescen* OR teen* OR student* OR pupil* OR youth OR "young pe*")<br><br>AND<br><br>("digital skill*" OR "digital safety" OR "online safety" OR "online resilience" OR "digital resilience" OR "digital competenc*" OR "internet safety" OR "social media literacy" OR "digital literacy" OR "media literacy" OR "information literacy" OR "AI literacy" OR misinformation OR "social media" OR "digital media" OR "social network*" OR online)<br><br>AND<br><br>("mental health" OR "mental illness" OR "mental disorder*" OR "psychological distress" OR wellbeing* OR "mental wellbeing" OR "life satisfaction" OR affect* OR "emotional wellbeing" OR "psychological wellbeing" OR resilience OR happiness OR optimism OR "personal growth" OR "self expression" OR identity OR suicid* OR "self-

For peer review only

	harm" OR depress* OR anxi* OR stress OR internali?* OR externali?* OR "self esteem" OR "self concept" OR "self worth" OR "body image" OR "eating disorder*" OR "disordered eating")<br><br>AND<br><br>("secondary school" OR "high school" OR "middle school" OR "post primary" OR "secondary education" OR classroom* OR "school-based" OR "whole school")<br><br>AND<br><br>(experiment* OR randomi?ed OR "randomi?ed controlled trial*" OR "randomi?ed control trial*" OR rct OR "cluster rct" OR "cluster randomi?ed*" OR "quasi-experiment*" OR evaluation OR pretest OR posttest OR "pre test" OR "post test" OR lesson* OR intervention* OR effectiveness)
Social Wellbeing	TITLE-ABS-KEY ((adolescen* OR teen* OR student* OR pupil* OR youth OR "young pe*") <br>AND

For peer review only - http://bmjopen.bmj.com/site/about/guidelines.xhtml

	("digital skill*" OR "digital safety" OR "online safety" OR "online resilience" OR "digital resilience" OR "digital competenc*" OR "internet safety" OR "social media literacy" OR "digital literacy" OR "media literacy" OR "information literacy" OR "AI literacy" OR misinformation OR "social media" OR "digital media" OR "social network*" OR online)<br><br>AND<br><br>(loneliness OR "social isolation" OR "social connect*" OR belonging OR friendship OR "social support" OR "peer rejection")<br><br>AND

For peer review only - http://bmjopen.bmj.com/site/about/guidelines.xhtml

	("secondary school" OR "high school" OR "middle school" OR "post primary" OR "secondary education" OR classroom* OR "school-based" OR "whole school")<br><br>AND<br><br>(experiment* OR randomi?ed OR "randomi?ed controlled trial*" OR "randomi?ed control trial*" OR rct OR "cluster rct" OR "cluster randomi?ed*" OR "quasi-experiment*" OR evaluation OR pretest OR posttest OR "pre test" OR "post test" OR lesson* OR intervention* OR effectiveness)
Physical Wellbeing	TITLE-ABS-KEY ((adolescen* OR teen* OR student* OR pupil* OR youth OR "young pe*") <br>AND

For peer review only - http://bmjopen.bmj.com/site/about/guidelines.xhtml

	("digital skill*" OR "digital safety" OR "online safety" OR "online resilience" OR "digital resilience" OR "digital competenc*" OR "internet safety" OR "social media literacy" OR "digital literacy" OR "media literacy" OR "information literacy" OR "AI literacy" OR misinformation OR "social media" OR "digital media" OR "social network*" OR online)<br><br>AND<br><br>("physical wellbeing" OR "physical well-being" OR sleep* OR "sedentary behav*" OR exercise OR "substance abuse" OR "substance use" OR "physical activ*" OR "health behav*" OR "sexual health")<br><br>AND<br><br>("secondary school" OR "high school" OR "middle school" OR "post primary" OR "secondary education" OR classroom* OR "school-based" OR "whole school")

For peer review only - http://bmjopen.bmj.com/site/about/guidelines.xhtml

	AND<br><br>(experiment* OR randomi?ed OR "randomi?ed controlled trial*" OR "randomi?ed control trial*" OR rct OR "cluster rct" OR "cluster randomi?ed*" OR "quasi-experiment*" OR evaluation OR pretest OR posttest OR "pre test" OR "post test" OR lesson* OR intervention* OR effectiveness)
Digital Wellbeing	TITLE-ABS-KEY ((adolescen* OR teen* OR student* OR pupil* OR youth OR "young pe*") <br>AND<br><br>(“digital skill*" OR "digital safety" OR "online safety" OR "online resilience" OR "digital resilience" OR "digital competenc*” OR "internet safety" OR "social media literacy" OR "digital literacy" OR "media literacy"

For peer review only - http://bmjopen.bmj.com/site/about/guidelines.xhtml

	OR "information literacy" OR "AI literacy" OR misinformation OR "social media" OR "digital media" OR "social network*" OR online)<br><br>AND<br><br>("digital well*" OR "online well*" OR cyberbull* OR bully* OR victimis* OR victimiz* OR harass* OR aggress* OR "online abuse" OR sext* OR groom* OR "harmful content" OR "violent content" OR "self-harm content" OR "hate speech" OR extremis* OR radicalis* OR radicaliz* OR "problematic internet*" OR "problematic social media*" OR "internet addiction" OR "social media addiction" OR "problematic smartphone*" OR "technology overuse" OR "gaming addiction" OR "problematic gaming" OR "online gambling" OR "screen time" OR "digital fatigue" OR "information overload" OR "fear of missing out" OR fomo OR pornograph* OR porn*)

For peer review only - http://bmjopen.bmj.com/site/about/guidelines.xhtml

	AND<br><br>("secondary school" OR "high school" OR "middle school" OR "post primary" OR "secondary education" OR classroom* OR "school-based" OR "whole school")<br><br>AND<br><br>(experiment* OR randomi?ed OR "randomi?ed controlled trial*" OR "randomi?ed control trial*" OR rct OR "cluster rct" OR "cluster randomi?ed*" OR "quasi-experiment*" OR evaluation OR pretest OR posttest OR "pre test" OR "post test" OR lesson* OR intervention* OR effectiveness)

For peer review only - http://bmjopen.bmj.com/site/about/guidelines.xhtml

Supplement 2. *Categorisation of Wellbeing Constructs for the Current Review**

**Wellbeing Domain**	**Direction of Indicator**	**Example & Definition**	**Example Representative Measures**
Psychological and Subjective Wellbeing	Positive Indicators	Life Satisfaction: Measures that assess satisfaction with life overall.	Satisfaction with Life Scale (Diener et al., 1985).
		Positive Affect: Measures that assess how much positive affect the individual experiences in their life.	PANAS–Positive Affect (Watson et al., 1988).
		Composite Wellbeing Measures which assess an individual's overall subjective wellbeing	Warwick Edinburgh Mental Wellbeing Scale (Tennant et al., 2007).

For peer review only - http://bmjopen.bmj.com/site/about/guidelines.xhtml

		Resilience<br><br>Measures which assess the ability to adapt and/or recover.	Adolescent Psychological Resilience Scale (APRS; Bulut et al., 2013).
		Optimism<br><br>Measures general expectations about positive outcomes.	Revised Life Orientation Test (LOT-R; Scheier et al., 1994).
		Quality of Life<br><br>Assesses overall satisfaction and fulfilment across life domains.	The Quality-of-Life Scale (QOLS; Burckhardt et al., 2003).
		Body Satisfaction<br><br>Measures contentment with various aspects of their body.	The Body Satisfaction Scale (Slade et al., 1990).
		Body Esteem<br><br>Measures positive self-evaluations of	The Body Esteem Scale (Franzoi & Shields, 1984).

For peer review only - http://bmjopen.bmj.com/site/about/guidelines.xhtml

		one's body and appearance.	
		Self Esteem<br><br>Measures an individual's overall sense of self-worth and value.	Rosenberg self-esteem scale (Rosenberg, 2011).
	Negative Indicators	DSM-V defined mental health outcomes (e.g., depression, anxiety, stress)<br><br>Measures which assess mental health outcomes as defined by the DSM-V.	Beck Depression Inventory (Beck, 1961).<br><br>State–Trait Anxiety Inventory (Spielberger, 1994)<br><br>Beck Anxiety Inventory (Beck et al., 1988).
		Negative Affect:<br><br>Measures that assess how much negative effect the individual	PANAS–Negative Affect (Watson et al., 1988).

For peer review only - http://bmjopen.bmj.com/site/about/guidelines.xhtml

		experiences in their life.	
		Body Dissatisfaction<br><br>Measures an individual's negative attitudes or feelings towards their own physical appearance.	Appearance evaluation subscale of the multidimensional body-self relations questionnaire (Cash, 2017).
Social Wellbeing	Positive Indicators	Social Connectedness<br><br>Measures perceived closeness and belonging in relationships and society.	The Social Connectedness scale (Lee & Robbins, 1995).
		Perceived Social Support<br><br>Measures the subjective feeling of having support from	Social Support Questionnaire (SSQ; Sarason et al., 1983).<br><br>The Child and Adolescent Social Support Scale.

For peer review only - http://bmjopen.bmj.com/site/about/guidelines.xhtml

		others. E.g. family, peers, and teachers.	(CASSS; Malecki et al., 2018).
	Negative Indicators	Loneliness<br><br>Measures subjective feelings of isolation and lack of social connection.	UCLA (Russell et al., 1978).
		Social Isolation<br><br>Measures lack of social networks and frequency of social interactions.	Lubben Social Network Scale (Lubben et al., 2006).
		Peer Rejection<br><br>Measures the degree to which an individual is disliked, excluded, or rejected by peers.	Social peer rejection measure (Lev-Wiesel et al., 2013).
		Cyberbullying Victimisation<br><br>Measures experiences of being targeted or harassed online.	Scale of Victimisation through the Cell Phone and Internet (Álvarez-García et al., 2017).

For peer review only - http://bmjopen.bmj.com/site/about/guidelines.xhtml

			Cyberbullying victimization (CBV) scale (Lee et al., 2017).
		Cyberbullying Perpetration<br><br>Measures engagement in online bullying behaviours, such as sending harmful messages, spreading rumours, or excluding others digitally.	Scale of Aggression through the Cell Phone and Internet (Álvarez-García et al., 2016)<br><br>Cyberbullying perpetration (CBP) scale (Lee et al., 2017)
Physical Wellbeing	Positive/Negative Indicators	Self-Reported Health Status<br><br>Measures perceived overall general health.	Self-rated health (SRH; Bombak, 2013).
		Sleep Quality<br><br>Measures satisfaction with sleep and restfulness.	Pittsburgh Sleep Quality Index (Buysse et al., 1989).
		Substance Use Behaviours	Risk Behaviour Index–5 items on tobacco, alcohol, and

For peer review only

		Measures risk behaviours related to tobacco, alcohol, and drug use.	drug use (Williams et al., 2000).
		Physical Activity<br><br>Measures frequency and intensity of exercise.	Physical Activity Questionnaire for Adolescents (PAQ-A; Kowalski et al., 1997).
	Negative Indicators	Perceived/Self-Reported Sleep Problems<br><br>Measures difficulties falling asleep, staying asleep, or feeling rested, based on self-report.	Adolescent Sleep Hygiene Scale (ASHS; LeBourgeois et al., 2005).
		Sedentary Behaviour<br><br>Measures time spent sitting or engaging in low-energy activities.	Sedentary Behavior Questionnaire (SBQ; Rosenberg et al., 2010).

For peer review only - http://bmjopen.bmj.com/site/about/guidelines.xhtml

	Positive	Health-Related Quality of Life<br><br>Measures the impact of various dimensions of health on quality of life.	SF-36 (Short Form 36 Health Survey; Ware & Sherbourne, 1992).
Digital-Specific Wellbeing	Negative	Social media addiction<br><br>Measures problematic or compulsive use of social media.	The Bergen Facebook Addiction Scale (BFAS) (Bowman & Clark-Gordon, 2019).
		Problematic social media use<br><br>Measures negative patterns of use which disrupt functioning.	Social Media Disorder scale (Boer et al., 2022).<br><br>Bergen Social Media Addiction Scale (BSMAS; Lin et al., 2017).
		Online gambling behaviour<br><br>Measures problem gambling and related	Adolescent Gambling Inventory's (CAGI) Problem Gambling Severity Index (PGSI; Tremblay et al., 2010).

For peer review only

For peer review only - http://bmjopen.bmj.com/site/about/guidelines.xhtml

		harms in online settings.	
		Online financial risk behaviours<br><br>Measures risky financial actions online and related consequences.	DOSPERT - Financial domain specific (Weber et al., 2002).
		Compulsive tech use<br><br>Measures excessive or uncontrolled use of digital devices or the internet leading to negative consequences/ functional impairment.	The Problematic and Risky Internet Use Screening Scale (PRIUSS; Jelenchick et al., 2014).

*Illustrative examples for the purposes of the protocol.

For peer review only

For peer review only - http://bmjopen.bmj.com/site/about/guidelines.xhtml

7 **Supplement 3.** *Categorisation of Media and Digital Literacy Outcomes for the Current*
8 *Review**

Domain	Definition	Example of Measures
Functional Skills	The skills/practical ability to access and use devices and digital technologies.	• Internet skills for school (ISFS; Kuhlemeier & Hemker, 2007).<br>• iKnow measure (internet knowledge), (Potosky, 2007).<br>• yDSI skills, content creation & production dimension (Helsper et al., 2021).
Critical Skills	The ability to evaluate, assess and analyse media in a critical way.	• Media Literacy Scale (Arke & Primack, 2009).<br>• MeHLit – Media Health Literacy Scale (Nazarnia et al., 2022).<br>• Media Attitudes Questionnaire-Realism Scepticism (Irving et al., 1998).<br>• Critical Processing of Beauty Images Scale (CPBI; Engeln-Maddox & Miller, 2008).<br>• The Critical Thinking about Media Messages (CTMM) scale, (McLean et al., 2016).

For peer review only - http://bmjopen.bmj.com/site/about/guidelines.xhtml

Creative Production	The ability to create, develop and distribute various forms of media/content.	• yDSI skills, content creation & production dimension (Helsper et al., 2021).

*Illustrative examples for the purposes of the protocol.

For peer review only

For peer review only - http://bmjopen.bmj.com/site/about/guidelines.xhtml

PRISMA-P (Preferred Reporting Items for Systematic review and Meta-Analysis Protocols) 2015 checklist: recommended items to address in a systematic review protocol*

Section and topic	Item No	Checklist item	Location where item is reported
**ADMINISTRATIVE INFORMATION**			
Title:			
Identification	1a	Identify the report as a protocol of a systematic review	Pg. 1
Update	1b	If the protocol is for an update of a previous systematic review, identify as such	n/a
Registration	2	If registered, provide the name of the registry (such as PROSPERO) and registration number	Pg. 9
Authors:			
Contact	3a	Provide name, institutional affiliation, e-mail address of all protocol authors; provide physical mailing address of corresponding author	Pg. 1
Contributions	3b	Describe contributions of protocol authors and identify the guarantor of the review	Pg.1
Amendments	4	If the protocol represents an amendment of a previously completed or published protocol, identify as such and list changes; otherwise, state plan for documenting important protocol amendments	n/a
Support:			
Sources	5a	Indicate sources of financial or other support for the review	Pg. 25
Sponsor	5b	Provide name for the review funder and/or sponsor	Pg. 25
Role of sponsor or funder	5c	Describe roles of funder(s), sponsor(s), and/or institution(s), if any, in developing the protocol	Pg. 25
**INTRODUCTION**			
Rationale	6	Describe the rationale for the review in the context of what is already known	Pg. 4 - 8
Objectives	7	Provide an explicit statement of the question(s) the review will address with reference to participants, interventions, comparators, and outcomes (PICO)	Pg. 8
**METHODS**			
Eligibility criteria	8	Specify the study characteristics (such as PICO, study design, setting, time frame) and report characteristics (such as years considered, language, publication status) to be used as criteria for eligibility for the review	Pg. 11 - 18
Information sources	9	Describe all intended information sources (such as electronic databases, contact with study authors, trial registers or other grey literature sources) with planned dates of coverage	Pg. 9
Search strategy	10	Present draft of search strategy to be used for at least one electronic database, including planned limits, such that it could be repeated	Supplements

For peer review only

Study records:			
Data management	11a	Describe the mechanism(s) that will be used to manage records and data throughout the review	Pg. 19
Selection process	11b	State the process that will be used for selecting studies (such as two independent reviewers) through each phase of the review (that is, screening, eligibility and inclusion in meta-analysis)	Pg. 19
Data collection process	11c	Describe planned method of extracting data from reports (such as piloting forms, done independently, in duplicate), any processes for obtaining and confirming data from investigators	Pg. 19 - 20
Data items	12	List and define all variables for which data will be sought (such as PICO items, funding sources), any pre-planned data assumptions and simplifications	Pg. 20 21
Outcomes and prioritization	13	List and define all outcomes for which data will be sought, including prioritization of main and additional outcomes, with rationale	Pg. 21
Risk of bias in individual studies	14	Describe anticipated methods for assessing risk of bias of individual studies, including whether this will be done at the outcome or study level, or both; state how this information will be used in data synthesis	Pg. 21
Data synthesis	15a	Describe criteria under which study data will be quantitatively synthesised	Pg. 22
	15b	If data are appropriate for quantitative synthesis, describe planned summary measures, methods of handling data and methods of combining data from studies, including any planned exploration of consistency (such as I^2, Kendall's τ)	Pg. 22 - 24
	15c	Describe any proposed additional analyses (such as sensitivity or subgroup analyses, meta-regression)	Pg. 23 - 24
	15d	If quantitative synthesis is not appropriate, describe the type of summary planned	n/a
Meta-bias(es)	16	Specify any planned assessment of meta-bias(es) (such as publication bias across studies, selective reporting within studies)	Pg. 21
Confidence in cumulative evidence	17	Describe how the strength of the body of evidence will be assessed (such as GRADE)	n/a

*** It is strongly recommended that this checklist be read in conjunction with the PRISMA-P Explanation and Elaboration (cite when available) for important clarification on the items. Amendments to a review protocol should be tracked and dated.** **The copyright for PRISMA-P (including checklist) is held by the PRISMA-P Group and is distributed under a Creative Commons Attribution Licence 4.0.**

From: Shamseer L, Moher D, Clarke M, Ghersi D, Liberati A, Petticrew M, Shekelle P, Stewart L, PRISMA-P Group. Preferred reporting items for systematic review and meta-analysis protocols (PRISMA-P) 2015: elaboration and explanation. BMJ. 2015 Jan 2;349(jan02 1):g7647.

For peer review only

BMJ Open

Wellbeing-focused Media Literacy Interventions in Secondary Schools: A Systematic Review and Meta-analysis Protocol

Journal:	*BMJ Open*
Manuscript ID	bmjopen-2026-119555.R2
Article Type:	Protocol
Date Submitted by the Author:	22-May-2026
Complete List of Authors:	Tibbs, Maria; University of York, Psychology in Education Research Centre, Department of Education.<br>Francis, Gill ; University of York, Psychology in Education Research Centre, Department of Education.<br>Mountain, Jennifer ; University of York, Psychology in Education Research Centre, Department of Education.<br>Polizzi, Gianfranco ; University of Birmingham, Department of Linguistics and Communication<br>Bell, Beth T; University of York, Psychology in Education Research Centre, Department of Education.
**Primary Subject Heading**:	Mental health
Secondary Subject Heading:	Paediatrics
Keywords:	Meta-Analysis, Systematic Review, MENTAL HEALTH, Schools, Social Media

SCHOLARONE™
MANUSCRIPTS

Wellbeing-focused Media Literacy Interventions in Secondary Schools: A Systematic Review and Meta-analysis Protocol

Maria Tibbs[1], Gill Francis[1], Jennifer Mountain[1], Gianfranco Polizzi[2], Beth T. Bell[1*].

[1] Psychology in Education Research Centre, Department of Education, University of York, United Kingdom.

[2] Department of Linguistics and Communication, University of Birmingham, Birmingham, United Kingdom.

***Corresponding author**

Email: Beth.bell@york.ac.uk.

Keywords: Adolescent wellbeing; school-based interventions; media literacy; digital literacy; digital wellbeing; meta-analysis; systematic review.

Wordcount: 4,744

Abstract

Introduction

Media and digital technologies shape adolescent development across interconnected online and offline contexts, offering opportunities for learning and connection while also exposing young people to risks that can affect wellbeing. Wellbeing-focused media literacy interventions aim to support young people in understanding and managing how media and technology shape their experiences and wellbeing. Schools are well placed to deliver such interventions at scale through curricular integration. However, existing interventions vary widely in focus, design, and evaluation quality, making it difficult to determine what works and why. This review synthesises school-based wellbeing-focused media literacy interventions and evaluates their effectiveness across wellbeing outcomes.

Methods and analysis

A range of interdisciplinary databases (ACM Digital Library, British Education Index, CINAHL, ERIC, MEDLINE, PsycINFO, Scopus, Web of Science), alongside targeted grey literature searches of internal evaluation reports and dissertations, were searched on 20th January 2026. Eligible studies will include school-based interventions for adolescents aged 11 to 18. Abstracts and full texts will be screened in Covidence, and relevant data extracted using an adapted TIDieR framework. A three-level random effects meta-analysis will estimate pooled effects across psychological, social, physical, and digital wellbeing domains. Narrative synthesis, informed by the adapted TIDieR framework, will explore intervention content, delivery, and theoretical foundations to identify mechanisms of change and sources of heterogeneity.

Ethics and dissemination

Ethical approval is not required for this systematic review, as it will only include published data. The review findings will be disseminated through a peer-reviewed publication and conference presentations.

Registration details

This protocol has been preregistered on the Open Science Framework Platform (OSF; **10.17605/OSF.IO/APM7F**) and PROSPERO (CRD420251269724)

Article Summary

- Uses a three level random effects meta-analysis that accounts for dependency among multiple effect sizes within studies, allowing more accurate synthesis of complex intervention outcomes.
- Comprehensive interdisciplinary search across psychology, education, public health and human computer interaction databases alongside grey literature sources to maximise coverage of relevant interventions.
- Integrates interventions targeting wellbeing outcomes associated with media and technology use including psychological, social, physical and digital wellbeing within a single media literacy framework, helping consolidate previously fragmented areas of research.
- Intervention characteristics will be systematically coded using an adapted TIDieR framework to identify intervention components and mechanisms of change.
- Substantial heterogeneity in intervention content, theoretical frameworks, delivery formats and outcome measures may still limit comparability across studies despite the use of a multilevel meta-analytic approach and use of subgroup analyses.

Introduction

Rates of mental health difficulties among adolescents have risen globally in recent decades [1–3], accompanied by declines in feelings of happiness, and positive affect [4–6]. Most mental health difficulties emerge before the age of 25 [7], and in the United Kingdom one in five adolescents were reported to have a probable mental health disorder in 2023 [8]. At the same time, there has been a rapid growth in youth digital technology use, with adolescents in the United Kingdom spending an average of four to six hours per day online [9]. While these trends have unfolded concurrently, existing evidence suggests that technologies are not inherently harmful to young people, but rather associations between digital technology use and mental health are complex and shaped by a wide range of social, developmental, and contextual factors.

For contemporary adolescents, social life, learning, and self-expression are increasingly shaped by media and digital technologies, including social media platforms such as YouTube, TikTok, Snapchat, and WhatsApp [10–12]. These technologies provide opportunities for connection, creativity, and community. However, they can also expose young people to cyberbullying, harmful or distressing content, reinforce sedentary behaviours, and promote upward comparison, all of which may undermine mental health and wellbeing [8,13–17]. Supporting young people to develop capable and informed engagement with media and technology is therefore a pressing public health and educational concern [18].

Defining Wellbeing

Wellbeing is commonly defined as "*how people feel and how they function, both on a personal and a social level and how they evaluate their lives as a whole*" [19, p.6], though its conceptualisation and measurement vary considerably across the literature [20,21]. It is widely recognised as a multidimensional construct, encompassing psychological, physical and social domains [22]. Psychological wellbeing includes two components; subjective

(hedonic) wellbeing, such as life satisfaction and affect balance [23,24] and psychological (eudaimonic) wellbeing, which focuses on qualities associated with flourishing, such as purpose and personal growth [25,26]. Physical wellbeing is frequently indexed through engagement in health-promoting behaviours (e.g., physical activity; [27] and avoidance of health-risk behaviours (e.g., smoking and substance use; [28]. Social wellbeing captures the extent to which individuals feel integrated into and supported by their social networks and communities, encompassing experiences of belonging, acceptance, and positive relationships [29]. Wellbeing, at both the personal and social level, can also be measured using more objective indicators, including physical/mental health status, educational attainment, socioeconomic security, and housing quality [30].

As young people's everyday lives have become deeply intertwined with digital technologies, scholars have increasingly argued that wellbeing frameworks must also incorporate a digital dimension. Digital wellbeing has been defined as the "*subjective individual experience of optimal balance between the benefits and drawbacks obtained from mobile connectivity*" [31, p.938]. Promoting wellbeing in contemporary society therefore requires approaches that attend to multiple, interrelated dimensions of wellbeing and equip young people with the knowledge and skills to engage with media and technology in ways that support personal and social flourishing in an increasingly digital world.

Media Literacy Education

Media literacy education offers one potential pathway for promoting well-being among adolescents. Media literacy refers to the ability to access, analyse, evaluate, and create media, with an emphasis on critically understanding media messages and their underlying intentions [32,33]. As media environments have become increasingly digitised, this framing has been extended to include digital literacy, applying the same core functional, critical, and

For peer review only - http://bmjopen.bmj.com/site/about/guidelines.xhtml

socioemotional competencies to online media, platforms, and technologies, alongside related literacies such as information and AI literacy [34,35]. In this framing, digital literacy is typically understood as a context-specific subfield of media literacy, that encompasses the same underlying competencies. These include functional competencies such as searching for information, creating content, and navigating platforms [36] and critical competencies, such as evaluating online content and understanding how platform infrastructures and algorithms shape what users encounter and produce [37–39].

Accordingly, in this review we use the term *media literacy interventions* as an umbrella to refer to interventions grounded in media literacy frameworks and traditions, including those applied to digital contexts (e.g., AI literacy, social media literacy) and commonly described as digital literacy interventions. There is growing recognition that media literacy includes an integral wellbeing dimension, involving the socioemotional knowledge and skills needed to navigate the psychological demands of modern-day media and digital technology and to understand how these experiences can affect wellbeing [40]. Emerging evidence suggests that these functional, critical and socioemotional skills are needed to help young people use technology in ways that maintain, enhance, support and protect wellbeing [41–44].

Wellbeing-focused Media Literacy Interventions

Schools have become a key setting for initiatives aimed at improving young people's media literacy in relation to wellbeing, as they reach learners during formative developmental periods, enable integration into curricula, and allow delivery at scale [45]. In the United Kingdom, improving media and digital skills is now a governmental priority [46], and education on some online harms is mandated through the Relationships, Sex and Health Education curriculum [47].

For peer review only - http://bmjopen.bmj.com/site/about/guidelines.xhtml

Increasingly, a diverse range of interventions aim to foster media literacy in ways that support youth wellbeing in both online and offline contexts. For example, interventions have been developed to promote positive body image by fostering media literacy in relation to harmful appearance-related messaging across media and technology, while also teaching psychological and behavioural coping strategies [48,49]. Interventions have also been developed to promote social wellbeing in online spaces by providing education related to how the design features and social affordances of online media environments may shape and sustain cyberbullying behaviours and social relationships [50,51]. While these interventions vary in terms of content and approach, a commonality is that all aim to *develop the knowledge and skills needed to foster wellbeing in relation to media and technology* through three interrelated sets of competencies:

(1) Critical knowledge and skills to analyse and evaluate media and technology.

(2) Functional knowledge and skills to navigate and operate media and technology in autonomous ways.

(3) Socio-emotional knowledge and skills to manage personal and social wellbeing in relation to media and technology.

In many cases, wellbeing is positioned as an assumed or downstream outcome arising from the development and application of these competencies, rather than as an explicit focus of intervention theory or evaluation. We refer to this diverse group of approaches as *wellbeing-focused media literacy interventions*.

Despite growing recognition of their educational and public health relevance [52], little is known about which wellbeing-focused media literacy interventions work, for whom, and why. Existing reviews have largely examined intervention effectiveness within specific domains, such as cyberbullying [53] and body image [54], even though interventions often share elements that promote critical awareness, self-reflection, and behavioural change [55]. This fragmentation prevents understanding of shared mechanisms or "active ingredients" that drive positive outcomes across intervention types and limits understanding of what approaches are most effective or transferable to real-world educational settings.

Objectives

Accordingly, this systematic review aims to identify classroom-based wellbeing-focused media literacy interventions that are effective among adolescents aged 11 to 18. Furthermore, it aims to examine the components and characteristics that underpin effectiveness. Specifically, it addresses the following questions:

1. Do school-based wellbeing-focused media literacy interventions improve youth wellbeing outcomes (psychological/subjective, social, physical, and digital)?
2. What active ingredients (components & characteristics) underpin effective school-based well-being focused media literacy interventions?

Materials and Method

This study will conduct a systematic review and meta-analysis examining the effectiveness of eligible interventions. The review will include experimental and quasi-experimental studies, including dissertations employing these designs, provided they meet the predefined eligibility criteria. Where sufficient comparable data are available, quantitative findings from eligible studies will be synthesised using meta-analysis to estimate the effects of the interventions under investigation.

Protocol and Registration

The current protocol is aligned with the Preferred Reporting Items for Systematic Reviews and Meta-Analyses Protocols (PRISMA-P; [56], see supplementary materials. The subsequent systematic review and meta-analysis will be reported following the Preferred Reporting Items for Systematic Reviews and Meta-Analyses (PRIMSA; [57].

This protocol has been preregistered on the Open Science Framework Platform (OSF; **10.17605/OSF.IO/APM7F**) and PROSPERO (CRD420251269724). Any significant amendments made to the protocol will be documented and reported in the final systematic review manuscript. Database searches have been conducted, and title and abstract screening is currently ongoing, with completion anticipated by November 2026.

Information Sources and Search Strategy

The systematic search included both peer-reviewed and grey literature published in English, with no geographic restrictions. Peer-reviewed studies were identified through interdisciplinary databases to ensure comprehensive coverage across education, psychology, public health and human computer interaction: ACM Digital Library, British Education Index, CINAHL, ERIC (Education Resources Information Centre), MEDLINE, PsycINFO, Scopus, Web of Science. Grey literature included doctoral theses and publicly available

For peer review only - http://bmjopen.bmj.com/site/about/guidelines.xhtml

internal reports evaluating interventions with randomised designs, as these sources can provide valuable, otherwise unpublished evidence subjected to some level of independent scrutiny. Doctoral theses were be identified through ProQuest Dissertations and Theses Global. All database searches were conducted on 20th January 2026. In addition to these interdisciplinary databases, websites of reputable media literacy and/or educational evaluation organisation websites (e.g., Education Endowment Foundation, Internet Matters, PSHE Association) will be searched for relevant internal evaluation reports. The rationale for including each database is presented in **Table 1**. To ensure all relevant articles will be captured, a process of forward reference and backward citation checking using *citationchaser* [58] will be conducted on eligible full texts.

The search strategy was developed in collaboration with a library specialist at the University of York and through examination of previous systematic reviews in the areas of digital literacy, media literacy, and wellbeing. Search terms focused on the core domains under investigation: the population of interest, characteristics of the intervention, relevant outcomes, the setting within which interventions are implemented, and study design, as well as MeSH terms or subject areas relevant to each database where applicable. Considering the breadth of potential wellbeing outcomes, the search strategy was conducted separately for each of the four wellbeing domains (psychological/subjective, social, physical, and digital) to ensure sufficient and relevant retrieval of studies. See **Supplementary Material 1** for an example SCOPUS search strategy.

Table 1.

Rationale for bibliographic databases

**Database**	**Rationale**

ERIC (Education Resources Information Centre)	Captures literature focused on education and learning contexts.
British Education Index	Covers educational literature focused within the British context, that may not be captured within ERIC.
Web of Science	Broad, multidisciplinary database indexing a wide range of high-impact peer-reviewed research.
PsycINFO	Specialises in psychology and related behavioural sciences.
CINAHL	Covers nursing, allied health, and applied healthcare research.
MEDLINE	Biomedical and public health database; includes extensive coverage of health interventions and uses MeSH terms, enhancing search precision.
SCOPUS	Indexes a broad range of journals across health sciences, technology, and education.
ACM Digital Library	Houses studies from human–computer interaction, a field that often contributes to research on digital literacy and technology use.
ProQuest Dissertations and Theses Global	Grey literature database that houses doctoral theses.

Eligibility Criteria

To be included in the current systematic review, all studies, including those identified across the grey literature, will need to meet the pre-defined inclusion and exclusion criteria as

For peer review only - http://bmjopen.bmj.com/site/about/guidelines.xhtml

informed by the Population, Intervention, Comparison, Outcome, Study Design (PICOS) model. For a summary of the inclusion criteria, **see Table 2.**

Population

To be included in this review, studies must focus on young people engaged in formal second level education aged 11- 18. This age range was selected to capture developmental stages typically corresponding to secondary-level schooling across international education systems, while excluding primary and post-secondary contexts.

Intervention

Eligible interventions must include a media literacy component, defined as educational content focused on developing adolescents' ability to access, analyse, evaluate, and create media, with an emphasis on critically understanding media messages and their underlying intentions [32,33]. Media or technology must be an active target of the intervention, rather than solely the mode of delivery.

Interventions may contain one or more of the following media literacy components, which reflect different ways in which wellbeing-focused media literacy is operationalised in practice:

1. **Critical:** The knowledge and skills needed to critically analyse and evaluate media and technology through a wellbeing lens (e.g. understanding persuasive intent, platform design, algorithms, or how media features shape experiences relevant to wellbeing).
2. **Functional:** The knowledge and skills needed to navigate and operate media and technology in ways that may support wellbeing, including modifying engagement,

using safety and support features, and enacting boundaries or help-seeking behaviours within digital environments.

3. **Socio-emotional:** The knowledge and skills needed to recognise and reflect on emotional and social responses to media and technology, and to evaluate when media engagement supports or undermines wellbeing.

Comparators

Studies will be included if they evaluate the intervention against either a passive control condition (e.g., no intervention, usual care, or waitlist/delayed care) or an active control condition (e.g., an alternative intervention or the same intervention delivered at a different time point). Studies that do not include a comparison or control group, such as pre–post, uncontrolled, or purely observational designs, will be excluded.

Outcomes

The primary outcome is wellbeing, which we define broadly as "*how people feel and how they function, both on a personal and a social level, and how they evaluate their lives as a whole*" [19, p.6]. To structure our analysis, we draw on the World Health Organisation's three-domain model of wellbeing, which includes psychological, social, and physical dimensions [22]. We extend this framework by adding a fourth domain: digital wellbeing. This expanded model allows us to capture the full spectrum of experiences and risks associated with young people's digital lives. For the purposes of this review, we define these four domains as follows:

1. ***Psychological/Subjective wellbeing*** reflects individuals' evaluations and lived experiences of their own lives, including their sense of life satisfaction, experiences of positive and negative emotions, self-concept, the absence of mental health difficulties, and overall capacity to flourish [59,60].

For peer review only - http://bmjopen.bmj.com/site/about/guidelines.xhtml

2. ***Social wellbeing*** refers to the extent to which individuals feel integrated into and supported by their social networks and communities [29]. This can include concepts such as belonging, friendship quality, as well as experiences of social isolation and loneliness.
3. ***Physical wellbeing*** can be described as the ability to maintain bodily health and energy through balanced behaviours such as exercise, diet, sleep, sexual health, and avoidance of harmful habits [61,62].
4. ***Digital wellbeing*** refers to individuals' ability to engage with digital technologies in ways that support wellbeing, including feeling safe, in control, and supported in online environments, as well as minimising exposure to digital risks such as harmful content, excessive use, or negative social interactions.

Secondary outcomes include indicators of digital, media, and information literacy, reflecting young people's knowledge, skills, and critical competencies in navigating online environments. For detail on the categorisation and representative measures, see **Supplementary Material 2 and 3.**

Study Design

This review will include English language, peer-reviewed experimental and quasi-experimental studies with a comparator or control group, including randomised controlled trials (cluster and individual) and non-randomised comparison designs (e.g. matched or non-equivalent control groups). Eligible studies must provide a quantitative comparison between intervention and control conditions to assess effectiveness or efficacy.

Timeframe

The search period was set from 2000 to the present date to capture developments in digital literacy during a transitional period of conceptual overlap with traditional media literacy,

when technologies were beginning to be integrated into media literacy education and regulation [63]. These developments are exemplified by the enactment of international policies such as the U.S. Children's Internet Protection Act (2000), the UK Communications Act (2003), and the EU Action Plan for a Safer Internet (1999-2004), which increasingly framed media literacy around online safety, responsible use, and digital participation alongside traditional media.

For peer review only - http://bmjopen.bmj.com/site/about/guidelines.xhtml

Table 2. *Inclusion/Exclusion Criteria*

	**Inclusion**	**Exclusion**
Population	Young people aged 11-18 attending post-primary, secondary, or high school.	Young people not attending post-primary, secondary, or high school.<br>Young people aged <11 or >18.
Intervention	• School or classroom-based media literacy interventions that include education on the knowledge and skills needed to foster wellbeing in relation to media and technology including critical, functional and socio-emotional.	Interventions that:<br>• Are not conducted within education settings.<br>• Do not target students' knowledge and skills related to media and technology.
Comparator	Studies will be included if they compare the intervention with either a passive control (e.g., no intervention, usual care, or waitlist/delayed care) or active control (e.g., an alternative intervention or the same intervention delivered at a different time point).	Studies without a comparison or control group (e.g., pre–post, uncontrolled, or observational designs) will be excluded.

For peer review only - http://bmjopen.bmj.com/site/about/guidelines.xhtml

Outcome	Studies will be included if their primary or secondary outcomes relate to either wellbeing and/or digital/media/information literacy.	• Studies will be excluded if they do not assess wellbeing as an outcome
Study Design	Studies will be included if they:<br>• Employ experimental designs with a comparator group, randomised controlled trials (including cluster-RCTs) and non-randomised comparison group designs (e.g. quasi-experiments using matched control or non-equivalent control group).<br>• Include a quantitative comparison between intervention and control or comparison conditions to evaluate effectiveness or efficacy.	Studies will be excluded if they:<br>• Do not include a comparator group (e.g. single-group pre–post, purely observational, or cross-sectional studies).<br>• Employ qualitative designs.<br>• Are systematic reviews and meta-analyses.<br>• Are intervention protocols without published findings available at the time of synthesis.
Timeframe	2000 to present.	Studies published before the year 2000.

For peer review only - http://bmjopen.bmj.com/site/about/guidelines.xhtml

Data Management

Search results will be exported to Zotero reference managers and duplicates will be removed. Covidence systematic review software will be used to screen the abstracts and full texts of identified studies. Microsoft Excel will be used to extract relevant study data and to conduct quality appraisals of all included studies.

Selection of Studies and Data Extraction

Following the removal of duplicates, studies will be screened in two stages using Covidence. First, titles and abstracts will be assessed against the inclusion and exclusion criteria. Full texts of potentially relevant studies will then be retrieved and reviewed for eligibility. Four reviewers experienced in systematic review methodologies will contribute to the data screening and extraction process. At each stage studies will be double screened, and discrepancies will be resolved through a joint discussion by the two reviewers, and the opinion of a third reviewer will be obtained if agreement is not reached. Inter-rater reliability (e.g., Cohen's κ) will be calculated to assess agreement between the two reviewers at title and abstract screening as well as full-text screening.

Similarly, two reviewers will duplicate data extraction and coding to ensure accuracy and consistency. One reviewer will extract, and code all included studies, while the remaining three reviewers will each extract and code a proportion of the studies to enable cross-checking and verification. Extracted data will be compared for accuracy and completeness, with any discrepancies resolved through discussion and arbitration by a fourth author where necessary.

A pre-piloted data extraction form will be used to capture bibliographic details, sample characteristics, intervention and comparison conditions, and quantitative outcomes from each included study. The form will capture:

1. **Key study characteristics:** including author details, study date, country, study aims and objectives.

2. **Participant and School characteristics:** including sample size and composition (e.g., gender distribution, mean age, type of school [public/private], and contextual factors such as rural/urban setting of the school).

3. **Intervention characteristics:** We will record intervention characteristics using an adapted version of the Template for Intervention Description and Replication (TIDieR) framework [64]. Using this, we will extract details on:

   a. The rationale, goal, and theoretical framing of the intervention.

   b. What is delivered (specific activities, and intervention components). This will be extended to capture which wellbeing-focused media literacy components are targeted. These components include: (1) critical knowledge and skills; (2) functional knowledge and skills; and (3) socio-emotional knowledge and skills. We acknowledge that these domains are interrelated and will not mutually exclusive. The coding framework will therefore allow for dual or multiple categorisations where components span domains.

   c. Who the intervention is delivered by (e.g., teachers, trained facilitators, peers).

   d. How the intervention was delivered (in-class online modules, group activities, presentations). This will be extended to extract details on mechanisms through which these interventions are theorised to achieve their outcomes. Specifically, we will code for teaching strategies and pedagogical approaches (e.g., reflection, discussion, experiential learning) and behaviour change techniques (BCT's; [65] used to enact behaviour change.

For peer review only - http://bmjopen.bmj.com/site/about/guidelines.xhtml

e. Where the intervention was delivered (e.g., within subject specific classes, after school, during pastoral care).

f. The frequency and duration of the intervention session(s).

g. Evidence of any modifications during the process of intervention implementation.

h. Details on adherence to any existing protocols.

i. Details of any adaptations or tailoring for different contexts.

4. **Outcomes:** including outcome domains, measures used, time points of assessment (e.g., pre-, post-, follow-up), directionality of wellbeing outcomes (positive or negative), and available relevant quantitative data (means, related effect sizes, and associated variance estimates) for meta-analytic calculations.

Quality Appraisal and Certainty of Evidence

To examine risk of bias across studies (including those identified through grey literature searching), the Cochrane Risk of Bias Tool (RoB2; [66] will be used. This tool evaluates the risk of bias by assigning studies a quality rating of low, high, or unclear across several domains of bias. Studies deemed low quality will be retained for both the systematic review and meta-analysis. However, in line with recent meta-analyses of school-based interventions [67,68], sensitivity analyses will be conducted excluding low-quality studies to examine their influence on the magnitude and robustness of the overall effect. Funnel plots will be used to assess potential publication bias, and Egger's test will be employed to statistically evaluate the presence of small-study effects.

For peer review only - http://bmjopen.bmj.com/site/about/guidelines.xhtml

Data Synthesis

Narrative Synthesis

Extracted data will be tabulated and narratively synthesised following Popay et al's. [69] guidance on narrative synthesis. Firstly, study, participant, and intervention characteristics will be summarised descriptively using the adapted TIDieR framework. Second, interventions will be compared according to their wellbeing-focused media literacy components (knowledge/understanding and skills) and the mechanisms of change identified (teaching strategies, pedagogies, and behaviour change techniques). Finally, the synthesis will explore how variations in these components and mechanisms related to wellbeing outcomes across domains (psychological/subjective, social, physical, digital). Using this approach, the synthesis will provide an overview of patterns in intervention content (what) and the mechanisms to enact behaviour change (how), providing a working theory of how the intervention works to achieve its outcomes [70].

Meta-analysis

All studies meeting the inclusion criteria will be included in the systematic review; however, only studies reporting sufficient quantitative data on wellbeing outcomes (e.g., means and standard deviations, change scores, or effect estimates with variance) will be eligible for inclusion in the meta-analysis. Studies lacking extractable data or reporting outcomes that cannot be meaningfully standardised will be synthesised narratively. Where outcomes are reported on continuous scales, effect sizes are expected to be calculated as standardised mean differences (Hedges' g), which is commonly used in meta-analyses of school-based interventions to accommodate heterogeneous measures and small sample sizes. Effect sizes will be interpreted in relation to those reported in prior meta-analyses of school-based interventions, with emphasis on robustness and practical relevance rather than fixed magnitude thresholds.

For peer review only - http://bmjopen.bmj.com/site/about/guidelines.xhtml

We will perform a meta-analysis using an intention-to-treat approach, as opposed to a per-protocol approach, to minimise post-randomisation bias and overestimation of effects [71]. This means that all participants originally allocated to intervention and control groups will be analysed according to their initial assignment, regardless of adherence or dropout, ensuring a more conservative and unbiased estimate of effect. To account for probable correlation and dependence between multiple effect sizes reported within individual studies, we will adopt a three-level random-effects meta-analysis to estimate pooled effects. A three-level random-effects meta-analysis partitions variance into three components:

1) Sampling variance associated each observed effect size (sampling error within studies).
2) Variance between effect sizes within the same study
3) Variance between study effects.

In this analysis, effect sizes are nested within studies, and multiple outcomes reported by the same study are modelled as correlated at level two. This approach allows the inclusion of several non-independent outcomes per study while appropriately accounting for their clustering [72,73].

To assess what proportion of the observed variance reflects variance in true effects, the I^2 statistic will be calculated, with cut-offs of 25, 50, and 75% denoting low, moderate, and high levels of heterogeneity. 95% confidence intervals and for the I^2 values will be calculated using the method outlined by Borenstein [74]. Finally, to explore sources of heterogeneity, we will conduct moderator analyses. Moderators will include study design characteristics (e.g., passive versus active control conditions, publication year), intervention characteristics (e.g., mode of delivery, number and duration of modules or lessons), and participant characteristics (e.g., gender composition). In addition, outcome domains (psychological/subjective, social, physical, digital) will be entered as categorical moderators

For peer review only - http://bmjopen.bmj.com/site/about/guidelines.xhtml

alongside study-level characteristics such as intervention design and participant details. This will allow us to formally test whether effects differ across outcome domains, as well as to examine potential sources of between-study heterogeneity. Following guidance from [75], analyses with categorical moderators will only be conducted if each category contains at least five cases.

As a robustness check, will fit a correlated-hierarchical effects model (CHE), which combines a hierarchical variance structure with robust variance estimation to permit inference without requiring known within-study correlations [76]. This will be accompanied by further sensitivity analyses using examination of the potential impact of outliers and risk of bias. Where substantial or unforeseen heterogeneity is identified, and where sufficient data are available, we may additionally conduct separate three-level or standard meta-analyses within wellbeing domains (e.g., psychological/subjective, social, physical, digital) to improve conceptual and methodological comparability of pooled estimates. Importantly, meta-analytic decisions will be guided by theoretical coherence, measurement comparability, and the distribution of available data within each domain. Where measures within a domain are conceptually or operationally heterogeneous, outcomes that cannot be meaningfully compared will be synthesised narratively rather than pooled.

Statistical analyses will be conducted using the *metaSEM* package [77] in R statistical software [78].

Ethics and Dissemination

Ethical approval is not required for this systematic review, as it will only include published data. The review findings will be disseminated through a peer-reviewed publication and conference presentations.

Public and Patient Involvement (PPI)

For peer review only - http://bmjopen.bmj.com/site/about/guidelines.xhtml

The current protocol has been presented and reviewed by $N = 10$ key stakeholders including as representatives from charities and advocacy groups, education specialists, methodological experts, and academic researchers as well as $N = 2$ young people.

Deviations from the Protocol

Any deviations from the registered protocol will be updated and recorded on the OSF and Prospero websites.

Strengths and Limitations

This review has several methodological and conceptual strengths. The protocol is preregistered on the Open Science Framework and PROSPERO and follows PRISMA-P guidance, supporting reproducibility and methodological rigour. The search strategy spans multiple interdisciplinary databases across education, psychology, public health, and human–computer interaction, alongside grey literature sources, increasing the likelihood of identifying a comprehensive body of relevant evidence. Conceptually, the review integrates interventions targeting wellbeing outcomes associated with media and technology use including psychological, social, physical, and digital wellbeing within a single media literacy framework. This approach helps consolidate previously fragmented areas of research. In addition, intervention characteristics will be systematically coded using an adapted Template for Intervention Description and Replication (TIDieR) framework, enabling the identification of active components and mechanisms that may underpin effective wellbeing-focused media literacy interventions.

However, some limitations should be acknowledged. Considerable heterogeneity is also anticipated in intervention content, theoretical foundations, delivery formats, and outcome measures across studies. Although this heterogeneity will be explicitly accounted for using a three-level random-effects meta-analytic model that accommodates dependency among

For peer review only - http://bmjopen.bmj.com/site/about/guidelines.xhtml

multiple effect sizes within studies, variation in intervention design and measurement may still limit comparability and complicate interpretation of pooled estimates. Finally, the strength of the conclusions will depend on the methodological quality and reporting practices of the included primary studies.

Conflicts of Interest

The authors declare no conflicts of interest.

Acknowledgements

The authors would like to thank members of both the steering committee and the youth advisory board for their invaluable contributions to the drafting of this manuscript.

Funding

This project has been funded by the Nuffield Foundation (Grant Number: EDO /FR-000025035), but the views expressed are those of the authors and not necessarily the Foundation. Visit nuffieldfoundation.org

Author Contributions

- **MT:** Conceptualisation, Methodology, Formal analysis plan, Investigation, Writing – original draft, responsible for the overall content as guarantor.
- **GF:** Conceptualisation, Methodology, Validation, Writing – review & editing.
- **JM:** Investigation, Data curation, Writing – review & editing.
- **GP:** Conceptualisation, Writing – review & editing.
- **BB:** Conceptualisation, Funding acquisition, Supervision, Writing – review & editing.

References

1 Cybulski L, Ashcroft DM, Carr MJ, *et al.* Temporal trends in annual incidence rates for psychiatric disorders and self-harm among children and adolescents in the UK, 2003–2018. *BMC Psychiatry*. 2021;21:229. doi: 10.1186/s12888-021-03235-w

2 Fitzgerald A, Mahon C, Shevlin M, *et al.* Exploring changing trends in depression and anxiety among adolescents from 2012 to 2019: Insights from My World repeated cross-sectional surveys. *Early Interv Psychiatry*. 2025;19:e13562. doi: 10.1111/eip.13562

3 Mojtabai R, Olfson M. National Trends in Mental Health Care for US Adolescents. *JAMA Psychiatry*. 2020;77:703–14. doi: 10.1001/jamapsychiatry.2020.0279

4 Blanchflower DG, Bryson A, Xu X. The declining mental health of the young and the global disappearance of the unhappiness hump shape in age. *PLOS ONE*. 2025;20:e0327858. doi: 10.1371/journal.pone.0327858

5 Dumuid D, Singh B, Brinsley J, *et al.* Trends in Well-Being Among Youth in Australia, 2017-2022. *JAMA Netw Open*. 2023;6:e2330098. doi: 10.1001/jamanetworkopen.2023.30098

6 Marquez J, Long E. A Global Decline in Adolescents' Subjective Well-Being: a Comparative Study Exploring Patterns of Change in the Life Satisfaction of 15-Year-Old Students in 46 Countries. *Child Indic Res*. 2021;14:1251–92. doi: 10.1007/s12187-020-09788-8

7 Solmi M, Radua J, Olivola M, *et al.* Age at onset of mental disorders worldwide: large-scale meta-analysis of 192 epidemiological studies. *Mol Psychiatry*. 2022;27:281–95.

8 NHS Digital. Mental Health of Children and Young People in England, 2023 - wave 4 follow up to the 2017 survey. 2023.

For peer review only - http://bmjopen.bmj.com/site/about/guidelines.xhtml

9 Ofcom. Online Nation Report. 2023. https://www.ofcom.org.uk/siteassets/resources/documents/research-and-data/online-research/online-nation/2023/online-nation-2023-report.pdf?v=368355

10 Charmaraman L, Hodes R, Richer AM. Young sexual minority adolescent experiences of self-expression and isolation on social media: cross-sectional survey study. *JMIR Ment Health*. 2021;8:e26207. doi:10.2196/26207

11 Goodyear VA, Armour KM. Young People's health-related learning through social media: What do teachers need to know? *Teach Teach Educ*. 2021. doi: 10.1016/j.tate.2021.103340

12 Mittmann G, Woodcock K, Dörfler S, *et al.* "TikTok Is My Life and Snapchat Is My Ventricle": A Mixed-Methods Study on the Role of Online Communication Tools for Friendships in Early Adolescents. *J Early Adolesc*. 2022;42:172–203. doi: 10.1177/02724316211020368

13 Al-Jbouri E, Volk AA, Spadafora N, *et al.* Friends, followers, peers, and posts: adolescents' in-person and online friendship networks and social media use influences on friendship closeness via the importance of technology for social connection. *Front Dev Psychol*. 2024;2:1419756. https://doi.org/10.3389/fdpys.2024.1419756

14 Caba-Machado V, Mcilroy D, Padilla-Adamuz FM. Night-time use of electronic devices, fear of missing out, sleep difficulties, anxiety, and well-being in UK and Spain: a cross-cultural comparison. *Curr Psychol*. 2024;43:21134–45. https://doi.org/10.1007/s12144-024-05934-5

15 Engberg E, Leppänen MH, Sarkkola C, *et al.* Physical Activity Among Preadolescents Modifies the Long-Term Association Between Sedentary Time Spent Using Digital Media and the Increased Risk of Being Overweight. Published Online First: 29 July 2021. doi: 10.1123/jpah.2021-0163

16 Peppler K, Dahn M. Social Media and Creativity. *The Routledge International Handbook of Children, Adolescents, and Media*. Routledge 2022.

17 Zheng Q, Yang W, Li T, *et al.* Examining the Pathways to Social Media FOMO: Mediation Effects of Passive Engagement and Upward Social Comparisons. *Int J Human–Computer Interact*. 2025;1–8. doi: 10.1080/10447318.2025.2499156

18 Büchi M. Digital well-being theory and research. *New Media Soc*. 2024;26:172–89. doi: 10.1177/14614448211056851

19 Michaelson J, Mahony S, Schifferes J. Measuring wellbeing: A guide for practitioners. *New Econ Found Lond*. 2012.

20 Dodge R, Daly A, Huyton J, *et al.* The challenge of defining wellbeing. Intnl. J. Wellbeing 2, 222–235. 2012. doi:10.5502/ijw.v2i3.4

21 Hone L, Schofield G, Jarden A. Conceptualizations of wellbeing: Insights from a prototype analysis on New Zealand workers. *N Z J Hum Resour Manag*. 2015;15:97–118.

For peer review only - http://bmjopen.bmj.com/site/about/guidelines.xhtml

22 World Health Organization. Summary reports on proceedings minutes and final acts of the international health conference held in New York from 19 June to 22 July 1946. *N Y WHO*. 1948;35.

23 Diener E, Suh EM, Lucas RE, *et al.* Subjective well-being: three decades of progress. *Psychol Bull*. 1999;125:276. https://doi.org/10.1037/0033-2909.125.2.276

24 Diener E, Ryan K. Subjective well-being: A general overview. *South Afr J Psychol*. 2009;39:391–406. https://doi.org/10.1177/008124630903900402

25 Ryan RM, Deci EL. On Happiness and Human Potentials: A Review of Research on Hedonic and Eudaimonic Well-Being. *Annu Rev Psychol*. 2001;52:141–66. doi: 10.1146/annurev.psych.52.1.141. DOI: 10.1146/annurev.psych.52.1.141

26 Ryff CD, Singer BH. Know Thyself and Become What You Are: A Eudaimonic Approach to Psychological Well-Being. *J Happiness Stud*. 2008;9:13–39. doi: 10.1007/s10902-006-9019-0

27 Hennessey A, MacQuarrie S, Petersen KJ. Exploring physical, subjective and psychological wellbeing profile membership in adolescents: a latent profile analysis. *BMC Psychol*. 2024;12:720. https://doi.org/10.1186/s40359-024-02196-5

28 Dittmar H, Bond R, Hurst M, *et al.* The relationship between materialism and personal well-being: A meta-analysis. *J Pers Soc Psychol*. 2014;107:879–924. doi: 10.1037/a0037409

29 Keyes CLM. Social well-being. *Soc Psychol Q*. 1998;121–40. https://doi.org/10.2307/2787065

30 Organisation for Economic Co-operation and Development Secretary-General, OECD iLibrary. How's life?: measuring well-being. Paris 2011.

31 Vanden Abeele MMP. Digital Wellbeing as a Dynamic Construct. *Commun Theory*. 2021;31:932–55. doi: 10.1093/ct/qtaa024

32 Aufderheide P. Media Literacy: From a Report of the National Leadership Conference on Media Literacy. *Media Literacy Around the World*. Routledge 1997.

33 Hobbs R. Digital and Media Literacy: A Plan of Action. A White Paper on the Digital and Media Literacy Recommendations of the Knight Commission on the Information Needs of Communities in a Democracy. Aspen Institute 2010.

34 Leaning M. An approach to digital literacy through the integration of media and information literacy. *Media Commun*. 2019;7:4–13. https://doi.org/10.17645/mac.v7i2.1931

35 Wuyckens G, Landry N, Fastrez P. Untangling media literacy, information literacy, and digital literacy: A systematic meta-review of core concepts in media education. *J Media Lit Educ*. 2022;14:168–82. doi: https://doi.org/10.23860/JMLE-2022-14-1-12

For peer review only - http://bmjopen.bmj.com/site/about/guidelines.xhtml

36 Van Deursen AJ, Helsper EJ, Eynon R. Development and validation of the Internet Skills Scale (ISS). *Inf Commun Soc*. 2016;19:804–23. http://dx.doi.org/10.1080/1369118X.2015.1078834

37 Buckingham D. Digital Media Literacies: rethinking media education in the age of the Internet. *Res Comp Int Educ*. 2007;2:43–55.

38 Dezuanni M. Minecraft and children's digital making: implications for media literacy education. *Learn Media Technol*. 2018;43:236–49. DOI: 10.1080/17439884.2018.1472607

39 Polizzi G. Internet users' utopian/dystopian imaginaries of society in the digital age: Theorizing critical digital literacy and civic engagement. *New Media Soc*. 2023;25:1205–26. https://doi.org/10.1177/14614448211018609

40 Tuamsuk K, Subramaniam M. The current state and influential factors in the development of digital literacy in Thailand's higher education. *Inf Learn Sci*. 2017;118:235–51. doi: 10.1108/ILS-11-2016-0076

41 Gordon CS, Ferber K, Notley T, *et al.* The relationship between media literacy and well-being: A systematic review and meta-analysis. *Educ Res Rev*. 2025;49:100731. doi: 10.1016/j.edurev.2025.100731

42 Kalmus V, Tambaum T, Abuladze L. Use of digital tools, digital skills and mental well-being. 2023:235–50.

43 Livingstone S, Mascheroni G, Stoilova M. The outcomes of gaining digital skills for young people's lives and wellbeing: A systematic evidence review. *New Media Soc*. 2023;25:1176–202. https://doi.org/10.1177/146144482110431

44 Vissenberg J, De Coninck D, Mascheroni G, *et al.* Digital Skills and Digital Knowledge as Buffers Against Online Mis/Disinformation? Findings from a Survey Study Among Young People in Europe. *Soc Media Soc*. 2023;9:20563051231207859. doi: 10.1177/20563051231207859

45 Carta MG, Di Fiandra T, Rampazzo L, *et al.* An overview of international literature on school interventions to promote mental health and well-being in children and adolescents. *Clin Pract Epidemiol Ment Health CP EMH*. 2015;11:16. DOI: 10.2174/1745017901511010016

46 DCMS. Department for Digital, Culture, Media and Sport: Annual Report and Accounts. 2022.

47 Department for Education. Teaching Online Safety in Schools. 2023.

48 Bell BT, Taylor C, Paddock D, *et al.* Digital Bodies: A controlled evaluation of a brief classroom-based intervention for reducing negative body image among adolescents in the digital age. *Br J Educ Psychol*. 2022;92:280–98. DOI: 10.1111/bjep.12449

For peer review only - http://bmjopen.bmj.com/site/about/guidelines.xhtml

49 Gordon CS, Jarman HK, Rodgers RF, *et al.* Outcomes of a cluster randomized controlled trial of the SoMe social media literacy program for improving body image-related outcomes in adolescent boys and girls. *Nutrients*. 2021;13:3825. DOI: 10.3390/nu13113825

50 Kutok ER, Dunsiger S, Patena JV, *et al.* A Cyberbullying Media-Based Prevention Intervention for Adolescents on Instagram: Pilot Randomized Controlled Trial. *JMIR Ment Health*. 2021;8:e26029. doi: 10.2196/26029

51 Schoeps K, Villanueva L, Prado-Gascó VJ, *et al.* Development of Emotional Skills in Adolescents to Prevent Cyberbullying and Improve Subjective Well-Being. *Front Psychol*. 2018;9. doi: 10.3389/fpsyg.2018.02050

52 Ofcom. A Positive Vision for Media Literacy: Ofcom's Three-Year Media Literacy Strategy. 2024.

53 Ng ED, Chua JYX, Shorey S. The effectiveness of educational interventions on traditional bullying and cyberbullying among adolescents: A systematic review and meta-analysis. *Trauma Violence Abuse*. 2022;23:132–51. https://doi.org/10.1177/1524838020933867

54 Zuair AA, Sopory P. Effects of media health literacy school-based interventions on adolescents' body image concerns, eating concerns, and thin-internalization attitudes: A systematic review and meta-analysis. *Health Commun*. 2022;37:20–8. DOI: 10.1080/10410236.2020.1813954

55 Weinstein E, James C. School-based initiatives promoting digital citizenship and healthy digital media use. *Handb Adolesc Digit Media Use Ment Health*. 2022;365. DOI: https://doi.org/10.1017/9781108976237.020

56 Moher D, Shamseer L, Clarke M, *et al.* Preferred reporting items for systematic review and meta-analysis protocols (PRISMA-P) 2015 statement. *Syst Rev*. 2015;4:1. doi: 10.1186/2046-4053-4-1

57 Page MJ, McKenzie JE, Bossuyt PM, *et al.* The PRISMA 2020 statement: an updated guideline for reporting systematic reviews. *BMJ*. 2021;372:n71. doi: 10.1136/bmj.n71

58 Haddaway NR, Grainger MJ, Gray CT. Citationchaser: A tool for transparent and efficient forward and backward citation chasing in systematic searching. *Res Synth Methods*. 2022;13:533–45. DOI: 10.1002/jrsm.1563

59 Adler A, Unanue W, Osin E, *et al.* Psychological wellbeing. *Happiness*. 2017;118.

60 Stone AA, Mackie C, Framework P on MSW-B in a P-R, *et al.* Introduction. *Subjective Well-Being: Measuring Happiness, Suffering, and Other Dimensions of Experience [Internet]*. National Academies Press (US) 2013.

61 Harvard University Health Sciences. Physical – Center for Wellness and Health Promotion. n/d. https://wellness.huhs.harvard.edu/physical

62 Hung S-T, Cheng Y-C, Wu C-C, *et al.* Examining Physical Wellness as the fundamental element for achieving holistic well-being in older persons: review of literature and

practical application in Daily Life. *J Multidiscip Healthc*. 2023;1889–904. doi: 10.2147/JMDH.S419306

63 Wallis R, Buckingham D. Media literacy: the UK's undead cultural policy. *Int J Cult Policy*. 2019;25:188–203. doi: 10.1080/10286632.2016.1229314

64 Hoffmann TC, Glasziou PP, Boutron I, *et al.* Better reporting of interventions: template for intervention description and replication (TIDieR) checklist and guide. *BMJ*. 2014;348:g1687. doi: 10.1136/bmj.g1687

65 Michie S, Richardson M, Johnston M, *et al.* The behavior change technique taxonomy (v1) of 93 hierarchically clustered techniques: building an international consensus for the reporting of behavior change interventions. *Ann Behav Med*. 2013;46:81–95. DOI: 10.1007/s12160-013-9486-6

66 Higgins J. Cochrane handbook for systematic reviews of interventions. Version 5.1. 0 [updated March 2011]. The Cochrane Collaboration. *Www Cochrane-Handb Org*. 2011.

67 Hodder RK, O'Brien KM, Lorien S, *et al.* Interventions to prevent obesity in school-aged children 6-18 years: An update of a Cochrane systematic review and meta-analysis including studies from 2015–2021. *eClinicalMedicine*. 2022;54. doi: 10.1016/j.eclinm.2022.101635

68 Zhang Q, Wang J, Neitzel A. School-based Mental Health Interventions Targeting Depression or Anxiety: A Meta-analysis of Rigorous Randomized Controlled Trials for School-aged Children and Adolescents. *J Youth Adolesc*. 2023;52:195–217. doi: 10.1007/s10964-022-01684-4

69 Popay J, Roberts H, Sowden A, *et al.* Guidance on the conduct of narrative synthesis in systematic reviews. *Prod ESRC Methods Programme Version*. 2006;1:b92. DOI: 10.13140/2.1.1018.4643

70 Rodgers M, Sowden A, Petticrew M, *et al.* Testing methodological guidance on the conduct of narrative synthesis in systematic reviews: effectiveness of interventions to promote smoke alarm ownership and function. *Evaluation*. 2009;15:49–73. https://doi.org/10.1177/1356389008097871

71 McCoy CE. Understanding the Intention-to-treat Principle in Randomized Controlled Trials. *West J Emerg Med*. 2017;18:1075–8. doi: 10.5811/westjem.2017.8.35985

72 Cheung MW-L. Modeling dependent effect sizes with three-level meta-analyses: A structural equation modeling approach. *Psychol Methods*. 2014;19:211–29. doi: 10.1037/a0032968

73 Van den Noortgate W, López-López JA, Marín-Martínez F, *et al.* Three-level meta-analysis of dependent effect sizes. *Behav Res Methods*. 2013;45:576–94. doi: 10.3758/s13428-012-0261-6

74 Borenstein M, Cooper H, Hedges L, *et al.* Effect sizes for continuous data. *Handb Res Synth Meta-Anal*. 2009;2:221–35.

For peer review only - http://bmjopen.bmj.com/site/about/guidelines.xhtml

75 Weisz JR, Kuppens S, Ng MY, *et al.* What five decades of research tells us about the effects of youth psychological therapy: A multilevel meta-analysis and implications for science and practice. *Am Psychol*. 2017;72:79–117. doi: 10.1037/a0040360

76 Pustejovsky JE, Tipton E. Meta-analysis with Robust Variance Estimation: Expanding the Range of Working Models. *Prev Sci Off J Soc Prev Res*. 2022;23:425–38. doi: 10.1007/s11121-021-01246-3

77 Cheung MW-L. metaSEM: An R package for meta-analysis using structural equation modeling. *Front Psychol*. 2015;5:1521. DOI: 10.3389/fpsyg.2014.01521

78 Core Team R. R: A language and environment for statistical computing [Software]. *R Found Stat Comput*. 2021.

79 Guest E, Zucchelli F, Costa B, *et al.* A systematic review of interventions aiming to promote positive body image in children and adolescents. *Body Image*. 2022;42:58–74. doi: 10.1016/j.bodyim.2022.04.009

80 Malinauskas R, Malinauskiene V. A meta-analysis of psychological interventions for Internet/smartphone addiction among adolescents. *J Behav Addict*. 2019;8:613–24. DOI: 10.1556/2006.8.2019.72

81 Cowling M, Sim KN, Orlando J, *et al.* Untangling Digital Safety, literacy, and Wellbeing in School activities for 10 to 13 Year Old Students. *Educ Inf Technol*. 2025;30:941–58. https://doi.org/10.1007/s10639-024-13183-z

For peer review only - http://bmjopen.bmj.com/site/about/guidelines.xhtml

Supplementary Material

Supplement 1. *Example SCOPUS Search String.*

**Wellbeing Domain**	**Search Strategy**
Psychological/Subjective Wellbeing:	TITLE-ABS-KEY ((adolescen* OR teen* OR student* OR pupil* OR youth OR "young pe*")<br>AND<br>(“digital skill*" OR "digital safety" OR "online safety" OR "online resilience" OR "digital resilience" OR "digital competenc*” OR "internet safety" OR "social media literacy" OR "digital literacy" OR "media literacy" OR "information literacy” OR "AI literacy” OR misinformation OR "social media" OR "digital media" OR "social network*" OR online)<br>AND<br>("mental health" OR "mental illness" OR "mental disorder*" OR "psychological distress" OR wellbeing* OR "mental wellbeing" OR "life satisfaction" OR affect* OR "emotional wellbeing" OR "psychological wellbeing" OR resilience OR happiness OR optimism OR "personal growth" OR "self expression" OR identity OR suicid* OR "self-

For peer review only

	harm" OR depress* OR anxi* OR stress OR internali?* OR externali?* OR "self esteem" OR "self concept" OR "self worth" OR "body image" OR "eating disorder*" OR "disordered eating")<br><br>AND<br><br>("secondary school" OR "high school" OR "middle school" OR "post primary" OR "secondary education" OR classroom* OR "school-based" OR "whole school")<br><br>AND<br><br>(experiment* OR randomi?ed OR "randomi?ed controlled trial*" OR "randomi?ed control trial*" OR rct OR "cluster rct" OR "cluster randomi?ed*" OR "quasi-experiment*" OR evaluation OR pretest OR posttest OR "pre test" OR "post test" OR lesson* OR intervention* OR effectiveness)
Social Wellbeing	TITLE-ABS-KEY ((adolescen* OR teen* OR student* OR pupil* OR youth OR "young pe*") <br>AND

For peer review only - http://bmjopen.bmj.com/site/about/guidelines.xhtml

	(“digital skill*" OR "digital safety" OR "online safety" OR "online resilience" OR "digital resilience" OR "digital competenc*” OR "internet safety" OR "social media literacy" OR "digital literacy" OR "media literacy" OR "information literacy” OR "AI literacy” OR misinformation OR "social media" OR "digital media" OR "social network*" OR online)<br><br>AND<br><br>(loneliness OR "social isolation" OR "social connect*" OR belonging OR friendship OR "social support" OR "peer rejection”)<br><br>AND

For peer review only

For peer review only - http://bmjopen.bmj.com/site/about/guidelines.xhtml

	("secondary school" OR "high school" OR "middle school" OR "post primary" OR "secondary education" OR classroom* OR "school-based" OR "whole school")<br><br>AND<br><br>(experiment* OR randomi?ed OR "randomi?ed controlled trial*" OR "randomi?ed control trial*" OR rct OR "cluster rct" OR "cluster randomi?ed*" OR "quasi-experiment*" OR evaluation OR pretest OR posttest OR "pre test" OR "post test" OR lesson* OR intervention* OR effectiveness)
Physical Wellbeing	TITLE-ABS-KEY ((adolescen* OR teen* OR student* OR pupil* OR youth OR "young pe*") <br>AND

For peer review only - http://bmjopen.bmj.com/site/about/guidelines.xhtml

	(“digital skill*" OR "digital safety" OR "online safety" OR "online resilience" OR "digital resilience" OR "digital competenc*” OR "internet safety" OR "social media literacy" OR "digital literacy" OR "media literacy" OR "information literacy” OR "AI literacy” OR misinformation OR "social media" OR "digital media" OR "social network*" OR online)<br><br>AND<br><br>(“physical wellbeing" OR "physical well-being" OR sleep* OR "sedentary behav*" OR exercise OR "substance abuse" OR "substance use" OR "physical activ*" OR "health behav*" OR "sexual health")<br><br>AND<br><br>("secondary school" OR "high school" OR "middle school" OR "post primary" OR "secondary education" OR classroom* OR "school-based" OR "whole school")

For peer review only - http://bmjopen.bmj.com/site/about/guidelines.xhtml

	AND<br><br>(experiment* OR randomi?ed OR "randomi?ed controlled trial*" OR "randomi?ed control trial*" OR rct OR "cluster rct" OR "cluster randomi?ed*" OR "quasi-experiment*" OR evaluation OR pretest OR posttest OR "pre test" OR "post test" OR lesson* OR intervention* OR effectiveness)
Digital Wellbeing	TITLE-ABS-KEY ((adolescen* OR teen* OR student* OR pupil* OR youth OR "young pe*”)<br><br>AND<br><br>(“digital skill*" OR "digital safety" OR "online safety" OR "online resilience" OR "digital resilience" OR "digital competenc*” OR "internet safety" OR "social media literacy" OR "digital literacy" OR "media literacy"

For peer review only - http://bmjopen.bmj.com/site/about/guidelines.xhtml

	OR "information literacy" OR "AI literacy" OR misinformation OR "social media" OR "digital media" OR "social network*" OR online)<br><br>AND<br><br>("digital well*" OR "online well*" OR cyberbull* OR bully* OR victimis* OR victimiz* OR harass* OR aggress* OR "online abuse" OR sext* OR groom* OR "harmful content" OR "violent content" OR "self-harm content" OR "hate speech" OR extremis* OR radicalis* OR radicaliz* OR "problematic internet*" OR "problematic social media*" OR "internet addiction" OR "social media addiction" OR "problematic smartphone*" OR "technology overuse" OR "gaming addiction" OR "problematic gaming" OR "online gambling" OR "screen time" OR "digital fatigue" OR "information overload" OR "fear of missing out" OR fomo OR pornograph* OR porn*)

For peer review only

For peer review only - http://bmjopen.bmj.com/site/about/guidelines.xhtml

	AND<br><br>("secondary school" OR "high school" OR "middle school" OR "post primary" OR "secondary education" OR classroom* OR "school-based" OR "whole school")<br><br>AND<br><br>(experiment* OR randomi?ed OR "randomi?ed controlled trial*" OR "randomi?ed control trial*" OR rct OR "cluster rct" OR "cluster randomi?ed*" OR "quasi-experiment*" OR evaluation OR pretest OR posttest OR "pre test" OR "post test" OR lesson* OR intervention* OR effectiveness)

For peer review only - http://bmjopen.bmj.com/site/about/guidelines.xhtml

Supplement 2. *Categorisation of Wellbeing Constructs for the Current Review**

**Wellbeing Domain**	**Direction of Indicator**	**Example & Definition**	**Example Representative Measures**
Psychological and Subjective Wellbeing	Positive Indicators	Life Satisfaction: Measures that assess satisfaction with life overall.	Satisfaction with Life Scale (Diener et al., 1985).
		Positive Affect: Measures that assess how much positive affect the individual experiences in their life.	PANAS–Positive Affect (Watson et al., 1988).
		Composite Wellbeing Measures which assess an individual's overall subjective wellbeing	Warwick Edinburgh Mental Wellbeing Scale (Tennant et al., 2007).

For peer review only

For peer review only - http://bmjopen.bmj.com/site/about/guidelines.xhtml

		Resilience<br><br>Measures which assess the ability to adapt and/or recover.	Adolescent Psychological Resilience Scale (APRS; Bulut et al., 2013).
		Optimism<br><br>Measures general expectations about positive outcomes.	Revised Life Orientation Test (LOT-R; Scheier et al., 1994).
		Quality of Life<br><br>Assesses overall satisfaction and fulfilment across life domains.	The Quality-of-Life Scale (QOLS; Burckhardt et al., 2003).
		Body Satisfaction<br><br>Measures contentment with various aspects of their body.	The Body Satisfaction Scale (Slade et al., 1990).
		Body Esteem<br><br>Measures positive self-evaluations of	The Body Esteem Scale (Franzoi & Shields, 1984).

For peer review only

		one's body and appearance.	
		Self Esteem<br><br>Measures an individual's overall sense of self-worth and value.	Rosenberg self-esteem scale (Rosenberg, 2011).
	Negative Indicators	DSM-V defined mental health outcomes (e.g., depression, anxiety, stress)<br><br>Measures which assess mental health outcomes as defined by the DSM-V.	Beck Depression Inventory (Beck, 1961).<br><br>State–Trait Anxiety Inventory (Spielberger, 1994)<br><br>Beck Anxiety Inventory (Beck et al., 1988).
		Negative Affect:<br><br>Measures that assess how much negative effect the individual	PANAS–Negative Affect (Watson et al., 1988).

For peer review only - http://bmjopen.bmj.com/site/about/guidelines.xhtml

		experiences in their life.	
		Body Dissatisfaction<br><br>Measures an individual's negative attitudes or feelings towards their own physical appearance.	Appearance evaluation subscale of the multidimensional body-self relations questionnaire (Cash, 2017).
Social Wellbeing	Positive Indicators	Social Connectedness<br><br>Measures perceived closeness and belonging in relationships and society.	The Social Connectedness scale (Lee & Robbins, 1995).
		Perceived Social Support<br><br>Measures the subjective feeling of having support from	Social Support Questionnaire (SSQ; Sarason et al., 1983).<br><br>The Child and Adolescent Social Support Scale.

For peer review only - http://bmjopen.bmj.com/site/about/guidelines.xhtml

		others. E.g. family, peers, and teachers.	(CASSS; Malecki et al., 2018).
	Negative Indicators	Loneliness<br><br>Measures subjective feelings of isolation and lack of social connection.	UCLA (Russell et al., 1978).
		Social Isolation<br><br>Measures lack of social networks and frequency of social interactions.	Lubben Social Network Scale (Lubben et al., 2006).
		Peer Rejection<br><br>Measures the degree to which an individual is disliked, excluded, or rejected by peers.	Social peer rejection measure (Lev-Wiesel et al., 2013).
		Cyberbullying Victimisation<br><br>Measures experiences of being targeted or harassed online.	Scale of Victimisation through the Cell Phone and Internet (Álvarez-García et al., 2017).

For peer review only

			Cyberbullying victimization (CBV) scale (Lee et al., 2017).
		Cyberbullying Perpetration<br><br>Measures engagement in online bullying behaviours, such as sending harmful messages, spreading rumours, or excluding others digitally.	Scale of Aggression through the Cell Phone and Internet (Álvarez-García et al., 2016)<br><br>Cyberbullying perpetration (CBP) scale (Lee et al., 2017)
Physical Wellbeing	Positive/Negative Indicators	Self-Reported Health Status<br><br>Measures perceived overall general health.	Self-rated health (SRH; Bombak, 2013).
		Sleep Quality<br><br>Measures satisfaction with sleep and restfulness.	Pittsburgh Sleep Quality Index (Buysse et al., 1989).
		Substance Use Behaviours	Risk Behaviour Index–5 items on tobacco, alcohol, and

For peer review only - http://bmjopen.bmj.com/site/about/guidelines.xhtml

		Measures risk behaviours related to tobacco, alcohol, and drug use.	drug use (Williams et al., 2000).
		Physical Activity<br><br>Measures frequency and intensity of exercise.	Physical Activity Questionnaire for Adolescents (PAQ-A; Kowalski et al., 1997).
	Negative Indicators	Perceived/Self-Reported Sleep Problems<br><br>Measures difficulties falling asleep, staying asleep, or feeling rested, based on self-report.	Adolescent Sleep Hygiene Scale (ASHS; LeBourgeois et al., 2005).
		Sedentary Behaviour<br><br>Measures time spent sitting or engaging in low-energy activities.	Sedentary Behavior Questionnaire (SBQ; Rosenberg et al., 2010).

For peer review only

	Positive	Health-Related Quality of Life<br><br>Measures the impact of various dimensions of health on quality of life.	SF-36 (Short Form 36 Health Survey; Ware & Sherbourne, 1992).
Digital-Specific Wellbeing	Negative	Social media addiction<br><br>Measures problematic or compulsive use of social media.	The Bergen Facebook Addiction Scale (BFAS) (Bowman & Clark-Gordon, 2019).
		Problematic social media use<br><br>Measures negative patterns of use which disrupt functioning.	Social Media Disorder scale (Boer et al., 2022).<br><br>Bergen Social Media Addiction Scale (BSMAS; Lin et al., 2017).
		Online gambling behaviour<br><br>Measures problem gambling and related	Adolescent Gambling Inventory's (CAGI) Problem Gambling Severity Index (PGSI; Tremblay et al., 2010).

For peer review only - http://bmjopen.bmj.com/site/about/guidelines.xhtml

		harms in online settings.	
		Online financial risk behaviours<br><br>Measures risky financial actions online and related consequences.	DOSPERT - Financial domain specific (Weber et al., 2002).
		Compulsive tech use<br><br>Measures excessive or uncontrolled use of digital devices or the internet leading to negative consequences/ functional impairment.	The Problematic and Risky Internet Use Screening Scale (PRIUSS; Jelenchick et al., 2014).

6 *Illustrative examples for the purposes of the protocol.

For peer review only - http://bmjopen.bmj.com/site/about/guidelines.xhtml

Supplement 3. *Categorisation of Media and Digital Literacy Outcomes for the Current Review**

Domain	Definition	Example of Measures
Functional Skills	The skills/practical ability to access and use devices and digital technologies.	• Internet skills for school (ISFS; Kuhlemeier & Hemker, 2007).<br>• iKnow measure (internet knowledge), (Potosky, 2007).<br>• yDSI skills, content creation & production dimension (Helsper et al., 2021).
Critical Skills	The ability to evaluate, assess and analyse media in a critical way.	• Media Literacy Scale (Arke & Primack, 2009).<br>• MeHLit – Media Health Literacy Scale (Nazarnia et al., 2022).<br>• Media Attitudes Questionnaire-Realism Scepticism (Irving et al., 1998).<br>• Critical Processing of Beauty Images Scale (CPBI; Engeln-Maddox & Miller, 2008).<br>• The Critical Thinking about Media Messages (CTMM) scale, (McLean et al., 2016).

For peer review only

Creative Production	The ability to create, develop and distribute various forms of media/content.	• yDSI skills, content creation & production dimension (Helsper et al., 2021).

*Illustrative examples for the purposes of the protocol.

For peer review only

For peer review only - http://bmjopen.bmj.com/site/about/guidelines.xhtml

PRISMA-P (Preferred Reporting Items for Systematic review and Meta-Analysis Protocols) 2015 checklist: recommended items to address in a systematic review protocol*

Section and topic	Item No	Checklist item	Location where item is reported
**ADMINISTRATIVE INFORMATION**			
Title:			
Identification	1a	Identify the report as a protocol of a systematic review	Pg. 1
Update	1b	If the protocol is for an update of a previous systematic review, identify as such	n/a
Registration	2	If registered, provide the name of the registry (such as PROSPERO) and registration number	Pg. 9
Authors:			
Contact	3a	Provide name, institutional affiliation, e-mail address of all protocol authors; provide physical mailing address of corresponding author	Pg. 1
Contributions	3b	Describe contributions of protocol authors and identify the guarantor of the review	Pg.1
Amendments	4	If the protocol represents an amendment of a previously completed or published protocol, identify as such and list changes; otherwise, state plan for documenting important protocol amendments	n/a
Support:			
Sources	5a	Indicate sources of financial or other support for the review	Pg. 25
Sponsor	5b	Provide name for the review funder and/or sponsor	Pg. 25
Role of sponsor or funder	5c	Describe roles of funder(s), sponsor(s), and/or institution(s), if any, in developing the protocol	Pg. 25
**INTRODUCTION**			
Rationale	6	Describe the rationale for the review in the context of what is already known	Pg. 4 - 8
Objectives	7	Provide an explicit statement of the question(s) the review will address with reference to participants, interventions, comparators, and outcomes (PICO)	Pg. 8
**METHODS**			
Eligibility criteria	8	Specify the study characteristics (such as PICO, study design, setting, time frame) and report characteristics (such as years considered, language, publication status) to be used as criteria for eligibility for the review	Pg. 11 - 18
Information sources	9	Describe all intended information sources (such as electronic databases, contact with study authors, trial registers or other grey literature sources) with planned dates of coverage	Pg. 9
Search strategy	10	Present draft of search strategy to be used for at least one electronic database, including planned limits, such that it could be repeated	Supplements

For peer review only

Study records:			
Data management	11a	Describe the mechanism(s) that will be used to manage records and data throughout the review	Pg. 19
Selection process	11b	State the process that will be used for selecting studies (such as two independent reviewers) through each phase of the review (that is, screening, eligibility and inclusion in meta-analysis)	Pg. 19
Data collection process	11c	Describe planned method of extracting data from reports (such as piloting forms, done independently, in duplicate), any processes for obtaining and confirming data from investigators	Pg. 19 - 20
Data items	12	List and define all variables for which data will be sought (such as PICO items, funding sources), any pre-planned data assumptions and simplifications	Pg. 20 21
Outcomes and prioritization	13	List and define all outcomes for which data will be sought, including prioritization of main and additional outcomes, with rationale	Pg. 21
Risk of bias in individual studies	14	Describe anticipated methods for assessing risk of bias of individual studies, including whether this will be done at the outcome or study level, or both; state how this information will be used in data synthesis	Pg. 21
Data synthesis	15a	Describe criteria under which study data will be quantitatively synthesised	Pg. 22
	15b	If data are appropriate for quantitative synthesis, describe planned summary measures, methods of handling data and methods of combining data from studies, including any planned exploration of consistency (such as I^2, Kendall's τ)	Pg. 22 - 24
	15c	Describe any proposed additional analyses (such as sensitivity or subgroup analyses, meta-regression)	Pg. 23 - 24
	15d	If quantitative synthesis is not appropriate, describe the type of summary planned	n/a
Meta-bias(es)	16	Specify any planned assessment of meta-bias(es) (such as publication bias across studies, selective reporting within studies)	Pg. 21
Confidence in cumulative evidence	17	Describe how the strength of the body of evidence will be assessed (such as GRADE)	n/a

*** It is strongly recommended that this checklist be read in conjunction with the PRISMA-P Explanation and Elaboration (cite when available) for important clarification on the items. Amendments to a review protocol should be tracked and dated.** **The copyright for PRISMA-P (including checklist) is held by the PRISMA-P Group and is distributed under a Creative Commons Attribution Licence 4.0.**

From: Shamseer L, Moher D, Clarke M, Ghersi D, Liberati A, Petticrew M, Shekelle P, Stewart L, PRISMA-P Group. Preferred reporting items for systematic review and meta-analysis protocols (PRISMA-P) 2015: elaboration and explanation. BMJ. 2015 Jan 2;349(jan02 1):g7647.

For peer review only
